# Supplementary material for: Interventions to reintroduce or increase assisted vaginal births: a systematic review of the literature
Source: BMJ Open. 2023 Feb 14;13(2):e070640. doi: 10.1136/bmjopen-2022-070640 (PMC9930566; doi:10.1136/bmjopen-2022-070640)
Supplement: Supplementary data [file bmjopen-2022-070640supp001.pdf]

Supplementary file 1. Search strategy

Initial searches December 31, 2019. Updated on September 1, 2021

MEDLINE / PubMed

Searches
#1 "operative vaginal delivery" OR "operative vaginal deliver*" OR OVD [ti]<br>#2 "assisted vaginal delivery" OR "assisted vaginal deliver*" OR AVD [ti]<br>#3 "assisted vaginal birth" OR AVB [ti]<br>#4 "instrumental delivery" OR "instrumental deliver*" OR "instrumental vaginal delivery" OR "instrumental vaginal deliver*" [ti]<br>#5 "kiwi delivery" OR "kiwi deliver*" [ti]<br>#6 "ventouse delivery" OR "ventouse deliver*" OR "ventouse assisted delivery" OR "ventouse assisted deliver*" [ti]<br>#7 "vacuum delivery" OR "vacuum deliver*" OR "vacuum assisted delivery" OR "vacuum assisted deliver*" [ti]<br>#8 "forceps delivery" OR "forceps deliver*" OR "forceps assisted delivery" OR "forceps assisted deliver*" [ti]<br>#9 "obstetric forceps" OR "obstetrical forceps" [ti]<br>#10 ventouse OR "vacuum extraction" OR "vacuum extractor" [ti]<br>#11 #1 OR #2 OR #3 OR #4 OR #5 OR #6 OR #7 OR #8 OR #9 OR #10

EMBASE

Searches
#1 "operative vaginal delivery" [exp]<br>#2 "operative vaginal delivery" OR "operative vaginal deliver*" OR OVD [ti]<br>#3 "assisted vaginal delivery" [exp]<br>#4 "assisted vaginal delivery" OR "assisted vaginal deliver*" OR AVD [ti]<br>#5 "assisted vaginal birth" OR AVB [ti]<br>#6 "instrumental delivery" [exp] ('forceps delivery'/exp OR 'vacuum extraction'/exp)<br>#7 "instrumental delivery" OR "instrumental deliver*" OR "instrumental vaginal delivery" OR "instrumental vaginal deliver*" [ti]<br>#8 "kiwi delivery" OR "kiwi deliver*" [ti]<br>#9 "ventouse delivery" OR "ventouse deliver*" OR "ventouse assisted delivery" OR "ventouse assisted deliver*" [ti]<br>#10 "obstetric vacuum delivery kit" [exp]<br>#11 "obstetric vacuum delivery kit" OR "vacuum delivery" OR "vacuum deliver*" OR "vacuum assisted delivery" OR "vacuum assisted deliver*" [ti]<br>#12 "forceps delivery" [exp]<br>#13 "forceps delivery" OR "forceps deliver*" OR "forceps assisted delivery" OR "forceps assisted deliver*" [ti]<br>#14 "obstetric forceps" [exp]<br>#15 "obstetric forceps" OR "obstetrical forceps" [ti]<br>#16 "vacuum extraction" OR "vacuum extractor" [exp]<br>#17 ventouse OR "vacuum extraction" OR "vacuum extractor" [ti]<br>#18 #1 OR #2 OR #3 OR #4 OR #5 OR #6 OR #7 OR #8 OR #9 OR #10 OR #11 OR #12 OR #13 OR #14 OR #15 OR #16 OR #17

Torloni MR, *et al. BMJ Open* 2023; 13:e070640. doi: 10.1136/bmjopen-2022-070640

CINAHL

Searches
#1 “vaginal birth” [SU]<br>#2 “operative vaginal delivery” OR “operative vaginal deliver*” OR OVD [ti]<br>#3 “assisted vaginal delivery” OR “assisted vaginal deliver*” OR AVD [ti]<br>#4 “assisted vaginal birth” OR AVB [ti]<br>#5 “instrumental delivery” OR “instrumental deliver*” OR “instrumental vaginal delivery” OR “instrumental vaginal deliver*” [ti]<br>#6 “kiwi delivery” OR “kiwi deliver*” [ti]<br>#7 “ventouse delivery” OR “ventouse deliver*” OR “ventouse assisted delivery” OR “ventouse assisted deliver*” [ti]<br>#8 “vacuum delivery” OR “vacuum deliver*” OR “vacuum assisted delivery” OR “vacuum assisted deliver*” [ti]<br>#9 “forceps delivery” OR “forceps deliver*” OR “forceps assisted delivery” OR “forceps assisted deliver*” [ti]<br>#10 “obstetrical forceps” [SU]<br>#11 “obstetric forceps” OR “obstetrical forceps” [ti]<br>#12 “vacuum extraction, obstetrical” [SU]<br>#13 ventouse OR “vacuum extraction” OR “vacuum extractor” [ti]<br>#14 #1 OR #2 OR #3 OR #4 OR #5 OR #6 OR #7 OR #8 OR #9 OR #10 OR #11 OR #12 OR #13

LILACS

Searches
#1 “operative vaginal delivery” OR “operative vaginal deliver*” OR OVD [ti]<br>#2 “assisted vaginal delivery” OR “assisted vaginal deliver*” OR AVD [ti]<br>#3 “assisted vaginal birth” OR AVB [ti]<br>#4 “instrumental delivery” OR “instrumental deliver*” [ti]<br>#5 “instrumental vaginal delivery” OR “instrumental vaginal deliver*” [ti]<br>#6 “kiwi delivery” OR “kiwi deliver*” [ti]<br>#7 “ventouse delivery” OR “ventouse deliver*” [ti]<br>#8 “ventouse assisted delivery” OR “ventouse assisted deliver*” [ti]<br>#9 “vacuum delivery” OR “vacuum deliver*” [ti]<br>#10 “vacuum assisted delivery” OR “vacuum assisted deliver*” [ti]<br>#11 “forceps delivery” OR “forceps deliver*” [ti]<br>#12 “forceps assisted delivery” OR “forceps assisted deliver*” [ti]<br>#13 “obstetric forceps” OR “obstetrical forceps” [ti]<br>#14 ventouse OR “vacuum extraction” OR “vacuum extractor” [ti]

SCOPUS

Searches
#1 “operative vaginal delivery” OR “operative vaginal deliver*” OR OVD [ti]<br>#2 “assisted vaginal delivery” OR “assisted vaginal deliver*” OR AVD [ti]<br>#3 “assisted vaginal birth” OR AVB [ti]<br>#4 “instrumental delivery” OR “instrumental deliver*” OR “instrumental vaginal delivery” OR “instrumental vaginal deliver*” [ti]<br>#5 “kiwi delivery” OR “kiwi deliver*” [ti]<br>#6 “ventouse delivery” OR “ventouse deliver*” OR “ventouse assisted delivery” OR “ventouse assisted deliver*” [ti]<br>#7 “vacuum delivery” OR “vacuum deliver*” OR “vacuum assisted delivery” OR “vacuum assisted deliver*” [ti]<br>#8 “forceps delivery” OR “forceps deliver*” OR “forceps assisted delivery” OR “forceps assisted deliver*” [ti]<br>#9 “obstetric forceps” OR “obstetrical forceps” [ti]<br>#10 ventouse OR “vacuum extraction” OR “vacuum extractor” [ti]<br>#11 #1 OR #2 OR #3 OR #4 OR #5 OR #6 OR #7 OR #8 OR #9 OR #10

Cochrane Library

Searches
#1 “operative vaginal delivery” OR “operative vaginal deliver*” OR OVD [ti]<br>#2 “assisted vaginal delivery” OR “assisted vaginal deliver*” OR AVD [ti]<br>#3 “assisted vaginal birth” OR AVB [ti]<br>#4 “instrumental delivery” OR “instrumental deliver*” OR “instrumental vaginal delivery” OR “instrumental vaginal deliver*” [ti]<br>#5 “kiwi delivery” OR “kiwi deliver*” [ti]<br>#6 “ventouse delivery” OR “ventouse deliver*” OR “ventouse assisted delivery” OR “ventouse assisted deliver*” [ti]<br>#7 “vacuum delivery” OR “vacuum deliver*” OR “vacuum assisted delivery” OR “vacuum assisted deliver*”<br>#8 “forceps delivery” OR “forceps deliver*” OR “forceps assisted delivery” OR “forceps assisted deliver*” [ti]<br>#9 “obstetrical forceps” [MeSH]<br>#10 “obstetric forceps” OR “obstetrical forceps” [ti]<br>#11 “vacuum extraction, obstetrical” [MeSH]<br>#12 ventouse OR “vacuum extraction” OR “vacuum extractor” [ti]<br>#13 #1 OR #2 OR #3 OR #4 OR #5 OR #6 OR #7 OR #8 OR #9 OR #10 OR #11 OR #12

WHO Library

Searches
#1 “operative vaginal delivery” OR “operative vaginal deliver*” OR OVD [ti]<br>#2 “assisted vaginal delivery” OR “assisted vaginal deliver*” OR AVD [ti]<br>#3 “assisted vaginal birth” OR AVB [ti]<br>#4 “instrumental delivery” OR “instrumental deliver*” [ti]<br>#5 “instrumental vaginal delivery” OR “instrumental vaginal deliver*” [ti]<br>#6 “kiwi delivery” OR “kiwi deliver*” [ti]<br>#7 “ventouse delivery” OR “ventouse deliver*” [ti]<br>#8 “ventouse assisted delivery” OR “ventouse assisted deliver*” [ti]<br>#9 “vacuum delivery” OR “vacuum deliver*” [ti]<br>#10 “vacuum assisted delivery” OR “vacuum assisted deliver*” [ti]<br>#11 “forceps delivery” OR “forceps deliver*” [ti]<br>#12 “forceps assisted delivery” OR “forceps assisted deliver*” [ti]<br>#13 “obstetric forceps” OR “obstetrical forceps” [ti]<br>#14 ventouse OR “vacuum extraction” OR “vacuum extractor” [ti]<br>#15 #1 OR #2 OR #3 OR #4 OR #5 OR #6 OR #7 OR #8 OR #9 OR #10 OR #11 OR #12 OR #13 OR #14

Web of science

Searches
#1 “operative vaginal delivery” OR “operative vaginal deliver*” OR OVD [ti]<br>#2 “assisted vaginal delivery” OR “assisted vaginal deliver*” OR AVD [ti]<br>#3 “assisted vaginal birth” OR AVB [ti]<br>#4 “instrumental delivery” OR “instrumental deliver*” OR “instrumental vaginal delivery” OR “instrumental vaginal deliver*” [ti]<br>#5 “kiwi delivery” OR “kiwi deliver*” [ti]<br>#6 “ventouse delivery” OR “ventouse deliver*” OR “ventouse assisted delivery” OR “ventouse assisted deliver*” [ti]<br>#7 “vacuum delivery” OR “vacuum deliver*” OR “vacuum assisted delivery” OR “vacuum assisted deliver*” [ti]<br>#8 “forceps delivery” OR “forceps deliver*” OR “forceps assisted delivery” OR “forceps assisted deliver*” [ti]<br>#9 “obstetric forceps” OR “obstetrical forceps” [ti]<br>#10 ventouse OR “vacuum extraction” OR “vacuum extractor” [ti]<br>#11 #1 OR #2 OR #3 OR #4 OR #5 OR #6 OR #7 OR #8 OR #9 OR #10

Torloni MR, *et al. BMJ Open* 2023; 13:e070640. doi: 10.1136/bmjopen-2022-070640

MEDLINE/PubMed- Training programs

Search terms
(RCOG Operative vaginal Birth Simulation Training OR ROBuST) OR<br>(Advanced Life Support in Obstetrics OR ALSO) OR<br>(Programa de Rescate Obstétrico y Neonatal: Tratamiento Óptimo y Oportuno OR PRONTO) OR<br>(Essential Steps in the Management of Obstetric Emergencies OR ESMOE) OR<br>(assisted vaginal birth OR operative vaginal birth OR instrumental vaginal delivery OR forceps delivery OR vacuum delivery)

Torloni MR, *et al. BMJ Open* 2023; 13:e070640. doi: 10.1136/bmjopen-2022-070640
