# Supplementary material for: Interventions to reintroduce or increase assisted vaginal births: a systematic review of the literature
Source: BMJ Open. 2023 Feb 14;13(2):e070640. doi: 10.1136/bmjopen-2022-070640 (PMC9930566; doi:10.1136/bmjopen-2022-070640)
Supplement: Supplementary data [file bmjopen-2022-070640supp002.pdf]

Supplementary file 2. Studies excluded

List of 32 studies identified through electronic search and excluded after full-text reading

Study reference	Reason for exclusion	Category for exclusion
Becker, D. A.; Blanchard, C. T.; Szychowski, J. M.; Rogers, S. L.; Brumfield, C. G.; Subramaniam, A. 743: The effect of an operative vaginal delivery curriculum for OBGYN residents American Journal of Obstetrics and Gynecology 2019;220(1):S488	Conference abstract of study later published and included (Becker 2020)	Abstract
Beneru, D.; Mcgee, T.; Jenkins, G. Caesarean sections at full dilatation before and after mandatory consultant presence. Australian and New Zealand Journal of Obstetrics and Gynaecology 2021;61(SUPPL 1):6	Conference abstract only	Abstract
Caughey, A.; Cheng, Y. W.; Zlatnik, M.; Nguy, M.; Nguyen, L.; Thiet, M. P. Perineal lacerations and operative vaginal delivery: Results from a quality improvement program American Journal of Obstetrics and Gynecology Dec 2007;197(6):S79-S79	Conference abstract only	Abstract
Coleman, L.; Basude, S. A simple program of focused education and simulation training to reduce the rate of obstetric anal sphincter injury at forceps delivery. BJOG. Apr 2015;122():78-78	Conference abstract only	Abstract
Fox, Nathan S.; Bardos, Jonah; Loudon, Holly; Rekawek, Patricia; Friedman, Frederick; Brodman, Michael. 523: Adding senior obstetrician supervision increases the forceps delivery rate and decreases the cesarean delivery rate for residents. American Journal of Obstetrics and Gynecology 2017; 216(1S):S309	Conference abstract only	Abstract
Halawani, M.; Webster, S.; Soliman, E.; El-Nouri, A. Reintroduction of operative vaginal delivery at a large tertiary referral hospital in Cairo, Egypt. International Journal of Gynecology and Obstetrics 2018;143:279	Conference abstract only	Abstract
Hodges, R.; Skinner, S.; Wallace, E.; Davies, M. Perinatal and maternal outcomes after training obstetric residents in forceps before vacuum operative birth. American Journal of Obstetrics and Gynecology 2016;214(1):S270-S271	Conference abstract of study later published and included (Skinner 2017)	Abstract
Jovic, E.; Sethna, F. Does consultant attendance for a second-stage delivery in theatre make a difference? Australian and New Zealand Journal of Obstetrics and Gynaecology 2021;61(SUPPL 1):71-72	Conference abstract only	Abstract
Muller, B.; Gilreath, N.; Wineland, R.; Sullivan, S.; Finneran, M.M. Impact of contemporary labor curves on primary cesarean delivery at a single institution American Journal of Obstetrics and Gynecology 2021;224(2):S386	Conference abstract only	Abstract
Negi, Masaru MD; Espinal, Mariana MD; Holveck, Beth RN; Mehlhaff, Krista MD; Cron, Julia MD; Pettker, Christian Michael MD Improvement of Operative Vaginal Delivery Training in Residency: A Single Institution Experience [17G], Obstetrics & Gynecology: May 2020 - Volume 135 - Issue - p 75S	Conference abstract only	Abstract
Russell, M.; Esen, U. Achieving safety and success of operative vaginal deliveries at a district teaching hospital - A triumph of audit and a targeted practical teaching workshop. International Journal of Gynecology and Obstetrics 2009;107(S2):S548	Conference abstract only	Abstract

Stephens-Hennessy, Beth M. Implementation of the California Maternal Quality Care Collaborative Toolkit to Support Vaginal Birth and Reduce Primary Cesarean Births JOGNN: Journal of Obstetric, Gynecologic & Neonatal Nursing 2018;47(3):S51-S52	Conference abstract only	Abstract
Wilkinson, M; Mumtaz, H; Nassar, M; Gopal, G. Does consultant presence at trials of operative vaginal delivery increase success? BJOG- 2021;128(S2):152	Conference abstract only	Abstract
Zaid, R. Z.; Yoong, W.; Lhodi, W.; Hirsch, M.; Anderson, K.; Balghari, Z.; Aziz, A.; Relph, S.; Sivashanmugarajan, V.; Govind, A. Delivering a modular, concise training package for instrumental vaginal deliveries. BJOG 2013;120(S2):594	Conference abstract only	Abstract
Dietz, Hans Peter; Ka Lai, Shek; Callaghan, Sascha; Shek, Ka Lai. Perinatal and Maternal Outcomes After Training Residents in Forceps Before Vacuum Instrumental Birth. Obstetrics & Gynecology 2017;130(4):910-910	Letter commenting study by Skinner 2017	Letter
Spencer, C.; Murphy, D.; Bewley, S. Caesarean delivery in the second stage of labour: better training in instrumental delivery may reduce rates. BMJ 2006;333(7569):613-614	Letter	Letter
van Tetering AAC, van Meurs A, Ntuyo P, van der Hout-van der Jagt MB, Mulders LGM, Nolens B, Namagambe I, Nakimuli A, Byamugisha J, Oei SG. Study protocol training for life: a stepped wedge cluster randomized trial about emergency obstetric simulation-based training in a low-income country. BMC Pregnancy Childbirth. 2020 Jul 28;20(1):429	Study protocol	Protocol
Ayala NK, Schlichting LE, Kole MB, Clark MA, Vivier PM, Viner-Brown SI, Werner EF. Operative vaginal delivery and third grade educational outcomes.Am J Obstet Gynecol MFM. 2020 Nov;2(4):100221.	Not a training or QI initiative to increase AVB	Wrong Intervention
Chang, Xavier; Chedraui, Peter; Ross, Michael G.; Hidalgo, Luis; Penafiel, Jaime Vacuum assisted delivery in Ecuador for prolonged second stage of labor: maternal-neonatal outcome The journal of maternal-fetal & neonatal medicine. 2007 2007;20(5):381-4	Not a training or QI initiative	Wrong Intervention
Chen CC, Lee JF. Effectiveness of the doula program in Northern Taiwan. Tzu Chi Med J. 2020 Apr 1;32(4):373-379.	Not a training or QI initiative to increase AVB	Wrong Intervention
Davison, Margaret Anne; Murray, Sarah; Whitaker, Lucy; Rendall, Lesley; Gammie, Nicky; Magowan, Brian. Comparison of instrumental vaginal births by assisted birth practitioner midwives and medical practitioners. British Journal of Midwifery 2014;22(10):700-705	Not a training or QI initiative to increase AVB	Wrong Intervention
Ghi T, Rizzo G; EGEO Group. The use of a hybrid mannequin for the modern high-fidelity simulation in the labor ward: the Italian experience of the Ecografia Gestione Emergenze Ostetriche (EGEO) group. Am J Obstet Gynecol. 2020 Jan;222(1):41-47.	Not a training or QI initiative to increase AVB	Wrong Intervention
Hildebrand E, Nelson M, Blomberg M. Long-term effects of the nine-item list intervention on obstetric and neonatal outcomes in Robson group 1 - A time series study. Acta Obstet Gynecol Scand. 2021 Jan;100(1):154-161.	Not a training or QI initiative to increase AVB	Wrong Intervention
Hinkson L, Henrich W, Tutschek B. Intrapartum ultrasound during rotational forceps delivery: a novel tool for safety, quality control, and teaching. Am J Obstet Gynecol. 2021 Jan;224(1):93.e1-93.e7.	Not a training or QI initiative to increase AVB	Wrong Intervention

Romero S, Pettersson K, Yousaf K, Westgren M, Ajne G. Perinatal outcome after vacuum assisted delivery with digital feedback on traction force; a randomised controlled study. BMC Pregnancy Childbirth. 2021 Feb 26;21(1):165. doi: 10.1186/s12884-021-03604-z. PMID: 33637058; PMCID: PMC7913459.	Not a training or QI initiative to increase AVB	Wrong Intervention
Waller-Wise R, Lewis S, Williams B. A Quality Improvement Project Utilizing a Clinical Practice Guideline in Women During Second-Stage Labor. J Perinat Educ. 2020 Apr 1;29(2):72-82. doi: 10.1891/J-PE-D-19-00014. PMID: 32308356; PMCID: PMC7159796.	Not a training or QI initiative to increase AVB	Wrong Intervention
Cheong, Y. C.; Abdullahi, H.; Lashen, H.; Fairlie, F. M. Can formal education and training improve the outcome of instrumental delivery? European Journal of Obstetrics and Gynecology and Reproductive Biology 2004;113(2):139-144	Increase of AVB use was not one of the Objectives or cited as one of the Outcomes in Methods	Wrong Outcome
Chikazawa, K.; Takagi, K.; Takahashi, H.; Akashi, K.; Nakamura, E.; Samejima, K.; Ushijima, J.; Horiuchi, I. Introduction of forceps delivery education for residents at a single perinatal institution. Hypertension Research in Pregnancy 2016;4(2):102-105	No data on AVB use before x after intervention	Wrong Outcome
Coste Mazeau, P; Boukeffa, N; Ticaud Boileau, N; Huet, S; Traverse, M; Eyraud, JL; Laguerre, A; Catalan, C; Riedl, C Evaluation of Suzor forceps training by studying obstetric anal sphincter injuries: a retrospective study. BMC Pregnancy Childbirth 20, 674 (2020)	No data on AVB use before x after intervention	Wrong Outcome
Dolo O, Clack A, Gibson H, Lewis N, Southall DP. Training of midwives in advanced obstetrics in Liberia. Bull World Health Organ. 2016 May 1;94(5):383-7.	No data on AVB use before x after intervention	Wrong Outcome
Egami N, Muta R, Anami A, Koga H. Impact of clinical practice guidelines for vacuum-assisted delivery on maternal and neonatal outcomes in Japan: A single-center observational study. J Obstet Gynaecol Res. 2021 Jan;47(1):167-173	No data on AVB use before x after intervention (assessed only success rates).	Wrong Outcome
van Tetering AAC, Segers MHM, Ntuyo P, Namagambe I, van der Hout-van der Jagt MB, Byamugisha JK, Oei SG. Evaluating the Instructional Design and Effect on Knowledge, Teamwork, and Skills of Technology-Enhanced Simulation-Based Training in Obstetrics in Uganda: Stepped-Wedge Cluster Randomized Trial. JMIR Med Educ. 2021 Feb 5;7(1):e17277	Present only Kirpatrick 1 and 2 outcomes	Wrong outcome
