# Supplementary material for: Interventions to reintroduce or increase assisted vaginal births: a systematic review of the literature
Source: BMJ Open. 2023 Feb 14;13(2):e070640. doi: 10.1136/bmjopen-2022-070640 (PMC9930566; doi:10.1136/bmjopen-2022-070640)
Supplement: Supplementary data [file bmjopen-2022-070640supp003.pdf]

Supplementary file 3. Details of 16 studies on interventions to increase AVB use

Author	Study design / Year study conducted / Level of significance / Adjustments	Country / Setting	Population / Number of deliveries	Intervention strategy / Frequency and duration	Effect of intervention (Kirkpatrick levels 3 or 4)
Ameh 2014	Cluster-randomised trial (stepped wedge)<br><br>2009-2011<br><br>P < 0.05<br><br>Adjustment for clustering	Kenya<br><br>2 "level 5" hospitals and 8 regional hospitals<br><br>All health care facilities involved were provincial "referral hospitals"	All women (N=16,794 at baseline, N=17,404 intervention period Total N= 34,198)	Multifaceted intervention comprising training in emergency obstetric and neonatal care (EmONC), provision of EmOC equipment (including reusable Kiwi omnicups) and local supportive supervision.<br><br>Duration of training: 3 days	• Significant increase in vacuum extraction (from 0.2% to 1% at 12 months) P< 0.001<br>• No significant change in overall CS (baseline 21.5% vs. post-intervention 21.5%)<br>• No significant change in direct obstetric case fatality rate (from 3% to 2%)<br>• Significant decrease in NICU admission due to birth asphyxia (48% mean reduction at 12 months, from 7.6% to 4%) P = 0.03<br>• Significant decrease in stillbirth rate at 12 months (35% mean reduction at 12 months, from 5% to 3%) P = 0.03<br>• No significant change in fresh stillbirth rate (from 2.1% to 1.9%)
Bardos 2017	Before and after study<br><br>2011-2015<br><br>P< 0.05<br>Confounding variables included in multivariable logistic regression: maternal age $\geq$ 35, maternal obesity (BMI$\geq$ 35 Kg/m2 at delivery), induction of labor, parity, and prior cesarean delivery. The last two variables were not included in the analysis of nulliparous patients only.	USA<br><br>1 Teaching hospital	Term, singleton, cephalic births cared by residents (N=1919 at baseline, N=3282 intervention period, Total N=5201)	Senior obstetrician (>20 years experience) teaching and supervising residents on forceps deliveries during the day time.<br><br>Frequency and duration not stated.	• Significant increase in forceps deliveries (0.6% to 2.6%, aOR 8.44, 95% CI 3.1 to 23.1)<br>• No significant change in vacuum deliveries (3.3% to 3.3%)<br>• Significant decrease in overall CS (27.3% to 24.5%, aOR 0.68, 95% CI 0.55 to 0.83)<br>• No significant change in:<br>-3rd-4th degree tears (1.4% to 2.0%)<br>-5 min Apgar score < 7 (0.3% to 0.5%)<br>The increase in forceps deliveries and the decrease in CS were seen only in daytime hours (7 AM to 7 PM), that is, the shift that was covered by senior obstetricians

**Author**	**Study design / Year study conducted/ Level of significance / Adjustments**	**Country / Setting**	**Population / Number of deliveries**	**Intervention strategy / Frequency and duration**	**Effect of intervention** (Kirkpatrick levels 3 or 4)
Becker 2020	Retrospective cohort study<br><br>2011-2018<br><br>P< 0.05<br><br>No adjustments	USA<br><br>1 Teaching hospital	All women with births after 20 weeks gestation (N=20,471 at baseline, N=6106 intervention period, Total N=26,577)	Twice-yearly AVB residency curriculum consisting of didactics (pretest and posttest of basic AVB knowledge, history, indications, safe use, risks of forceps and vacuum) and hands-on simulation of AVB.<br><br>Frequency and duration: One 2-hour workshop offered every 6 months	• Significant increase in forceps use, mean (SD), pre-1.2% (0.6) vs. post 2.0% (0.9), P = 0.027<br>• Significant decrease in vacuum use, mean (SD), pre-2.0% (0.5) vs. post-1.2% (0.3), P < 0.001<br>• No significant change in overall AVB, mean (SD), pre-3.2% (0.8) vs. post-3.1% (1.0)<br>• Increase in overall CS rates, from 27.9%-30.6% in pre-intervention years to 33.5%-33.9% in post-intervention years (no statistical analyses)

**Author**	**Study design / Year study conducted/ Level of significance / Adjustments**	**Country / Setting**	**Population / Number of deliveries**	**Intervention strategy / Frequency and duration**	**Effect of intervention** (Kirkpatrick levels 3 or 4)
Berglund 2010	Before and after study<br><br>2003-2006<br><br>P< 0.05<br><br>No adjustments	Ukraine<br><br>3 Maternity hospitals (Donetsk, Lutsk, Lviv)	All women (in the 3 sites combined: N=1696 at baseline, N= 6710 intervention period, Total N= 8406)	Development of obstetrical and neonatal protocols, staff training on Effective Perinatal Care (EPC), continuous monitoring and evaluation (with feedback to staff), revision of university curricula to include evidence-based perinatal care, provision of equipment, resuscitation and neonatal care.<br><br>Frequency and duration:<br>Initial (EPC) training: two weeks<br>Follow up EPC training: three days	• Significant decrease in overall AVB in one maternity:<br>Donetsk: from 3.7% to 0% P<0.0001<br>• No change in overall AVB in the other two maternities:<br>Lutsk: from 2.0% to 2.0%<br>Lviv: from 0% to 0%<br>• Significant decrease in CS in two maternities:<br>Donetsk: from 30% to 18.4% P<0.0001<br>Lutsk: from 33% to 12.7% P<0.0001<br>• No change in CS in one maternity:<br>Lviv: from 21.9% to 16.9%<br>• Significant decrease in episiotomies:<br>Donetsk: from 36.8%to 4.0% P<0.0001<br>Lutsk: from 19.4% to 5.7% P<0.0001<br>Lviv: 22.2% to 7.0% P<0.0001<br>• No effect on early neonatal mortality:<br>Donetsk: from 13.6% to 9.5%<br>Lutsk: from 4.6% to 3.0%<br>Lviv: from 6.4% to 0%<br>• Significant decrease in babies needing resuscitation:<br>Donetsk: from 13.1% to 7.4% P = 0.0025<br>Lutsk: from 2.4% to 0.9% P<0.0001<br>Lviv: 4.5% to 1.1% P=0.0003<br>• Significant decrease in NICU admission in two maternities:<br>Lutsk: from 7.3% to 4.4% P= 0.0115<br>Lviv: from 6.4% to 2.4% P = 0.0015<br>• No significant change in NICU admission in one maternity:<br>Donetsk: from 11.2% to 9.4%<br>• Significant decrease in the proportion of hypothermic infants:<br>Donetsk: from 59.5% to 0.6% P<0.0001<br>Lutsk: from 85.8% to 0.3% P<0.0001<br>Lviv: from 77% to 0.7% P<0.0001

**Author**	**Study design / Year study conducted/ Level of significance / Adjustments**	**Country / Setting**	**Population / Number of deliveries**	**Intervention strategy / Frequency and duration**	**Effect of intervention** (Kirkpatrick levels 3 or 4)
Cottrell 2021	Cohort study<br><br>2013-2017<br><br>P< 0.05<br><br>No adjustments	USA<br><br>1 Teaching hospital	Women with prolonged second stage labour (N=2720 at baseline, N= 2800 intervention period Total N=5520)	On-the-job, hands-on AVB training of residents and supervision by attending physicians.<br><br>Frequency and duration not stated.	• Significant increase in forceps deliveries, from 1.8% to 4.0%, P < 0.001<br>• Significant decrease in vacuum deliveries, from 8.7% to 6.5%, P = 0.002.<br>• No significant change in overall AVB, from 10.5% to 10.5%<br>• Increase in overall CS rate from 30.4% to 32.1%, no statistical analysis<br>• 3rd degree laceration: no change in forceps (post vs. pre, RR 0.5, 95% CI 0.24-1.21) and vacuum groups (post vs. pre, RR 0.8, 95% CI 0.37−1.55)<br>• 4th degree laceration: no change in vacuum (post vs. pre, RR 1.0, 95% CI 0.28-3.83); significant decrease in forceps group (post vs. pre, RR 0.1, 95% CI 0.01-0.73)<br>• Overall maternal complication rates: significant decrease in forceps group (post vs. pre, RR 0.40, 95% CI 0.19-0.75); no change in vacuum group (post vs. pre, RR 0.8, 95% CI 0.44-1.51)<br>• Facial laceration: no change in forceps (post vs. pre, RR 0.9, 95% CI 0.08-9.42) and vacuum groups (post vs. pre, RR 0.8, 95% CI 0.19-3.23)<br>• Scalp injury: no change in forceps (post vs. pre, Not estimable) and vacuum groups (post vs. pre, RR 0.5, 95% CI 0.15-1.46)<br>• Cephalohematoma: no change in forceps (post vs. pre, Not estimable) and vacuum groups (post vs. pre, RR 0.8, 95% CI 0.22-2.51)<br>• Overall neonatal complications: no change in forceps (post vs. pre, RR 0.4, 95% CI 0.09-2.09), vacuum groups (RR 0.6, 95% CI 0.31−1.25) and overall (post vs. pre, RR 0.4, 95% CI 0.09-2.09)<br>• No significant change in AVB failure rate (requiring CS) (1.8% requiring CS before the initiative and 1.4% after the initiative)

**Author**	**Study design / Year study conducted/ Level of significance / Adjustments**	**Country / Setting**	**Population / Number of deliveries**	**Intervention strategy / Frequency and duration**	**Effect of intervention** (Kirkpatrick levels 3 or 4)
Dmello 2021	Before and after intervention study<br><br>2011-2019<br><br>P <0.05<br><br>No adjustments	Tanzania<br>22 health facilities	All women<br>(N= 74,792 at baseline, N=666,490 during intervention period-continuous)<br>Total N=741,282	Complex, dynamic, multicomponent, multifaceted intervention to improve the quality of care and survival during pregnancy and childbirth.<br>Components of AVB training: theoretical, simulation, short on-site hands-on training and prolonged on-site supervision (in some settings), provision of VE, quarterly site visits to support on-the-job coaching. At large high-volume health facilities, an on-site mentor was stationed.VE rate was systematically targeted and monitored every month, reported quarterly and annually.<br>**Frequency and duration:** 5-day critical BEmONC skills course; 1-day modular course on specific topics addressing gaps detected in perinatal audits; on-site trainer: an experienced nurse or doctor supported by CCBRT was periodically stationed full time for periods from 2 weeks to 1 year in the labour ward of high-volume facilities for on-site coaching and support; and on-the-job coaching (mentors would spend 2–3 days in each of the 22 sites, building skills during routine service provision).	• Significant increase in VE from 0.03% in 2011 to 3.1% in 2019 (RR 1.43; 95% CI 1.41 to 1.43)<br>• Significant increase in overall CS rate from 2.3% in 2011 to 17.6% in 2019 (RR 1.15; 95% CI 1.14 to 1.15)<br>• Significant decrease in MMR: from 154 to 79 per 100 000 live birth (RR 0.92, 95% CI 0.9 to 0.95)<br>• Significant decrease in stillbirth rate from 26 to 21 per 1000 live births (RR 0.96, 95% CI 0.96 to 0.97)<br>• Significant increase in neonatal deaths (sum of deaths among inborn and referred babies) from 5.5 in 2011 to a peak of 14.5) in 2014 and then slightly declined to 12.1 in 2019 (RR 1.05, 95% CI 1.04 to 1.05)

**Author**	**Study design / Year study conducted/ Level of significance / Adjustments**	**Country / Setting**	**Population / Number of deliveries**	**Intervention strategy / Frequency and duration**	**Effect of intervention** (Kirkpatrick levels 3 or 4)
Dominico 2018	Before and after intervention study (ITS)<br><br>2011-2016<br><br>No statistical analyses<br><br>No adjustments	Tanzania<br><br>5 Tertiary hospitals<br>10 Primary health centres	All women<br>(N=2446 at baseline, N=125,908 intervention period<br>Total N=128,354)	Multifaceted intervention comprising decentralization of life-saving services; task-shifting obstetric procedures to other cadres; training that included skill building in vacuum extraction.<br><br>Frequency and duration not stated.	• Increase in vacuum extractions from 0.15% at baseline (no statistical analyses):<br>1st year after intervention: 0.88%<br>2nd year after intervention: 2.11%<br>3rd year after intervention: 1.54%<br>4th year after intervention: 1.26%<br>5th year after intervention: 1.73%<br>• Decrease in CS rates from 11.25% at baseline (no statistical analyses):<br>1st year after intervention: 10.85%<br>2nd year after intervention: 9.42%<br>3rd year after intervention: 9.88%<br>4th year after intervention: 9.80%<br>5th year after intervention: 11.18%
Dumont 2013	Cluster-randomised trial<br><br>2007-2011<br><br>P< 0.05<br><br>OR adjusted for country, hospital type and characteristics, patient characteristics, and birthweight (for perinatal outcomes).	Mali and Senegal<br><br>Referral hospitals (23 in intervention and 23 in control)	All women<br>(N=84,924 at baseline, N=106,243 intervention period<br>Total N=191,167 deliveries)	Multifaceted intervention focused on reducing MM and improving quality of obstetric care comprising onsite training in emergency obstetric care (using Advances in Labour and Risk Management (ALARM course) and update/creation of clinical guidelines, and audit and feedback on maternal deaths,<br><br>Frequency and duration: 6 days once a year (for 2 years)	• Significant increase in overall AVB rate in Senegal from 1.24% to 2.24% in intervention groups (adjusted OR 3.10, 95% CI 1.85 to 5.20)<br>• No significant change in overall AVB rate in Mali from 3.32% to 1.97% in intervention groups, (adjusted OR 0.51, 95% CI 0.16 to 1.59)<br>• Significant decrease in intrapartum CS (adjusted OR 0.87, 95% CI 0.82 to 0.92)<br>• Significant increase in emergency antepartum CS (OR 1.33, 95% CI 1.19 to 1·50), mainly due to pre-eclampsia/eclampsia<br>• Significant decrease in hospital maternal mortality (OR 0.85, 95% CI 0.73 to 0.98)<br>• Marginally significant increase in transfusions (adjusted OR 1.44, 95% CI 0.99 to 2.11)<br>• Significant decrease in neonatal mortality < 24 h (adjusted OR 0.74, 95% CI 0.61 to 0.90)<br>• No significant change in stillbirth rates (OR 1.05, 95% CI 0.91 to 1.22)

**Author**	**Study design / Year study conducted / Level of significance / Adjustments**	**Country / Setting**	**Population / Number of deliveries**	**Intervention strategy / Frequency and duration**	**Effect of intervention** (Kirkpatrick levels 3 or 4)
Geelhoed 2018	Qualitative implementation study with presentation of before and after routine health service data.<br><br>2015-2017<br><br>No statistical analyses<br><br>No adjustments	Mozambique<br><br>Tetu Province Hospitals and primary health facilities with > 100 births per 3-months-period; this amounted to 52-82 out of a total of some 120 health facilities	All women<br>(N= 88,000 at baseline, N=265,061 intervention period<br>Total N = 353,061 births)	Multifaceted intervention (training on the management of prolonged labour including vacuum extraction; accreditation in emergency obstetric care; monitoring and evaluation of routine data from all maternities; audit and feedback of CS.<br>Clinicians at the provincial hospital were assigned to provide in-service training to any MCH nurse or doctor wishing to strengthen their capabilities.<br>Key components of AVD module:<br>1) didactic training<br>2) simulation training<br>3) Hands-on training<br>4) Audit & feedack<br><br>Duration:<br>VE training: 1 week<br>QI strategy: 3 years	• Increase in vacuum deliveries from 0.2% at baseline (no statistical analyses):<br>1st yr after intervention: 1.0%<br>2nd yr after intervention: 2.3%<br>3rd yr after intervention: 2.2%<br>• Decrease in overall CS rate from baseline rate of 2.7% (no statistical analyses):<br>1st yr after intervention: 2.1%<br>2nd yr after intervention: 2.1%<br>3rd yr after intervention: 1.8%<br><br>• Decrease in institutional maternal mortality rate of 126/100,000 births at baseline (no statistical analyses):<br>1st year after intervention: 49/100,000<br>2nd year after intervention:48/100,000<br>3rd year after intervention: 44/100,000<br><br>• Decrease in institutional stillbirth rate from baseline of 17.7/1000 newborns (no statistical analyses):<br>1st year: 14.9/1000<br>2nd year: 12.4/1000<br>3rd year: 12.2/1000
Gulmezoglu 2006	Cluster-randomised trial<br><br>2001-2002<br><br>P <0.05<br><br>No multivariable regressions. End rates adjusted by baseline rates	Mexico, Thailand<br><br>**Mexico:** 22 health facilities not associated directly with a university or other academic or research department<br><br>**Thailand:** 18 primary and secondary health facilities	**Mexico:** All women (N=19,592 at baseline, N=22,236 intervention Total N=41,828)<br><br>**Thailand:** All women (N=17,655 at baseline, N=17,998 intervention Total N=35,653)	Multifaceted educational intervention to promote the use of the WHO Reproductive Health Library<br><br>Frequency and duration: Three 1-day workshops over a period of 6 months. Other components were continuous over the time of the trial	**Mexico:** No significant changes in intervention or control groups from baseline to adjusted end-of-study VE rates (intervention: 0.6% to 0.2% and control: 0.4% to 0.1%). Difference in adjusted end-of-study VE rate: 0.1, 95% CI -0.1 to 0.2<br><br>**Thailand:** No significant changes in intervention or control groups from baseline to adjusted end-of-study VE rates (intervention: 7.5% to 7.7% and control: 6.3% to 7.7%). Difference in adjusted end-of-study VE rate: 0.0, 95% CI -1.5 to 1.4

**Author**	**Study design / Year study conducted / Level of significance / Adjustments**	**Country / Setting**	**Population / Number of deliveries**	**Intervention strategy / Frequency and duration**	**Effect of intervention** (Kirkpatrick levels 3 or 4)
Mogilevkina 2022	Non-randomized controlled trial<br><br>2004-2009<br>Baseline: 2004-2005<br><br>Intervention: 2006-2007 (not analyzed)<br><br>After intervention: 2008-2009<br><br>P<0.05<br><br>No adjustments	Ukraine<br>Intervention: 28 city or district hospitals<br><br>Control: 36 city or district hospitals	All women<br><br>Intervention hospitals:<br>2004-2005: 47,838<br>2008-2009: 61,116<br><br>Control hospitals:<br>2004-2005: 38,032<br>2008-2009: 42,866	Multifaceted EMOC intervention using the Advances in Labour and Risk Management (ALARM) International Program (AIP). One 5-day course focused on the five main causes of maternal mortality. AVB training is one of the sessions.	• Significant increase in VE from 0.10% to 0.69% in intervention group (OR: 2.86; 95% CI: 1.80-4.57, P<0.001)*<br>• Significant decrease in Forceps rates from 0.17% to 0.08% in intervention group (OR: 1.80; 95% CI: 1.00-3.25, P= 0.041)**<br>• Significant increase in overall CS rate from 13.12% to 15.93% in intervention group (OR: 1.11; 95%CI: 1.06-1.17, P<0.001)*<br>• MM: non-significant change<br>• PPH > 1000 ml: non-significant change<br>• Post-partum hysterectomy: non-significant change<br>• Blood transfusion: significant decrease in intervention group from 2.11% to 0.48% (OR: 0.56; 95% CI: 0.48-0.65, P<0.001)*<br>• Plasma transfusions: significant decrease in intervention group from 4.56% to 2.28% (OR: 0.70; 95% CI: 0.63-0.78, P<0.001)*<br>• Uterus explorations: significant decrease in intervention group from 8.32% to 4.14% (OR: 0.64; 95% CI: 0.59-0.69, P<0.001)*<br><br>*Difference in difference estimator comparing the change in the rate of events or outcomes in the control versus the intervention group<br>**The decrease in forceps rate was larger in the control than in the intervention group.

**Author**	**Study design / Year study conducted/ Level of significance / Adjustments**	**Country / Setting**	**Population / Number of deliveries**	**Intervention strategy / Frequency and duration**	**Effect of intervention** (Kirkpatrick levels 3 or 4)
Nolens 2016	Before and after intervention study<br><br>2012-2014<br><br>P<0.05<br><br>No adjustments	Uganda<br><br>1 Teaching hospital	Women with "medium to high risk pregnancies" (N=12,143 at baseline N= 34,894 intervention period<br>Total N=47,037)	Development of a vacuum extraction guideline, supply of vacuum extraction equipment, training of staff in didactic and simulation sessions, and on the job supervision (for 1 week, after formal training sessions) and continued continuous supervision on labour ward for 4 months during which most residents had several labour ward shift<br><br>Frequency and duration: 4 months initial training accompanied by yearly refresher (theory and simulation training session)	• Significant increase in vacuum deliveries from 0.6% to 2.1%, P<0.01<br>• Significant increase in overall CS rate from 28.2% to 30.2%, P< 0.01<br>• No significant change in CS due to obstructed labour from 6.0% to 6.0%<br>• Significant decrease in women with ruptured uterus from 1.1% to 0.8%, P< 0.01<br>• No significant change in overall maternal deaths from 0.8% to 0.7%<br>• No significant change in maternal deaths due to intrapartum complications from 0.3% to 0.2%<br>• No significant change in maternal ICU admission from 1.9% to 1.8%<br>• Significant decrease in total perinatal deaths (stillbirths + neonatal deaths during admission) from 9.2% to 8.4%, P=0.02<br>• Significant decrease in intrapartum stillbirths from 3.4% to 2.6% , P< 0.01<br>• Significant increase in term admissions to NICU from 8.7% to 10.0%, , P< 0.01<br>• No significant change in term neonatal deaths from 1.3% to 1.4%
Skinner 2017	Retrospective cohort study (ITS)<br><br>2005-2014<br><br>P<0.05<br><br>No adjustments	Australia<br><br>1 Teaching hospital	All women excluding those with preterm or anomalous births, or who had CS without prior instrumental attempt (Total N=72,490 births)	Residency training program: formalized lecture series and mannequin simulation instrumental birth training and; mandatory instrumental birth credentialing for all obstetric residents	• Significant increase in forceps births from 4.8% last year pre-intervention to 9.8%-last year post-intervention (average annual rate (β) 1.5 per 100 births, 95% CI 1.03 to 1.96 per 100 births)<br>• Significant decrease in vacuum births from 8.0% last year pre-intervention to 6.0% last year post-intervention (β -1.43, 95% CI -2.5 to -0.37)<br>• Significant decrease in postpartum haemorrhage (β -1.3, 95% CI -2.07 to -0.49)<br>• No significant change in 3rd-4th degree tears (β -1.04, 95% CI -3.1 to 1.00)<br>• No significant change in composite neonatal morbiditya (β -0.18, 95% CI -0.38 to 0.02)

**Author**	**Study design / Year study conducted/ Level of significance / Adjustments**	**Country / Setting**	**Population / Number of deliveries**	**Intervention strategy / Frequency and duration**	• **Effect of intervention** (Kirkpatrick levels 3 or 4)
Solt 2011	Retrospective cohort (pre-post intervention)<br><br>Year study conducted not stated<br><br>P<0.05<br><br>No adjustments	USA<br><br>1 Teaching hospital	All women (N=3481 at baseline N=4338 intervention period Total N=7819)	Day time coverage of labour and delivery by a specially recruited senior generalist obstetrician with 35 years' clinical experience<br><br>Duration: 2 years	• Significant increase in forceps deliveries, from 4.9% to 7.8%, P < 0001<br>• Significant decrease in vacuum deliveries, from 6.4% to 2.9%, P < 0001<br>• No significant change in Overall AVB: from 11.3% to 10.6%<br>• No significant change in Overall CS, from 25.5% to 27.3%<br>• No significant change in<br>-3rd-4th degree tears, from 3.6% to 3.1%<br>-5 min Apgar < 7, from 1.9% to 2.4%<br>-Birth injuries (fracture, laceration, palsy), from 0.2% to 0.3%
Sorensen 2010	Prospective intervention study (before and after training evaluation)<br><br>2008<br><br>P<0.05<br><br>No adjustments	Tanzania<br><br>1 Teaching hospital	All women giving birth to a neonate ≥ 1000 g (N= 558 at baseline N= 550 intervention period Total N=1108)	2-day Advanced Life Support in Obstetrics (ALSO) provider course comprising lectures, workshops, and case discussions on the major topics of emergency obstetric and neonatal care (EmONC). In the workstation on vacuum delivery, hands-on skills were practiced using pelvic and fetal mannequins and the Kiwi OmniCup	• No change in vacuum delivery rates (0% to 0%)<br>• No significant change in CS before active labor, from 2.0% to 1.8%<br>• No significant change in emergency CS, from 6.9% to 6.3%<br>• No significant change in CS for prolonged labor, from 5.1% to 4.4%<br>• Significant decrease in death of liveborn with Apgar ≥ 4, from 1.1% to 0%, P=0.03<br>• No significant change in:<br>-Neonatal deaths, from 1.2% to 0.4%<br>-Stillbirths, from 1.7% to 2.5%<br>-Neonatal resuscitation, from 3.0% to 2.4%<br>• -1-min Apgar score < 7 from 6.4% to 6.9%

Author	Study design / Year study conducted/ Level of significance / Adjustments	Country / Setting	Population / Number of deliveries	Intervention strategy / Frequency and duration	Effect of intervention (Kirkpatrick levels 3 or 4)
Takeda 2018	Before and after study, ITS<br><br>2016<br><br>No statistical analyses<br>No adjustments	Japan<br><br>1 Primary health facility	All women<br>(N=4607 at baseline,<br>N= 650 intervention period<br>Total N=5257)	Forceps delivery simulation training comprising lectures with forceps delivery videos, demonstrations, and supervised simulation forceps deliveries	• Forceps rate increased from baseline 0%-2.0% in the 10 baseline pre-intervention years to 4.3% in the post-intervention year (no statistical analyses)<br>• Vacuum rates decreased from 1.2-13.3% in the 10 baseline pre-intervention years to 2.3% in post-intervention year (no statistical analyses)<br>• Overall CS rates decreased from 7.3%-25.6% in the 10 baseline pre-intervention years to 22.2% in post-intervention year (no statistical analyses)

AVB: assisted vaginal birth. CI: confidence interval. EmONC: emergency obstetric and neonatal care. ITS: interrupted times series. NICU: neonatal intensive care unit. OR: odds ratio. RR: relative risk. SD: standard deviation

a. Composite neonatal morbidity: a composite of low Apgar at 5 min (<7), neonatal intensive care unit or special care nursery admission, cephalohematoma, lacerations or abrasions, bruising, intraventricular hemorrhage, brachial plexus injury, fractured clavicle, convulsions, phototherapy, neonatal feeding problems, and respiratory distress syndrome.
