# Supplementary material for: Interventions to reintroduce or increase assisted vaginal births: a systematic review of the literature
Source: BMJ Open. 2023 Feb 14;13(2):e070640. doi: 10.1136/bmjopen-2022-070640 (PMC9930566; doi:10.1136/bmjopen-2022-070640)
Supplement: Supplementary data [file bmjopen-2022-070640supp004.pdf]

Supplementary file 4. Assessment of the quality of 16 included studies

Risk of bias of 13 non-randomized intervention studies[a]

Study	Bias due to confounding	Bias in selection of participants into the study	Bias in classification of interventions	Bias due to deviations from intended interventions	Bias due to missing data	Bias in measurement of outcomes	Bias in selection of the result	Overall bias [b]
Bardos 2017	Low	Low	Low	Low	Low	Low	Low	Low
Becker 2020	Moderate	Low	Low	Low	Low	Low	Low	Moderate
Berglund 2010	Moderate	Low	Low	Low	Low	Low	Low	Moderate
Cottrell 2021	Serious	Serious	Low	Low	Low	Low	Low	Serious
Dmello 2021	Moderate	Low	Low	Low	Low	Low	Low	Moderate
Dominico 2018	Serious	Low	Low	Low	Low	Low	Low	Serious
Geelhoed 2018	Serious	Low	Low	Low	Low	Low	Low	Serious
Mogilevkina 2022	Moderate	Low	Low	Low	Low	Low	Low	Moderate
Nolens 2016	Serious	Low	Low	Low	Low	Low	Low	Moderate
Skinner 2017	Low	Moderate	Low	Low	Low	Low	Low	Moderate
Solt 2011	Moderate	Low	Low	Low	Low	Low	Low	Moderate
Sorensen 2010	Moderate	Low	Low	Low	Low	Low	Low	Moderate
Takeda 2018 [c]	-	-	-	-	-	-	-	-

[a] Based on Risk of Bias for Non-Randomised Studies of Interventions (ROBINS-I) tool.
[b] Interpretation of overall risk of bias.
[c] Takeda 2018:Risk of bias not assessed due to insufficient information.

Low risk of bias: The study is comparable to a well performed randomised trial.
Moderate risk of bias: The study provides sound evidence for a nonrandomized study but cannot be considered comparable to a well performed randomised trial.
Serious risk of bias: The study has some important problems.
Critical risk of bias: The study is too problematic to provide any useful evidence and should not be included in any synthesis.
No information: No information on which to base a judgement about risk of bias.

Risk of bias of 3 randomized trials[a]

Study	Random sequence generation (selection bias)	Allocation concealment (selection bias)	Baseline outcome measurements	Baseline characteristics	Blinding of participants and personnel (performance bias)	Blinding of outcome assessment (detection bias)	Contamination	Incomplete outcome data (attrition bias)	Selective reporting (reporting bias)	Overall bias[b]
Ameh 2014	Low	Low	Low	Low	Low	Low	Low	Low	Low	Low
Dumont 2013	Low	Low	Low	Low	Low	Low	Low	Low	Low	Low
Gulmezoglu 2006	Low	Low	Low	Low	Low	Low	Low	Low	Low	Low

[a] Based on the Cochrane EPOC Risk of bias criteria for randomised trials.
[b] Interpretation of overall risk of bias

Low risk of bias: Low risk of bias for key quality domains (i.e. allocation sequence generation and concealment)
High risk of bias: High risk of bias for one or more of the key domains
Unclear risk of bias: Unclear risk of bias for one or more key domains
