# Supplementary material for: Interventions to reintroduce or increase assisted vaginal births: a systematic review of the literature
Source: BMJ Open. 2023 Feb 14;13(2):e070640. doi: 10.1136/bmjopen-2022-070640 (PMC9930566; doi:10.1136/bmjopen-2022-070640)
Supplement: Supplementary data [file bmjopen-2022-070640supp005.pdf]

Supplementary file 5. Baseline rates of assisted vaginal births and cesarean deliveries in studies included in the systematic review

Baseline rates[a]	N of studies	References
VE rates (N=14 studies, 15 data sets[b])		
range	0% to 8.7%	
< 1%	8	Ameh 2014 (Kenya), Dmello 2021 (Tanzania), Dominico 2018 (Tanzania), Geelhoed 2018 (Mozambique), Gulmezoglu 2006 (Mexico[c]), Mogilevkina 2022 (Ukraine), Nolens 2016 (Uganda), Sorensen 2010 (Tanzania)
1- 4 %	3	Takeda 2018 (Japan), Becker 2020 (USA), Bardos 2017 (USA)
> 4%	4	Solt 2011 (USA), Gulmezoglu 2006 (Thailand[c]), Skinner 2017 (Australia), Cottrell 2021 (USA)
Forceps rates (N=7 studies)		
range	0% to 5.2%	
< 1%	3	Bardos 2017 (USA), Mogilevkina 2022 (Ukraine), Takeda 2018 (Japan)
1-3%	2	Becker 2020 (USA), Cottrell 2021 (USA)
>3%	2	Skinner 2017 (Australia), Solt 2011 (USA)
Overall AVB rates (N=5 studies, 6 data sets[d])		
range	1.8% to 11.3%	
1-3%	3	Dumont 2013 (Senegal and Mali[e]) , Berglund 2010[f] (Ukraine)
>3 to 4.9%	1	Becker 2020 (USA)
5 to 10%	0	
>10%	2	Cottrell 2021 (USA), Solt 2011 (USA)
Overall CS rates (N=12 studies)		
range	2.6% to 30.6%	
< 10 %	3	Dmello 2021 (Tanzania), Geelhoed 2018 (Mozambique), Mogilevkina 2022[g] (Ukraine)
11-20%	1	Dominico 2018 (Tanzania)
21-30%	6	Ameh 2014 (Kenya), Solt 2011(USA), Takeda 2018 (Japan), Bardos 2017 (USA), Nolens 2016 (Uganda), Berglund 2010[f] (Ukraine)
>30%	2	Cottrell 2021 (USA), Becker 2020 (USA)

[a.] In studies with >1 time point, we present the lowest AVB rate and the highest CS rate before intervention. [b] One study (Gulmezoglu 2006) conducted in two countries (Mexico & Thailand) analyzed separately. [c] Gulmezoglu 2006 (randomized trial): rates in control groups. [d] One study (Dumont 2013) conducted in two countries (Senegal & Mali). [e] Dumont 2013 (cluster randomized trial): rates in control groups. [f] Berglund 2010: combined rates from three sites. [g] Mogilevkina 2022 (non-randomized trial): rate in control group.
AVB: Assisted vaginal birth; CS: Cesarean section; VE: Vacuum extraction
