# Supplementary material for: Interventions to reintroduce or increase assisted vaginal births: a systematic review of the literature
Source: BMJ Open. 2023 Feb 14;13(2):e070640. doi: 10.1136/bmjopen-2022-070640 (PMC9930566; doi:10.1136/bmjopen-2022-070640)
Supplement: Supplementary data [file bmjopen-2022-070640supp006.pdf]

Supplementary file 6. Details of interventions in 16 included studies

Study	Details of intervention strategy	Health provider included in intervention strategy / Number (N)	Mode of delivery of intervention strategy	Frequency and duration of intervention strategy	AVB instrument used in strategy
Ameh 2014	Multifaceted intervention:<br>1. **EmONC training**<br>• Training using adapted version of the Life Saving Skills-Emergency Obstetric and Newborn Care (LSS-EONC) training package, a competency-based training developed specifically for low resource settings. The course covers a list of 10 topics, including AVB. A cross cutting theme throughout the training is effective communication and teamwork. In the adaptation, forceps training was excluded and only VE was taught for AVB.<br>• **Multidisciplinary training approach:** midwives, medical doctors and clinical officers are trained together by multidisciplinary teams of experienced specialist obstetricians, midwives and anaesthetists (UK based volunteers and in-country volunteers).<br>• **On-site training:** the training is delivered as close as possible to the health care providers' place of service delivery.	Obstetricians, medical doctors, clinical officers, midwives<br><br>Overall, 400 maternity care workers were trained.<br><br>In each of the 10 hospitals, 72-100% of relevant staff were trained.	Face-to-face interactive learning sessions comprising of short lectures, simulation, and hands-on sessions using low fidelity obstetric, newborn and resuscitation manikins	3 days	Vacuum

	**Details of intervention strategy**				
Ameh 2014, continued	2. **Local supportive supervision:** local master trainers were trained (1-day "train the trainer" course) and mentored during another EmOC training course - covering the principles of supportive supervision, tools for supportive supervision, developing and implementing action plans from the process of supportive supervision. These local master trainers (4-6 per hospital) were usually obstetricians, senior midwives, clinical based on their knowledge and potential to teach/mentor others on the job. Local supportive supervisors were expected to provide on the job support to other colleagues, facilitating behaviour change after the training.<br><br>3. **Essential equipment:** all hospitals were provided with essential equipment including 4 reusable Kiwi omnicups per hospital.				

Study	Details of intervention strategy	Health provider included in intervention strategy / Number (N)	Mode of delivery of intervention strategy	Frequency and duration of intervention strategy	AVB instrument used in strategy
Bardos 2017	Senior obstetricians (more than 20 years experience) teaching and supervising of residents on forceps deliveries.<br>• One to two senior obstetricians covered labor and delivery at least 5 days a week.<br>• Two senior obstetricians supervised resident deliveries during daytime (7:00 am to 7:00 pm); one supervised resident deliveries during nights and weekends (with a second obstetrician available as backup in the event of an emergency or increased volume).	Residents<br><br>N not stated	On-site, face-to-face teaching and supervision of forceps deliveries	Not stated	Forceps
Becker 2020	Assisted vaginal birth (AVB) educational curriculum consisting of didactics and simulation. The curriculum comprised twice-yearly workshops for residents (excluding first year residents). A typical workshop would have 10 to 12 participants.<br><br>Components of the workshop<br>• 28-question pretest to evaluate basic AVB knowledge; 1-hour didactic on the history, indications, safe use, and risks of forceps and vacuums; and<br>• 1-hour hands-on simulation where residents are taught how to properly execute an AVB (forceps and vacuum) using a standard maternal pelvis model and dummy fetus.<br><br>The didactic portion of the workshop is delivered to the entire group of residents. The hands-on simulation is performed in groups of three to four residents paired with one to two faculty members. After the session, residents complete a 50-question posttest, with a passing mark of 70%.	Residents<br><br>N not stated	Face-to-face lectures, hands-on simulation using a standard maternal pelvis model and dummy fetus	One 2-hour workshop offered every 6 months<br><br>Duration of hands-on training / supervision not stated	Forceps, Vacuum

Study	Details of intervention strategy	Health provider included in intervention strategy / Number (N)	Mode of delivery of intervention strategy	Frequency and duration of intervention strategy	AVB instrument used in strategy
Berglund 2010	Multifaceted intervention comprising:<br>• Continuous monitoring and evaluation system with feedback to the staff, improving medical services and counseling with special attention to resuscitation, breast feeding, and prevention of infections and to strengthen continuity of care.<br>• Development and dissemination of national, evidence-based obstetrical and neonatal protocols.<br>• Staff training on evidence-based guideline. The Russian version of the training material 'Effective Perinatal Care' (EPC), developed by WHO Regional Office for Europe was used. The material is a comprehensive, didactic, interactive two-week training package including clinical activities and a team approach to maternity care<br>• Revision of university curricula to include evidence-based perinatal care.<br>• Participating maternities were provided with basic equipment for resuscitation and neonatal care and tools for the delivery room to encourage change of position during labor and more family-oriented care.<br><br>After evaluation of the progress in the maternities by trainers, additional practical training was provided: reinforcement training in neonatal care (nine days), reinforcement in modern perinatal technology (nine days) and care in second stage in preterm delivery (one day).	Obstetricians, neonatologists, midwives, pediatric nurses, pediatricians, anesthesiologists	Face-to-face, practical clinical scenarios,	Initial Effective Perinatal Care (EPC) training: 2 weeks<br><br>Follow up EPC training: 3 days	Vacuum

Study	Details of intervention strategy	Health provider included in intervention strategy / Number (N)	Mode of delivery of intervention strategy	Frequency and duration of intervention strategy	AVB instrument used in strategy
Cottrell 2021	On-the-job, hands-on AVB training of residents and supervision by attending physicians.	Residents<br><br>N not stated	Hands-on training	Not stated	Forceps, Vacuum
Dmello 2021	Multiple intervention components addressed gaps across the maternal and perinatal continuum of care (training, infrastructure, routine data quality strengthening and utilization). Multiple interventions were implemented to improve the quality of Basic Emergency Obstetric Care, towards addressing maternal and perinatal mortality; specifically targetted were the EMOC signal functions that include vacuum extraction.<br><br>Components:<br>• 2-week BEmONC national training, facilitated by national trainers;<br>• 5-day critical BEmONC skills course;<br>• 1-day modular course on specific topics addressing gaps detected in perinatal audits;<br>• on-site trainer: an experienced nurse or doctor supported by CCBRT was periodically stationed full time for periods from 2 weeks to 1 year in the labour ward of high-volume facilities for on-site coaching and support;<br>• on-the-job coaching (mentors would spend 2–3 days in each of the 22 sites, building skills during routine service provision).<br>Provision of vacuum extractors was a critical component of the intervention.	Doctors and midwives.<br><br>N=1540	Face to face, videos, simulation training, group discussions, hands-on coaching	Duration of each intervention component: in 2nd column<br><br>Frequency: NI	Vacuum

Study	Details of intervention strategy	Health provider included in intervention strategy / Number (N)	Mode of delivery of intervention strategy	Frequency and duration of intervention strategy	AVB instrument used in strategy
Dominico 2018	Strategy for the 10 primary health care centers<br>• Decentralization of life-saving services from district or regional hospitals to primary health care centres. They were upgraded to provide comprehensive emergency obstetric and newborn care (C-EmONC) (i.e. they were staffed and equipped to provide obstetric surgery, blood transfusion, and basis EmONC signal functions).<br>• Task-shifting obstetric procedures from doctors, to advanced level associate clinicians or assistant medical officers (AMOs), and anaesthesia to associate clinicians or nurse anaesthetists and clinical officers.<br>Strategy for the 5 hospitals<br>• Three months clinical training (that included skill building in vacuum extraction (VE) at the two largest medical training centres in the country.<br>• Training was followed by week-long Continuing Medical Education (CME) sessions that focused on specific topics that were identified as requiring further confidence- and skills-building, such as VE; training on mannequins.<br>• Training in VE also included how to manage complications such as postpartum haemorrhage and perineal lacerations.<br>• Human resources support, clinical audit, on-going mentoring supervision.<br>• On-the-job coaching and hands-on mentoring by experts during monthly on-site visits.<br>For all institutions:<br>• Weekly conferences allowing staff to discuss specific cases and networking with others to	Assistant medical officers, nurse-midwives<br><br>N = 80	Groups of 9-14 people (face-to-face). Training was competency-based<br><br>Trainees used anatomical models for practice. They would observe procedures being performed but had only few opportunities to perform a VE on a patient<br><br>eLearning sessions were developed.<br>In the case of VE, the trainers adopted WHO's video on VE and translated it into Swahili	Not stated	Vacuum

	**Details of intervention strategy**				
Dominico 2018, continued	For all institutions:<br>• Weekly conferences allowing staff to discuss specific cases and networking with others to facilitate specialist to be "on-call" so emergency consultations were possible day and night.<br>• A WhatApp group was created.<br>• Audit and feedback about procedures and outcomes				

Study	Details of intervention strategy	Health provider included in intervention strategy / Number (N)	Mode of delivery of intervention strategy	Frequency and duration of intervention strategy	AVB instrument used in strategy
Dumont 2013	Multicomponent intervention including:<br><br>6-day training workshop provided by certified instructors in each hospital to one doctor and one midwife (train the trainer) who were responsible for maternity services.<br>• Training used ALARM (Advances in Labour and Risk Management) international course.<br>• The session consisted of 3 days of training in best practices in emergency obstetric care, 1 day of training in maternal death reviews, 1 day of awareness training related to economic, socio cultural, and ethical barriers (including sexual and reproductive rights), and 1 day of training in adult education methods.<br>• At the end of the session, a normative evaluation was done.<br>• The trainers had to attend two recertification sessions (once a year) to verify their knowledge, update them on the clinical content and process of maternal death audits, discuss their roles, share their experiences, and confirm their capacity to provide leadership in their clinical settings.<br><br>Audit and feedback on maternal deaths.<br>• A multidisciplinary audit committee including physicians, midwives, nurses, administrators was created in each participating site and trained in the process of undertaking maternal death reviews.	Doctors and midwives<br><br>N = 46	Face-to-face	6 days once a year (for 2 years)	Forceps, Vacuum

	**Details of intervention strategy**				
Dumont 2013, continued	Audit and feedback on maternal deaths (continued)<br>• The audit cycle and onsite training were then launched in each intervention site with the support of external facilitators (certified instructors) during their quarterly educational outreach visits.<br><br>If needed, local trainers who took part in the initial training workshop developed **new clinical guidelines** or updated existing guidelines according to best practices for emergency obstetric care.				

Study	Details of intervention strategy	Health provider included in intervention strategy / Number (N)	Mode of delivery of intervention strategy	Frequency and duration of intervention strategy	AVB instrument used in strategy
Geelhoed 2018	Four complementary interventions:<br><br>1. One-week training on the management of prolonged labour, including vacuum extraction and craniotomy, developed and facilitated by a professor considered an expert on obstetric care in low-income countries both internationally and in Mozambique. Clinicians at the provincial hospital were assigned to provide in-service training to any MCH nurse or doctor wishing to strengthen their capabilities<br><br>2. Strengthening of the quarterly emergency obstetric care accreditation process<br><br>3. Monthly audit of clinical files of all cases of caesarean section in the provincial hospital (a large majority of all caesarean sections in the province) with specific feedback to clinicians involved, copied to relevant provincial managers<br><br>4. Strict monitoring and evaluation of routine data from all maternities, with specific feedback to district health directors and doctors, copied to all and to relevant provincial manager<br><br>Sharing of capabilities between staff was encouraged. Prizes and public recognition of successful hospitals.	Provincial level: nurse-midwives, general doctors, one consultant OB-GYN specialist<br><br>District level: nurse-midwives<br><br>335,971 births (2014 to 2017)	Face-to-face (1-week training including VE)	1-week training including VE (provided only once at provincial level with a consultant OB-GYN / facilitator, and repeated in a simplified and shortened format in most of the districts in the province in the following months)<br><br>QI strategy: 3 years	Vacuum

Study	Details of intervention strategy	Health provider included in intervention strategy / Number (N)	Mode of delivery of intervention strategy	Frequency and duration of intervention strategy	AVB instrument used in strategy
Gulmezoglu 2006	Multifaceted intervention addressing perceived barriers to evidence-based practice.<br>• Meeting with hospital directors/heads of obstetrics and gynaecology departments before intervention for their consent and support (to ensure organisational buy in).<br>• Provision of the WHO Reproductive Health Library (RHL), computers and printer in an accessible part of the labour ward/department (to ensure easy access to the RHL).<br>• Identification of a hospital RHL coordinator from staff (to help health care workers to use RHL and to maintain links with the research team in case of problems).<br>• RHL information materials (poster, short printouts) to promote awareness in health care workers.<br>• A series of interactive workshop delivered by a specialist (to teach principles of evidence-based practice and use of RHL). Only didactic material.	Doctors (obstetricians, neonatologist, anaesthetist), midwives, interns and students<br><br>N not stated	Face-to-face workshops	Three 1-day workshops over a period of 6 months. Other components were continuous over the time of the trial	Vacuum

Study	Details of intervention strategy	Health provider included in intervention strategy / Number (N)	Mode of delivery of intervention strategy	Frequency and duration of intervention strategy	AVB instrument used in strategy
Mogilevkina 2022	Staff of participating hospitals were trained using the Advances in Labour and Risk Management (ALARM) International Program (AIP). The course is designed to motivate and encourage health professionals to improve the delivery of emergency obstetric care in LMIC.<br>• It consists of a 5-day course which is based on adult learning principles that promote a collaborative, multidisciplinary approach of working and learning together. It focuses on the five main causes of maternal mortality.<br>• It includes interactive plenary sessions and hands-on skill workshops with obstetrical mannequins	Total = 577 professionals<br>-342 OB-GYNs<br>-235 midwives<br><br>They worked in 28 hospitals. Coverage of staff training was 65.5%(range 28.6% to 100%).	Face-to-face<br><br>Interactive plenary sessions<br><br>Hands-on skill workshops with obstetrical mannequins	On 5-day course (AVB is one session of the whole course; no details on duration of this session)	VE and Forceps
Nolens 2016	Development of a vacuum extraction **guideline, supply of vacuum extraction** equipment, **training** of staff, and **on the job supervision.**<br><br>• Standard operating procedures (SOP) for the use of vacuum extraction and sterilization of Kiwi vacuum extractors were developed.<br>• Group training of residents in the hospital, 1 week before their rotation in labor and delivery ward.<br>• Training consisted of discussion of the SOP on vacuum extraction and sterilization, watching the WHO RHL video on vacuum extraction and skills training on mannequins.<br>• On the job supervision in the week following training.<br>• Continuous supervision on labour ward for 4 months during which most residents had several labour ward shift	Residents<br><br>N = 45	Face-to-face, small groups (4-6 residents), on site<br><br>Group presentation of the guidelines and sterilization SOP<br><br>Video (WHO) on VE and discussion<br><br>Skills training on mannequins<br><br>Continuous onsite supervision for 4 months during labour ward shifts	4 months initial training accompanied by yearly theory and a yearly skills training session	Vacuum (Kiwi OmniCup)

Nolens 2016, continued	• After completion of this program with a duration of 4 months, training continued according to the existing curriculum complemented with the new SOP on vacuum extraction.<br>• The existing curriculum consisted of a yearly theory session and a yearly skills training session on mannequins per year group (there were 3 year groups: year 1, 2 and 3).<br>• Both the previously trained providers (residents in year 1, 2 and 3) and the newly arrived (year 1) residents were trained according to the existing curriculum after the initial training period of four months.<br>• Yearly refresher course (theoretical and skills-mannequin training) was provided to all residents and final-year medical students.				

Study	Details of intervention strategy	Health provider included in intervention strategy / Number (N)	Mode of delivery of intervention strategy	Frequency and duration of intervention strategy	AVB instrument used in strategy
Skinner 2017	Residency training program: policy requiring residents to develop competency in outlet, low, mid-cavity, and rotational instrumental birth.<br>• Before 2010, resident teaching in forceps and vacuum births was predominantly case based with additional informal lectures and mannequin simulation.<br>• In 2010, formalized lecture series and mannequin simulation training were implemented preceding mandatory instrumental credentialing for all obstetric residents.<br>• Credentialing required residents to be directly supervised by senior obstetricians in human instrumental birth until assessed as competent for unsupervised practice.<br>• Residents could only be credentialed in vacuum birth after being first credentialed in forceps birth.<br>• All residents were required to meet with training supervisors at 3-monthly intervals to review credentialing documents and implement remedial pathways if credentialing was not achieved in an appropriate timeframe.	Residents<br><br>N not stated	Lecture series, mannequin simulation, performance of supervised human instrumental birth	5 years (ongoing post-training intervention)	Forceps, Vacuum

Study	Details of intervention strategy	Health provider included in intervention strategy / Number (N)	Mode of delivery of intervention strategy	Frequency and duration of intervention strategy	AVB instrument used in strategy
Solt 2011	Day time coverage of labour and delivery (L&D) by a specially recruited senior generalist obstetrician with 35 years' clinical experience.<br>• The obstetrician was involved in teaching all aspects of obstetrical management and appropriate use of instrumental assisted delivery.<br>• The attending physician worked as the L&D laborist 4 days (40 hours) in a week and performed night call approximately 2 to 3 times per month.	Residents<br><br>N not stated	On-site labour and delivery coverage and resident training by obstetrician	2 years	Forceps (with predominance of Simpson or Luikart-Simpson forceps)<br><br>Vacuum

Study	Details of intervention strategy	Health provider included in intervention strategy / Number (N)	Mode of delivery of intervention strategy	Frequency and duration of intervention strategy	AVB instrument used in strategy
Sorensen 2010	2-day Advanced Life Support in Obstetrics (ALSO) provider course.<br>• The course consisted of lectures, workshops and case discussions on the major topics of emergency obstetric and neonatal care (EmONC).<br>• Each workstation lasted 75 minutes and case discussion 30-45 minutes.<br>• Intrapartum monitoring was taught in small groups discussing 5 partograph-based case stories with focus on assessment of fetal heart rate and progress of labor.<br>• Artificial rupture of membranes and oxytocin augmentation were emphasized as actions to perform before considering caesarean delivery.<br>• In the workstation on vacuum delivery, hands-on skills were practiced using pelvic and fetal mannequins and the “Kiwi OmniCup,” a hard plastic cup with a pump integrated in the handle.<br>• Neonatal resuscitation was covered by both a lecture and a workshop.	Doctors, assistant medical officers, nurse-midwives<br><br>N = 26	Face-to-face, workshop, lectures, simulations with mannequins	2 days	Vacuum

Study	Details of intervention strategy	Health provider included in intervention strategy / Number (N)	Mode of delivery of intervention strategy	Frequency and duration of intervention strategy	AVB instrument used in strategy
Takeda 2018	Forceps delivery simulation training.<br>• Consisting of a lecture with forceps delivery videos, a demonstration and simulation training experience.<br>• The lecture is about pelvic examination for precise assessment of fetal head descent based on trapezoidal fetal station and is supplemented by forceps delivery videos.<br>• Then, forceps delivery is demonstrated by instructors, using a pelvic model and fetal doll.<br>• Finally, trainees attempt a simulated forceps delivery under the supervision of a specialist.	Obstetrician<br><br>N = 1	Face-to-face lecture with forceps delivery videos, a demonstration, and practicing in simulation pelvic model	Not stated	Utokyo Naegele Forceps
