# Supplementary material for: Interventions to reintroduce or increase assisted vaginal births: a systematic review of the literature
Source: BMJ Open. 2023 Feb 14;13(2):e070640. doi: 10.1136/bmjopen-2022-070640 (PMC9930566; doi:10.1136/bmjopen-2022-070640)
Supplement: Supplementary data [file bmjopen-2022-070640supp007.pdf]

Supplementary file 7. Types of outcomes reported in the 16 included studies

Outcome	N studies (Total= 16)	References
OVD rates		
VE rates	14	Ameh 2014, Bardos 2017, Becker 2020, Cottrell 2021, Dmello 2021, Dominico 2018, Geelhoed 2018, Gulmezoglu 2016, Mogilevkina 2022, Nolens 2016, Skinner 2017, Solt 2011, Sorensen 2010, Takeda 2018
Forceps rates	7	Bardos 2017, Becker 2020, Cottrell 2021, Mogilevkina 2022, Skinner 2017, Solt 2011, Takeda 2018
Overall OVD rates	5	Berglund 2010, Becker 2020, Cottrell 2021, Dumont 2013, Solt 2011
Caesarean rates		
No information	2	Gulmezoglu 2016, Skinner 2017[a]
Overall rate	12	Ameh 2014, Bardos 2017, Becker 2020, Berglund 2010, Cottrell 2021, Dmello 2021, Dominico 2018, Geelhoed 2018, Mogilevkina 2022, Nolens 2016, Solt 2011, Takeda 2018
Antepartum/before active labour CS	2	Dumont 2018, Sorensen 2010
Emergency CS	1	Sorensen 2010
Intrapartum CS	1	Dumont 2018
CS for prolonged labour	1	Sorensen 2010
CS due to obstructed labor	1	Nolens 2016
Maternal outcomes		
None	6	Becker 2020, Berglund 2010, Dominico 2018, Gulmezoglu 2006, Sorensen 2010, Takeda 2018
Maternal mortality	6	Ameh 2014[b], Dmello 2021, Dumont 2013, Geelhoed 2018, Mogilevkina 2022, Nolens 2016
3rd/4th degree perineal tears	4	Bardos 2017, Cottrell 2021, Solt 2011, Skinner 2017
PPH or transfusion	3	Dumont 2013, Mogilevkina 2022, Skinner 2017
Uterine rupture	1	Nolens 2016
ICU admission	1	Nolens 2016
Overall maternal complication rate	1	Cottrell 2021
Perinatal outcomes		
None	5	Becker 2020, Dominico 2018, Gulmezoglu 2006, Mogilevkina 2022, Takeda 2018
Stillbirths	6	Ameh 2014, Dmello 2021, Dumont 2013, Geelhoed 2018, Nolens 2016, Sorensen 2010
Neonatal deaths	5	Berglund 2010, Dmello 2021, Dumont 2013, Nolens 2016, Sorensen 2010
Perinatal death	1	Nolens 2016
Apgar scores	3	Bardos 2017, Solt 2011, Sorensen 2010
NICU admission	3	Ameh 2014, Berglund 2010, Nolens 2016
Birth injuries	2	Cottrell 2021, Solt 2011
Neonatal resuscitation	2	Berglund 2010, Sorensen 2010
Composite neonatal complications	2	Cottrell 2021, Skinner 2017

ICU – intensive care unit; NICU – neonatal intensive care unit; PPH – post-partum hemorrhage.
[a] Skinner 2017 reported only CS rates in women who attempted AVB. [b]Ameh 2014 reported direct obstetric case fatality rate.
