# Supplementary material for: Interventions to reintroduce or increase assisted vaginal births: a systematic review of the literature
Source: BMJ Open. 2023 Feb 14;13(2):e070640. doi: 10.1136/bmjopen-2022-070640 (PMC9930566; doi:10.1136/bmjopen-2022-070640)
Supplement: Supplementary data [file bmjopen-2022-070640supp008.pdf]

Supplementary file 8. Summary of effects of the interventions in 16 studies included in the review

All percentages indicate baseline x post-intervention rates or incidence. **Bold** terms in the cells indicate rates or outcomes with statistically significant changes after intervention (compared to baseline), according to primary study authors.

Highlights in last column (only for studies with statistically significant increase in overall AVB rate or rate of use of at least one AVB instrument):
Yellow: Non-significant change in rates of adverse maternal or perinatal outcomes.
Green: Significant decrease in rates of adverse maternal or perinatal outcomes.
Grey: Significant increase in rates adverse maternal or perinatal outcomes

Study	Changes in AVB use	Changes in rate of CS	Changes in adverse maternal & perinatal outcomes
Ameh 2014, Kenya	**VE rate: significant increase** (from 0.2% to 1%, at 12 months), P< 0.001	Overall CS rate did not change significantly (21.5% x 21.5%, baseline x post-intervention, Table 40).	MM (Direct obstetric case fatality rate): No significant change<br>**NICU admission for birth asphyxia: significant decrease** (48% mean reduction from 7.6% to 4% at 12 months), P = 0.03<br>**Stillbirth rate: Significant decrease (**35% mean reduction at 12 months from 5% to 3%), , P = 0.03<br>Fresh stillbirth rate: No significant change
Bardos 2017, USA	VE rate: No significant change (3.3% to 3.3%)<br>**Forceps rate: Significant increase** (0.6% to 2.6%, aOR 8.44, 95% CI 3.1–23.1). This effect was seen only during day- time (when senior obstetrician was present).	**Overall CS rate: Significant decrease** (27.3% to 24.5%, aOR 0.68, 95% CI 0.55–0.83). This effect was seen only during day-time (when senior obstetrician was present).	3rd-4th degree tears: no significant change<br>5 min Apgar score <7: no significant change
Becker 2020 USA	Overall AVB rate: no significant changes (from 3.2% to 3.1%)<br>**VE rate: significant decrease** (from 2.0% to 1.2%), P < 0.001<br>**Forceps rate: significant increase** (from 1.2% to 2.0%), P = 0.027	Overall CS rates: increase (no statistical analyses) from 27.9%-30.6% in pre-intervention years to 33.5%-33.9% in post-intervention years	NI

Study	Changes in AVB use	Changes in rate of CS	Changes in adverse maternal & perinatal outcomes
Berglund 2010 Ukraine	**Overall AVB rates**: non- significant difference in 2 sites (Lusk 2% to 2%, Lviv 0% to 0%), **significant decrease in 1 site** (Donetsk from 3.7% to 0%), P<0.0001	**Overall CS**: **significant decrease in two sites** (Donetsk 30% to 18.4%, P<0.0001; Lutsk 33% to 12.7%, P<0.0001). Non-significant change in 1 site (Lviv 21.9% to 16.9%).<br>For 3 sites combined: 29.9% to 15.5% (weighted average, no statistical analysis)	**NICU admission: significant decrease in two sites** (Lutsk 7.3% to 4.4%, P= 0.0115; Lviv 6.4% to 2.4%, P= 0.0115); no significant change in one site (Donetsk)<br>Early neonatal death: no significant change (Donetsk: from 13.6% to 9.5%; Lutsk: from 4.6% to 3.0%; Lviv: 6.4% to 0%).<br>**Neonatal resuscitation: significant decrease** (Donetsk: from 13.1% to 7.4%, P = 0.0025; Lutsk: from 2.4% to 0.9%, P<0.0001; Lviv: from 4.5% to 1.1%, P=0.0003).<br>**Hypothermic infants: significant decrease** (Donetsk from 59.5% to 0.6%, P<0.0001; Lutsk from 85.8% to 0.3%, P<0.0001; Lviv from 77% to 0.7%, P<0.0001).
Cottrell 2021 USA	Overall AVB rate: no significant change (10.5 to 10.5%)<br>AVB failure rate (requiring CS): no significant change (1.8% to 1.4%)<br>**VE rate: significant decrease** (from 8.7% to 6.5%, P = 0.002.)<br>**Forceps rate: significant increase** (from 1.8% to 4.4%, P < 0.001)	Overall CS increased (30.4% to 32.1%)-no statistical analysis	3rd-degree tears: no significant change in forceps or vacuum groups<br>4th-degree tears: no significant change in vacuum group<br>**4th-degree tears: significant decrease in forceps** group (RR 0.1, 95% CI 0.01-0.73)<br>**Overall maternal complication rates: significant decrease in forceps** (RR 0.40 95% CI 0.19−0.75). No significant change in VE group.<br>Facial laceration, scalp injury, cephalo-hematoma: no significant change in forceps or vacuum groups<br>Overall neonatal complications: No significant change in forceps or vacuum groups
Dmello 2021 Tanzania	**VE rate: significant increase** (from 0.03% in 2011 to 3.1% in 2019; RR 1.43; 95% CI 1.41 to 1.43)	**Overall rate of CS**: **significant increase** (from 2.3% in 2011 to 17.6% in 2019; RR 1.15; 95% CI 1.14 to 1.15)	During the decade, facility-based:<br>**MMR: significant decrease** (from 154 to 79 per 100 000 live births, RR 0.92, 95% CI 0.9 to 0.95)<br>**Stillbirth rate: significant decrease** (from 26 to 21 per 1000 live births, RR 0.96, 95% CI 0.96 to 0.97)<br>**Neonatal deaths** (sum of deaths among inborn and referred babies)**: significant increase** (from 5.5 in 2011 to a peak of 14.5) in 2014 and then slightly declined to 12.1 in 2019; RR 1.05, 95% CI 1.04 to 1.05)

Study	Changes in AVB use	Changes in rate of CS	Changes in adverse maternal & perinatal outcomes
Dominico 2018 Tanzania	VE rate: Increase (No statistical analyses)<br>1st yr after intervention: 0.15% to 0.88%<br>2nd yr after intervention: 0.15% to 2.11%<br>3rd yr after intervention: 0.15% to 1.54%<br>4th yr after intervention: 0.15% to 1.26%<br>5th yr after intervention: 0.15% to 1.73%	Overall CS rate: Decrease (No statistical analyses)<br>1st yr after intervention: 11.25% to 10.85%<br>2nd yr after intervention: 11.25% to 9.42%<br>3rd yr after intervention: 11.25% to 9.88%<br>4th yr after intervention: 11.25% to 9.80%<br>5th yr after intervention: 11.25% to 11.18%	NI
Dumont 2013 Mali and Senegal	Overall AVB rate: no significant change (all rates < 3%)<br>Senegal: **Overall AVB, significant increase** (1.24% to 2.24%, intervention group, aOR 3.10 (95% CI 1.85-5.20)<br>Mali: Overall AVB, no significant change (3.32% to 1.97% intervention groups, aOR 0.51 (95% CI 0.16 to 1.59)	**Intrapartum CS: significant decrease** (aOR 0.87, 95% CI 0.82 - 0.92, P<0.0001)<br>**Emergency antepartum CS: significant increase** (OR 1.33; 1·19–1·50, p<0·0001) mainly due to PE/E.	**Hospital MM: significant decrease** (OR 0.85, 95% CI 0.73–0.98)<br>Transfusions: marginally significant increase (OR 1·44, 95% CI 0.99–2.11, p=0.06)<br>**Neonatal mortality < 24 h: significant decrease** (adjusted OR 0·74; 95% CI 0.61–0.90)<br>Stillbirth: no significant change
Geelhoed 2018 Mozambique	VE rate: Increase (No statistical analyses)<br>1st yr after intervention:0.2% to 1.0%<br>2nd yr after intervention: 0.2% to 2.3%<br>3rd yr after intervention: 0.2% to 2.2%	Overall CS rate: Decrease (No statistical analyses)<br>1st yr after intervention: 2.7% to 2.1%<br>2nd yr after intervention: 2.7% to 2.1%<br>3rd yr after intervention: 2.7% to 1.8%	Institutional maternal mortality rate: reduction from 126/100,000 births (baseline) to 49/100,000, 48/100,000 (1st yr), 44/100,000 (1st, 2nd and 3rd yr). No statistical analysis<br>Institutional stillbirth rate: reduction from 17.7/1000 newborns (baseline) to 14.9/1000 to 12.4/1000 to 12.2/1000 (1st, 2nd, 3rd yr). No statistical analysis.

Study	Changes in AVB use	Changes in rate of CS	Changes in adverse maternal & perinatal outcomes
Gulmezoglu 2006 Mexico	VE rate: no significant changes in intervention or control groups from baseline to adjusted end-of-study rates (intervention: 0.6% to 0.2% and ctrl: 0.4% to 0.1%)	NI	NI
Gulmezoglu 2006 Thailand	VE rate: no significant changes in intervention or control groups from baseline to adjusted end-of-study rates (intervention: 7.5% to 7.7%, and ctrl: 6.3% to 7.7%)	NI	NI
Mogilevkina 2022 Ukraine	**VE rates: significant increase** 0.1% to 0.7% in intervention group vs 0.11% to 0.26% in the control group (Ratio of the OR: 2.86; 95% CI: 1.80-4.57)<br>**Forceps rates: significant decrease** from 0.17% to 0.08% in intervention group vs 0.21% to 0.05% in the control group (Ratio of the OR: 1.80; 95% CI: 1.00-3.25)	**Overall CS rates: significant increase** (13.12% to 15.9%) in intervention group	MM: non-significant change<br>PPH > 1000 ml: non-significant change<br>Post-partum hysterectomy: non-significant change<br>**Blood transfusion: significant decrease** (OR: 0.56; 95%CI: 0.48-0.65)<br>**Plasma transfusions: significant decrease** (OR: 0.70; 95%CI: 0.63-0.78)<br>**Uterus explorations: significant decrease** (OR: 0.64; 95%CI: 0.59-0.69)
Nolens 2016 Uganda	**VE rate: significant increase** (0.6% to 2.4%, P<0.01)	**Overall CS rate: significant Increase** (28.2% to 30.2%, P< 0.01)<br>CS rate due to obstructed labor: No change (6.0% to 6.0%)<br>Slight change in indications for CS:<br>The percentage of CS for obstructed labour decreased from 23.1% to 20.0% (OR 0.92, 95% CI 0.84-1.01)	**Uterine rupture: significant decrease** (1.1% to 0.8%, P< 0.01; OR 0.75 95% CI 0.61-0.92)<br>Maternal ICU admission due to intrapartum complication: no significant change (1.9% to 1.8%)<br>Maternal death due to intrapartum complication: no significant change (0.3% to 0.2%)<br>Overall maternal deaths: no significant change (0.8% to 0.7%)<br>**Total perinatal mortality: significant decrease** (9.2% to 8.4%, P=0.02)<br>**Intrapartum stillbirths: significant decrease** (3.4% to 2.6%, P< 0.01; OR 0.76 95% CI 0.67-0.85)<br>Term neonatal deaths: no significant change (1.3% to 1.4%)<br>**Term admission to NICU: significant increase** (8.7% to 10.0%, P< 0.01, OR 1.16 95% CI 1.08-1.25)

Study	Changes in AVB use	Changes in rate of CS	Changes in adverse maternal & perinatal outcomes
Skinner 2017<br>Australia	**VE rates decreased significantly** (from 7.98%-last yr pre-intervention to 6.0%-last yr after intervention)<br>**Forceps rate increased significantly** (from 4.75%-last yr pre-intervention to 9.77%-last yr after intervention)<br>Rate of failed AVB: no significant change	NI (only reported CS in women who attempted AVB)	**PPH: significant decrease** (-1.3, 95% CI -2.07 to -0.49)<br>3rd-4th degree tears: no significant change (-1.04, 95% CI -3.1 to 1.00)<br>Composite neonatal morbidity: no significant change (-0.18, 95% CI -0.38 to 0.02)
Solt 2011<br>USA	Overall AVB rates. Non-significant change (11.3% to 10.6%)<br>**VE rates: significant decrease** (from 6.4% to 2.9%, P < 0001)<br>**Forceps rate: significant increase** (from 4.9% to 7.8%, P < 0001)	Overall CS: no significant change (25.5% to 27.3%)	3rd and 4th-degree tears: non-significant change (3.6% to 3.1%)<br>5 min Apgar <7 : non-significant change (1.9% to 2.4%)<br>Birth injuries: non-significant change (0.2% to 0.3%)<br>Umbilical cord pH < 7.1: significant decrease (2.4% to 1.5%)
Sorensen 2010<br>Tanzania	VE rates: No change (0% to 0%).	CS before active labor: no significant change (from 2.0% to 1.8%)<br>Emergency CS: no significant change (from 6.9% to 6.3%)<br>CS for prolonged labor: no significant change (from 5.1% to 4.4%)	**Death of liveborn with Apgar ≥ 4: significant decrease** (from 1.1% to 0%, p=0.03)<br>Neonatal deaths: No significant change (1.2% to 0.4%)<br>1-min Apgar <7: No significant change (6.4% to 6.9%)<br>Stillbirths: No significant change (1.7% to 2.5%)<br>Neonatal resuscitation: No significant change (3.0% to 2.4%)<br>Babies with mothers within 10 minutes: significant increase (from 5.6% to 71.5%)

Study	Changes in AVB use	Changes in rate of CS	Changes in adverse maternal & perinatal outcomes
Takeda 2018 Japan	VE rates: decrease (from 1.2%-13.3% in the 10 pre-intervention years to 2.3% after the intervention). No statistical analyses<br>Forceps rate: increase (from 0%-2,0% in the 10 pre-intervention years to 4.3% in post-intervention year). No statistical analyses	Overall CS rates: decrease after intervention (from 7.2%-25.6% in pre-intervention years to 22.2% in post-intervention year). No statistical analyses	NI

aOR: Adjusted odds ratio. AVB: Assisted vaginal birth. CI: Confidence interventional. CS: Cesarean section. Difce: Difference. ICU: Intensive care unit. MM: Maternal mortality. MMR: Maternal mortality ration. NI: No information. NICU: Neonatal intensive care unit. OR: Odds ratio. PPH: Post-partum haemorrhage. RR: Relative risk. VE: Vacuum extraction. Yr: Year
