# Supplementary material for: Interventions to reintroduce or increase assisted vaginal births: a systematic review of the literature
Source: BMJ Open. 2023 Feb 14;13(2):e070640. doi: 10.1136/bmjopen-2022-070640 (PMC9930566; doi:10.1136/bmjopen-2022-070640)
Supplement: Author's manuscript [file bmjopen-2022-070640.draft_revisions.pdf]

BMJ Open

BMJ Open is committed to open peer review. As part of this commitment we make the peer review history of every article we publish publicly available.

When an article is published we post the peer reviewers' comments and the authors' responses online. We also post the versions of the paper that were used during peer review. These are the versions that the peer review comments apply to.

The versions of the paper that follow are the versions that were submitted during the peer review process. They are not the versions of record or the final published versions. They should not be cited or distributed as the published version of this manuscript.

BMJ Open is an open access journal and the full, final, typeset and author-corrected version of record of the manuscript is available on our site with no access controls, subscription charges or pay-per-view fees (http://bmjopen.bmj.com).

If you have any questions on BMJ Open's open peer review process please email info.bmjopen@bmj.com

Interventions to reintroduce or increase assisted vaginal births: a systematic review of the literature

Journal:	*BMJ Open*
Manuscript ID	bmjopen-2022-070640
Article Type:	Original research
Date Submitted by the Author:	30-Nov-2022

Complete List of Authors:	Torloni, Maria Regina; Federal University of Sao Paulo, Department of Medicine<br>Opiyo, Newton; World Health Organization, UNDP/UNFPA/UNICEF/WHO/World Bank Special Programme of Research, Development and Research Training in Human Reproduction (HRP) Department of Reproductive Health and Research<br>Altieri, Elena; World Health Organization<br>Sobhy, Soha ; Queen Mary University of London,<br>Thangaratinam, Shakila; Queen Mary University of London, BARC (Barts Research Centre for Women's Health), Blizard Institute, Barts and the London School of Medicine and Dentistry; University of Birmingham, Institute of Metabolism and Systems Research<br>Nolens, Barbara; Canisius Wilhelmina Hospital,<br>Geelhoed, D. ; Tete Provincial Hospital<br>Betran, Ana Pilar; World Health Organization, Reproductive Health and Research

Keywords:	OBSTETRICS, PERINATOLOGY, PUBLIC HEALTH

SCHOLARONE™
Manuscripts

I, the Submitting Author has the right to grant and does grant on behalf of all authors of the Work (as defined in the below author licence), an exclusive licence and/or a non-exclusive licence for contributions from authors who are: i) UK Crown employees; ii) where BMJ has agreed a CC-BY licence shall apply, and/or iii) in accordance with the terms applicable for US Federal Government officers or employees acting as part of their official duties; on a worldwide, perpetual, irrevocable, royalty-free basis to BMJ Publishing Group Ltd ("BMJ") its licensees and where the relevant Journal is co-owned by BMJ to the co-owners of the Journal, to publish the Work in this journal and any other BMJ products and to exploit all rights, as set out in our licence.

The Submitting Author accepts and understands that any supply made under these terms is made by BMJ to the Submitting Author unless you are acting as an employee on behalf of your employer or a postgraduate student of an affiliated institution which is paying any applicable article publishing charge ("APC") for Open Access articles. Where the Submitting Author wishes to make the Work available on an Open Access basis (and intends to pay the relevant APC), the terms of reuse of such Open Access shall be governed by a Creative Commons licence – details of these licences and which Creative Commons licence will apply to this Work are set out in our licence referred to above.

Other than as permitted in any relevant BMJ Author's Self Archiving Policies, I confirm this Work has not been accepted for publication elsewhere, is not being considered for publication elsewhere and does not duplicate material already published. I confirm all authors consent to publication of this Work and authorise the granting of this licence.

For peer review only

Interventions to reintroduce or increase assisted vaginal births: a systematic review of the literature

Maria Regina Torloni[a], Newton Opiyo[b], Elena Altieri[c], Soha Sobhy[d], Shakila Thangaratinam[e], Barbara Nolens[f], Diederike Geelhoed[g], Ana Pilar Betrán[b]

a. Evidence Based Healthcare Post-Graduate Program, São Paulo Federal University, São Paulo, Brazil. Email: torlonimr@gmail.com

b. UNDP-UNFPA-UNICEF-WHO-World Bank Special Programme of Research, Development and Research Training in Human Reproduction (HRP), Department of Sexual and Reproductive Health and Research, World Health Organization, Geneva, Switzerland
Email: opiyon@who.int Email: betrana@who.int

c. Behavioral Insights Unit, World Health Organization, Geneva, Switzerland
Email: altierie@who.int

d. Academic clinical lecturer, Women's Health Research Unit, Wolfson Institute of Population Health, Queen Mary University of London, UK. Email: soha.sobhy@qmul.ac.uk

e. Co-Director of WHO Collaborating Centre for Global Women's Health, Institute of Metabolism and Systems Research, University of Birmingham, UK
Email: s.thangaratinam.1@bham.ac.uk

f. Canisius-Wilhelmina Hospital, Nijmegen, the Netherlands (consultant obstetrician/gynaecologist). Mulago National Referral Hospital, Kampala, Uganda (visiting)
Email: bnolens@yahoo.com

g. Formerly (2013-2017), senior technical advisor SRHR to Provincial Directorate of Health, Cidade de Tete, Tete Province, Mozambique, for Danish Ministry of Foreign Affairs (DANIDA) Email: geelhoed.d.w@gmail.com

Corresponding author: Maria Regina Torloni, Evidence Based Healthcare Post-Graduate Rua: Napoleão de Barros, 865 - Vila Clementino, CEP 04024-002 São Paulo – SP, Brazil. Tel: 55 11 5576-4848. Email: torlonimr@gmail.com

Word count: 4250. Tables: 4, Figure: 1, References: 59

For peer review only

ABSTRACT

Objective: To synthesize the evidence from studies that implemented interventions to increase/reintroduce the use of assisted vaginal births (AVB).
Design: Systematic review (PROSPERO#CRD42020215224)
Eligibility criteria: We included experimental, semi-experimental, and observational studies that reported any intervention to reintroduce/increase AVB use.
Data sources: We searched PubMed, EMBASE, CINAHL, LILACS, Scopus, Cochrane, WHO Library, Web of Science, ClinicalTrials.gov and WHO.int/ictrp through September 2021.
Risk of bias: For trials, we used the Cochrane-EPOC tool; for other designs we used ROBINS-I.
Data extraction and synthesis: Due to heterogeneity in interventions, we did not conduct meta-analyses. We present data descriptively, grouping studies according to settings: high-income countries (HICs) or low-middle income countries (LMICs). We classified direction of intervention effects as a)statistically significant increase or decrease, b)no statistically significant change, or c)statistical significance not reported in primary study. We provide qualitative syntheses of the main barriers and enablers for success of the intervention.
Results: We included 16 studies (10 from LMICs), mostly of low or moderate methodological quality, which described interventions with various components (e.g. didactic sessions, simulation, hands-on training, guidelines, audit/feedback). All HICs studies described isolated initiatives to increase AVB use; 9/10 LMIC studies tested initiatives to increase AVB use as part of larger multicomponent interventions to improve maternal/perinatal healthcare. No study assessed women´s views or designed interventions using behavioural theories. Overall, interventions were less successful in LMICs than in HICs. Increase in AVB use was not associated with significant changes in adverse maternal or perinatal outcomes. The main barriers to the successful implementation of the initiatives were related to staff and hospital environment. **Conclusions:** There is insufficient evidence to indicate which intervention, or combination of interventions, is more effective to safely increase AVB use. More research is needed, especially in LMICs, including studies that design interventions taking into account theories of behaviour change.

Keywords: Assisted Vaginal Birth; Obstetrical Forceps; Vacuum Extraction, Obstetrical; Simulation Training; Emergency Obstetric Care; Systematic Review

Article summary: Strengths and Limitations

- This is the first systematic review on initiatives to increase the use of assisted vaginal birth, a life-saving procedure that has been steadily declining, especially in low- and middle-income countries.
- We conducted a comprehensive literature search (including grey literature), without date, publication status or language restrictions, in 10 electronic databases.
- The systematic review followed strict methodological standards including double data extraction and quality assessment of all studies.
- Many of the included studies had limited information on participant characteristics, lacked adequate statistical analyses, and did not report important outcomes.
- We created a subjective classification to categorize the core components of the interventions.

For peer review only

INTRODUCTION

Assisted vaginal birth (AVB) refers to a vaginal birth conducted with the help of an instrument, such as a forceps or a vacuum extractor (VE). Common indications for AVB include prolonged second stage, maternal exhaustion or medical indications (e.g. maternal heart disease), and suspected or confirmed foetal compromise in the second stage of labour.[1] AVB can reduce maternal and perinatal mortality and morbidity associated with complications in the second stage of labour.[2] AVB can also avoid a second-stage caesarean section (CS) and its associated short- and long-term morbidity for the mother and baby, especially in low- and middle-income countries (LMICs).[3-9] Compared to second-stage CS, AVB is associated with lower risks of haemorrhage, post-operative infection, faster maternal recovery, shorter hospital stay, and cost-savings for health systems.[10-13] Moreover, women are more likely to have a spontaneous vaginal birth in their next pregnancy after an AVB than after a CS.[14]

The benefits associated with AVB may be greater in low-resource settings where many births occur in primary care facilities with limited access to emergency obstetric care, and where the need for a CS requires a time-consuming referral to a higher level health facility, where the safety of a CS cannot be guaranteed.[13 15-17] In these settings, AVB can be a life-saving intervention for mothers and babies who are facing second stage complications. The World Health Organization (WHO) and other UN agencies have included AVB as one of the seven critical signal functions of basic emergency obstetric care (EmOC), and one of the nine signal functions of comprehensive EmOC.[18] However, reports indicate that, in most LMICs, AVB is the least likely function to be performed in primary care facilities. [19-21]

While CS rates have continued to increase in the last decades, rates of AVB have declined, particularly in LMICs.[22 23] Although unreliable data about type of birth may affect the accuracy of estimates especially in low income settings, the prevalence of AVB in high income countries (HICs) is currently 5%-20%, compared to <5% in most LMICs, with many countries having <1% of all women giving birth by AVB.[13] The reasons for the decline of AVB vary across settings and include the increasing use of epidurals for labour analgesia, lack of skilled operators and functioning equipment, concerns about trauma to the neonate and mother, fear of mother-to-child transmission of HIV, fear of litigation in case of complications, and policies that exclude some professional cadres (e.g. midwives) from performing AVB. [2 13 24 25]

In recent years, several investigators have reported efforts to increase or reintroduce AVB, particularly in LMICs, using a variety of strategies targeted at perceived barriers to the use of this procedure.[26-31] However, to our knowledge, there are no systematic reviews on this topic.

The general objective of this review was to identify, critically appraise, and synthesize the evidence from studies which implemented interventions or programs to increase or reintroduce AVB use. The specific objectives of the review were a) to assess the effectiveness and safety of strategies aimed at increasing or reintroducing AVB use, b) to identify the core elements of interventions to increase AVB use, and c) to describe the main barriers and enablers to the implementation of these initiatives, according to study authors.

For peer review only

METHODS

The protocol for this review was registered in PROSPERO (CRD42020215224).[32] We report the review according to PRISMA guidelines.[33].

Patient and Public Involvement

Patients and the public were not involved in the design, or conduct, or reporting, or dissemination plans of our systematic review.

Ethics approval

This study does not involve human participants. Ethical approval was not required for this systematic review because all data came from information freely available in the public domain.

Types of studies

We included studies that assessed the effects (benefits and harms) of interventions to reintroduce or increase AVB use with any of the following designs: randomized, semi-randomized, cluster randomized, stepped wedge, or non-randomized trials, cohort studies, cross-sectional studies, before and after intervention studies, interrupted time-series, large scale intervention case studies (implementation research studies) and mixed-methods (quantitative and qualitative) studies. Studies conducted in all settings (low-, middle-, and high-income countries) were eligible for inclusion. We excluded studies presented only as congress abstracts as these have insufficient information about the intervention and/or outcomes.

Participants

Studies involving health personnel who provide labour and delivery care to pregnant women (including qualified or trainee physicians, obstetricians, nurses, midwives, and health assistants) were eligible for inclusion. We also included studies that implemented training programs or quality improvement (QI) interventions to reintroduce or increase the use of AVB targeted at health systems, healthcare facilities, or hospitals that provide labour and delivery care. We excluded studies that involved only undergraduate students.

Interventions

Studies involving any intervention to introduce, reintroduce, or increase AVB use with any instrument were eligible for inclusion. Educational or training programs in any format and of any duration were eligible, including individual or group training, workshops, or courses conducted online or face-to-face, onsite or offsite, involving simulation, hands-on training, and on the job supervision and auditing. We also included studies that used other strategies to increase AVB use, like different models of care, task-shifting, reorganization of staff, use of opinion leaders or champions, and the provision or continued availability of functional equipment. We included studies that reported the use of specific, isolated interventions to increase AVB use as well as studies that promoted this intervention as part of larger educational/training or QI initiatives that included other components of EmOC. Eligible comparators were no intervention, usual care or practice in accordance with local protocols, or alternative interventions to reintroduce or increase the use of AVB, as reported in the primary studies.

For peer review only

Outcome measures

The primary outcome was change in the rate of AVB (overall or by specific instrument). Secondary outcomes were changes in CS rates (overall, intrapartum, and second stage CS), maternal mortality and morbidity, neonatal mortality and morbidity, maternal birth experience, health provider satisfaction with training program or QI intervention, and change in health provider AVB knowledge or skills. Studies that only reported secondary outcomes without data on the primary outcome were not included.

We only included in the review studies that stated that increase in AVB use was one of their objectives, or that change in AVB rate was one of the study outcomes (in the Methods section). All studies also had to provide data (before and after the intervention, or in the intervention and control groups) on AVBs performed by the study participants (Kirkpatrick level-3) or AVB prevalence in the hospital or setting (Kirkpatrick level-4). [34] We excluded studies that only provided data on participants´ satisfaction with the intervention (Kirkpatrick level-1) or their knowledge acquisition (Kirkpatrick level-2). [34]

Search strategy

We searched MEDLINE/PubMed, EMBASE, CINAHL, LILACS, Scopus, the Cochrane Library, WHO Library, and Web of Science using the following combination of free text and MeSH terms adapted to each database: "forceps" OR "vacuum extraction" Or "ventouse" OR "operative vaginal delivery" OR "assisted vaginal birth" AND "training" OR "programs" OR "quality improvement initiatives" (complete search strategies in Supplementary file 1). We also searched MEDLINE/PubMed using the names of specific EmOC training programs (ROBuST, ALSO, PRONTO, ESMOE). We searched two clinical trials registries (International Clinical Trials Registry Platform www.who.int/ictrp/en/ and ClinicalTrials.gov (https://clinicaltrials.gov/) for ongoing studies or completed unpublished studies. We also searched websites of key organizations involved in EmOC training (WHO, UNFPA, UNICEF, JPHIEGO, FIGO, ICM, RCOG, Pathfinder, Population Council, Advanced Life Support Group, and Childhealth Advocacy International). The searches were conducted in December 2019 and updated in September 2021. The searches were done without any language, date, or publication status restrictions. We complemented the electronic searches by screening the reference lists of all studies selected for full-text reading and related reviews, and by contacting authors of relevant abstracts for additional, potentially relevant, studies.

Process of study selection and data extraction

The identified references were entered in Covidence (https://www.covidence.org/). Four reviewers (MRT, NO, SS, APB) working in pairs independently screened titles and abstracts of all unique citations and applied the pre-specified study eligibility criteria to select studies for full-text reading. Three independent reviewers (MRT, NO, APB) read these studies and selected those that fulfilled the aforementioned inclusion criteria. The same three reviewers extracted data on key information (study design, setting, participants, interventions, outcomes, and barriers and enablers to intervention success described by the authors) independently into a pilot-tested data extraction form specifically designed for this review. In all steps of the selection and extraction process, disagreements were solved by discussion. In case consensus could not be reached, a fourth reviewer was called to arbitrate.

For peer review only

Quality assessment of included studies

We used the criteria suggested by the Cochrane Effective Practice and Organisation of Care (EPOC) group to assess the risk of bias of the randomized trials included in the review.[35] Each trial was rated as having a "High", "Low" or "Unclear" overall risks of bias based on nine domains: random sequence generation and allocation concealment (selection bias), baseline outcome measurements and baseline characteristics, blinding of participants and personnel (performance bias), blinding of outcome assessment (detection bias), contamination, incomplete outcome data (attrition bias), and selective reporting (reporting bias). For other study designs we used the "Risk of Bias for Non-Randomised Studies of Interventions" (ROBINS-I) tool.[36] Risk of bias domains in this tool include the likelihood of bias attributable to confounding, selection of participants, classification of interventions, deviations from intended interventions, missing data, measurement of outcomes, and selection of reported results. Based on this approach, the overall risk of bias in each of these studies was rated as "Low risk", "Moderate risk", "Serious risk", "Critical risk", or "No information". Two review authors (NO, APB) independently conducted all quality assessments in duplicate. Disagreements were resolved by discussion, with the participation of a 3rd reviewer when needed. We did not exclude studies based on their risk of bias ratings.

Data synthesis

We intended to pool data from similar study designs into meta-analyses to assess the effectiveness of the intervention on the outcomes of interest. However, this was not possible because the interventions were very heterogeneous, with most individual studies using multiple different components in their educational and training interventions. Therefore, we present the data descriptively, grouping studies into two main categories according to the setting where they were conducted, i.e., LMICs or HICs, according to the World Bank.[37] We classified the direction of effect of the intervention on each outcome as a) statistically significant increase or decrease, b) no statistically significant change, or c) statistical significance not reported by the primary study authors. Statistical significance was judged using standard statistics (P values or 95% confidence intervals reported in primary studies). We list the main barriers and enablers related to the implementation or success of the intervention as reported by the study authors.

RESULTS

We identified 13,219 unique records from electronic databases and clinical trials registries. We excluded 13,177 records following review of titles and abstracts, and retrieved the full texts of the remaining 42 records for detailed eligibility assessment; 32 of these records were excluded due to various reasons (Supplementary file 2). We included in the review six additional studies not captured by the electronic search that were identified from screening the reference lists of studies selected for full text reading. Sixteen studies (15 published papers[26-31 38-46] and one PhD level thesis available online[47]), presenting 18 datasets, fulfilled the selection criteria and were included in the review (Figure 1).

Main characteristics and quality of included studies

Six studies were conducted in three HICs (US=4[27 38 39 44], Australia=1[43], Japan=1[45]). The other ten studies[26 28-31 40-42 46 47] were conducted in nine LMICs (Kenya, Mali, Mexico, Mozambique, Senegal, Tanzania, Thailand, Uganda and Ukraine). Most of these countries

For peer review only

were in sub-Saharan Africa (Tanzania=3[26 30 42], Kenya=1[47], Mozambique=1[31], Senegal and Mali=1[40], Uganda=1[29]). Two studies [40 41] were conducted in two different LMICs and their results are presented in separate datasets. The other 14 studies were conducted in a single country. Most HIC studies (5/6) were conducted in single facilities while most LMIC studies (8/10) were multisite (ranging from 3 to 82 facilities). Most of the studies involved public facilities located in urban areas, included more than one type of healthcare professionals, and used initiatives to increase the use of VE, or forceps and VE. Midwives/nurses were not included in any of the HIC studies but participated in the AVB training interventions in all of the LMIC studies except one[29] (which focused exclusively on training obstetrics and gynaecology residents). The studies were published mostly in 2010-2019 (11/16) and used non-randomized designs (13/16). Most studies were judged to have a moderate (8/16) or serious (3/16) risk of bias (Table 1). Study details and quality assessments are provided in Supplementary files 3 and 4. Fourteen studies reported baseline rates of VE that ranged from 0% to 8.7%, seven studies reported baseline rates of forceps that ranged from 0% to 5.2%, and five studies presented baselines rates of AVB in general that ranged from 1.8% to 11.3%. Overall baseline CS rates were reported by five studies and ranged from 2.6% to 30.6%. In general, LMICs had the lowest and HICs had the highest baseline AVB and CS rates, respectively (Supplementary file 5).

For peer review only

Table 1. Main characteristics of 16 studies on interventions to increase AVB use

Characteristic	N of studies	References
Economic category (UN)[a]		
Low income	3	Dumont 2013 (Mali), Geelhoed 2018, Nolens 2016
Lower middle income	7	Ameh 2014, Berglund 2010, Dmello 2021, Dumont 2013 (Senegal), Dominico 2018, Mogilevkina 2022, Sorensen 2010
Upper middle income	2	Gulmezoglu 2006 (Mexico and Thailand)
High income	6	Bardos 2017, Becker 2020, Cottrell 2021, Skinner 2017, Solt 2011, Takeda 2018
Type of health facility[b]		
Public	12	Ameh 2014, Becker 2020, Berglund 2010, Dmello 2021, Dominico 2018, Dumont 2013, Geelhoed 2018, Gulmezoglu 2006 (Thailand), Mogilevkina 2022, Nolens 2015, Skinner 2017, Sorensen 2010
Private	1	Solt 2011
Public and private	2	Bardos 2017, Gulmezoglu 2006 (Mexico)
Unclear or not stated	2	Cottrell 2021, Takeda 2018
Location of facility		
Urban areas	14	Ameh 2014, Bardos 2017, Becker 2020, Berglund 2010, Cottrell 2021, Dmello 2021, Dumont 2013, Gulmezoglu 2006, Mogilevkina 2022, Nolens 2016, Skinner 2017, Solt 2011, Sorensen 2010, Takeda 2018
Rural areas	1	Geelhoed 2018
Urban and rural areas	1	Dominico 2018
Number of facilities		
1	7	Bardos 2017, Becker 2020, Cottrell 2021, Nolens 2016, Solt 2011, Sorensen 2010, Takeda 2018
3	2	Berglund 2010, Skinner 2017
10-82	7	Ameh 2014, Dmello 2021, Dominico 2018, Dumont 2013, Geelhoed 2018, Gulmezoglu 2006, Mogilevkina 2022
Participants		
Resident physicians	4	Bardos 2017, Cottrell 2021, Nolens 2016, Solt 2011
Other physicians	2	Gulmezoglu 2006, Takeda 2018
>1 type of professional[c]	8	Ameh 2014, Becker 2020, Dmello 2021, Dominico 2018, Geelhoed 2018, Mogilevkina 2022, Skinner 2017, Sorensen 2010
Unclear/not stated	2	Berglund 2010, Dumont 2013
Type of instrument		
Forceps	2	Bardos 2017, Takeda 2018
Vacuum	7	Ameh 2014, Dmello 2021, Dominico 2018, Geelhoed 2018, Gulmezoglu 2006, Nolens 2016, Sorensen 2010
Forceps and vacuum	6	Becker 2020 , Cottrell 2021, Dumont 2013, Mogilevkina 2022, Skinner 2017, Solt 2011
Unclear/not stated	1	Berglund 2010
Year of publication		
2020 or later	4	Becker 2020 , Cottrell 2021, Dmello 2021, Mogilevkina 2022
2010 to 2019	11	Ameh 2014, Bardos 2017, Berglund 2010, Dominico 2018, Dumont 2013, Geelhoed 2018, Nolens 2016, Skinner 2017, Solt 2011, Sorensen 2010, Takeda 2018
2000 to 2009	1	Gulmezoglu 2006
Study design		
Randomized trials	3	Ameh 2014, Dumont 2013, Gulmezoglu 2006
Before & after studies	7	Bardos 2017, Berglund 2010, Dmello 2021, Mogilevkina 2022, Nolens 2016, Sorensen 2010, Takeda 2018
Interrupted time series	3	Dominico 2018, Geelhoed 2018, Skinner 2017
Cohort studies	3	Becker 2020, Cottrell 2021, Solt 2011
Risk of bias		
Low	4	Ameh 2014, Bardos 2017, Dumont 2013, Gulmezoglu 2006
Moderate	8	Berglund 2010, Becker 2020, Dmello 2021, Mogilevkina 2022, Nolens 2016, Skinner 2017, Solt 2011, Sorensen 2010
Serious	3	Cottrell 2021, Dominico 2018, Geelhoed 2018
Unclear[d]	1	Takeda 2018

AVB – assisted vaginal birth; EmOC – emergency obstetrics care. a. One study (Dumont 2013) conducted in two countries: Mali (low-income) and Senegal (lower middle income). b. One study (Gulmezoglu 2006) conducted in public facilities (Thailand) & public and private facilities ((Mexico). c.Doctors and nurse/midwives [Ameh 2018, Dmello 2021, Geelhoed 2018, Mogilevkina 2022]; Doctors, advance level associate clinicians and assistant medical officers [Dominico 2018]; Obstetricians and residents [Becker 2020, Skinner 2004]; Nurse-midwives, assistant medical officers, doctors [Sorensen 2010].. d Risk of bias assessment not done for one study (Takeda 2018) due to insufficient information

Core components of the interventions

Table 2 presents the main components of the interventions described in each study (details in Supplementary file 6). In almost all (9/10) of the studies conducted in LMICs, strategies to increase the use of AVB (mostly VE) were part of larger multicomponent interventions usually focused on improving emergency obstetric care, while all six studies from HICs tested interventions exclusively focused on increasing the use of forceps or both instruments. Didactic training (e.g. lectures, theoretical classes) about AVB was part of the interventions in all studies conducted in LMICs, and in half of those conducted in HICs. The proportion of studies that included AVB simulation training was higher in LMIC (7/10) than in HIC studies (3/6). At least half of the studies conducted in both settings included short (up to 30 days) hands-on training periods when the health care professionals conducted AVB in women under the supervision of instructors. More prolonged (>30 days) on-site hands-on supervision was part of the intervention in most (4/6) of the studies conducted in HICs, but in only less than half (4/10) of the LMIC studies. The provision of AVB guidelines and equipment was part of the intervention in about one third (3/10) of the LMIC studies and in none of the HIC studies. Audit and feedback of AVB rates was a component of the intervention in almost half (4/10) of the LMIC studies and in only one of the HIC studies (Table 2).

For peer review only

Table 2. Main components of interventions to increase AVB use in 16 studies

Study	Instrument of training	Focused only on AVB	Didactic training	Simulation training	Short[a] practical hands-on training	Long[b] onsite, hands-on supervision	AVB guidelines	Provision of Equipment	Audit & feedback
10 Studies conducted in LMICs									
Ameh 2014 (Kenya)	VE	No	Yes	Yes	Yes	Yes	No	Yes	No
Berglund 2010 (Ukraine)	VE or F	No	Yes	No	No	No	Yes	No	Yes
Dmello 2021 (Tanzania)	VE	No	Yes	Yes	Yes	Yes	No	Yes	Yes
Dominico 2018 (Tanzania)	VE	No	Yes	Yes	Yes	Yes	No	No	Yes
Dumont 2013 (Senegal & Mali)	VE or F	No	Yes	Yes	No	No	Yes	No	No
Geelhoed 2018 (Mozambique)	VE	No	Yes	No	Yes	No	No	No	Yes
Gulmezoglu 2006 (Mexico & Thailand)	VE	No	Yes	No	No	No	No	No	No
Mogilevkina 2022 (Ukraine)	VE or F	No	Yes	Yes	No	No	No	No	No
Nolens 2016 (Uganda)	VE	Yes	Yes	Yes	Yes	Yes	Yes	Yes	No
Sorensen 2010 (Tanzania)	VE	No	Yes	Yes	No	No	No	No	No
6 Studies conducted in HICs									
Bardos 2017 (USA)	F	Yes	No	No	Yes	Yes	No	No	No
Becker 2020 (USA)	VE and F	Yes	Yes	Yes	No	No	No	No	No
Cottrell 2021 (USA)	VE and F	Yes	No	No	Yes	Yes	No	No	No
Skinner 2017 (Australia)	VE and F	Yes	Yes	Yes	Yes	Yes	No	No	Yes
Solt 2011 (USA)	VE and F	Yes	No	No	Yes	Yes	No	No	No
Takeda 2018 (Japan)	F	Yes	Yes	Yes	No	No	No	No	No

AVB: Assisted vaginal birth, F: forceps, VE: vacuum extraction
[a] Short: up to 30 days.
[b] Long: More than 30 days.

Effects of the interventions

Table 3 presents the effects of the intervention on the primary outcome (AVB use) as described by the study authors. Only two of the studies[38 40] conducted analyses taking into account possible confounding factors that could have impacted the effects of the interventions on the rates of AVB (Supplementary file 3). In LMICs, the intervention was associated with a significant increase in VE or overall AVB rates in 5 [29 40 42 46 47] of the 12 datasets from the 10 studies. Two additional studies[30 31] reported increases in VE rates but did not conduct statistical analyses. Therefore, in LMICs, interventions were associated with a successful effect (significant increase in AVB) in approximately 42% (5/12) of the datasets. Four of the 6 studies conducted in HICs reported significant increases in forceps use associated with significant decreases in VE rates. The reasons for this effect are not described in the individual studies. One study conducted in Japan[45] also reported an increase in forceps and decrease in VE but did not provide statistical analyses, and one American study[38] reported a significant increase in forceps with non-significant change in VE rates. Since all studies in HICs focused mainly on increasing forceps use, we considered that the interventions were successful (significant increase) in 83% (5/6) of the studies conducted in these settings.

Many studies did not report any of the secondary outcomes of this review (Supplementary file 7). The effects of the interventions on the rates of CS were heterogeneous overall, but most studies only reported changes in overall CS rates and not in second-stage CS rates (Supplementary file 7). In the few studies that provided this information, most of the interventions to increase AVB use were not associated with significant changes in maternal morbidity or perinatal morbimortality (Supplementary file 8). The two studies that assessed participants´ changes in knowledge and skills (Kirkpatrick level-2) reported significant increases in these outcomes immediately after the training intervention.[39 47] Only one study assessed participants´ satisfaction with the intervention (Kirkpatrick level-1) and reported that the trainees (mostly midwives) reacted positively to all lectures and breakout sessions (mean scores 9.3/10).[47] None of the studies assessed maternal birth experience or satisfaction during the interventions.

For peer review only

Table 3. Effectiveness of interventions to increase AVB use

Study	Overall AVB rate	VE rate	Forceps rate
Low- and Middle-Income countries (10 studies, 12 datasets [a])			
Ameh 2014 (Kenya)		Increase*	
Berglund 2010[b] (Ukraine)	NS change		
Dmello 2021 (Tanzania)		Increase*	
Dominico 2018 (Tanzania)		Increase	
Dumont 2013 (Mali) [a]	NS change		
Dumont 2013 (Senegal) [a]	Increase*		
Geelhoed 2018 (Mozambique)		Increase	
Gulmezoglu 2006 (Mexico) [a]		NS change	
Gulmezoglu 2006 (Thailand) [a]		NS change	
Mogilevkina 2022 (Ukraine)		Increase*	NS change
Nolens 2016 (Uganda)		Increase*	
Sorensen 2010 (Tanzania)		NS change	
High income countries (6 studies)			
Bardos 2017 (USA)		NS change	Increase*
Becker 2020 (USA)	NS change	Decrease*	Increase*
Cottrell 2021 (USA)	NS change	Decrease*	Increase*
Skinner 2017 (Australia)		Decrease*	Increase*
Solt 2011 (USA)	NS change	Decrease*	Increase*
Takeda 2018 (Japan)		Decrease	Increase

AVB: Assisted vaginal birth. CS: Cesarean section. NS: non- significant. VE: Vacuum extraction.
a. Multi-country study with two separate datasets. b. Effects in 2 out of 3 sites.
* Statistically significant

Barriers and enablers to the success of the interventions

The main barriers to the success of interventions to increase AVB use were related to healthcare professionals, environmental factors, and training. One of the main barriers reported by study authors was the difficulty in ensuring that the staff acquired and maintained all necessary skills to conduct AVB over time, including obstetrics clinical skills (correct diagnosis for the need to conduct an AVB), technical skills to safely use the instrument, and non-technical skills (communication with the woman and team). Additional barriers were the lack of continued hands-on supervision and practice, lack of institutional support for instrumental births, lack of skilled personnel (staff shortage, high turnover, need for retraining) and instruments, insufficient pain relief for women, and fear of malpractice litigation in case of adverse outcomes. The main enablers to the success of the interventions included issues related to multidisciplinary training, clarity of the roles of healthcare providers, availability of trained and skilled healthcare professionals, and involving women in decision-making (Table 4).

For peer review only

Table 4. Barriers and enablers to the implementation or success of interventions to increase AVB use

**Barrier categories**	**Barriers identified**
Health professionals	Lack of skilled personnel (due to lack of training, high staff turnover, staff shortage)<br>Lack of confidence<br>Lack of supervision in general and especially in night shifts<br>Fear of HIV transmission<br>AVB perceived as a complicated and dangerous intervention<br>Resistance to change, previous attitudes and beliefs not supportive of AVB<br>Fear of malpractice litigation if complications arise
Environment	Lack of clarity on scope of midwifery practice and functions<br>Power struggles in the maternity units<br>Unsupportive work environment
Training	Lack of theoretical and formalized education on AVB<br>Need to learn skills for correct AVB indications and when to stop trying and do a CS<br>Lack of access to repeated opportunities to practice<br>Teacher/mentor preference influence the type of instrument to use and exposure to learning opportunities<br>Lack of access to or of willing clinical mentors<br>Poor communication with mentors
Women – Healthcare provider relationship	Insufficient pain relief for women<br>Lack of acceptance by the women (associated with negative interactions with staff, poor communication, little involvement in decision-making and mistrust of caregiver)
Equipment	Lack of equipment or maintenance of equipment<br>Difficulties related to the need to sterilize instruments (unavailability of the material/equipment for this purpose)
**Enabler categories**	**Enablers identified**
Health professionals	Senior midwifery support<br>Ownership of the initiative/strategy which is facilitated by Opinion Leaders<br>Retention of trained staff
Environment	Provision of opportunities for staff to gain experience<br>In areas where there is high population resistance to CS, AVB when indicated can be popular and acceptable<br>Clarity on the scope of the midwifery practice and functions<br>Establishment of task-shifting strategies<br>Involving senior staff and hospital administrators at the planning stage<br>Local stakeholders (Ministry of Health, hospital administrators) buy-in/endorsement<br>Advocacy efforts<br>Supportive environment including close supervision, pro-active teaching by experienced colleagues enable the development of competencies
Training	Incorporating AVB training in the medical curriculum<br>Training using a range of tools and modalities including videos and simulation (these do not replace hands-on clinical teaching)<br>Training multidisciplinary teams<br>Retraining (in-service)
Women – Healthcare provider relationship	Good communication between healthcare providers and with the woman to foster trust<br>Women's and partners' involvement in decision-making and satisfactory communication between women and providers<br>Acceptance of intervention by the woman is facilitated by positive interactions with staff, respectful care and on-going communication and trust
Equipment	Availability of functioning, user friendly equipment

AVB: Assisted vaginal birth, CS: Caesarean section

For peer review only

DISCUSSION

We found relatively few studies, mostly conducted in LMICs, which reported interventions to increase the use of AVB. Due to the heterogeneity of the interventions, we could not conduct meta-analyses. In LMICs, most studies tested initiatives to increase AVB (mostly VE) as part of larger multicomponent interventions focused on improving maternal/perinatal healthcare. On the other hand, the studies conducted in HICs involved stand-alone interventions focused specifically on increasing AVB use (mostly forceps). Effectiveness differed across studies but, overall, the interventions were less successful at increasing AVB use in LMICs (where baseline rates were lower) than in HICs. Increases in operative births were not associated with significant changes in adverse maternal or perinatal outcomes, in the few studies that reported these outcomes. Caution is recommended in interpreting the findings of this review because most studies had moderate or serious risk of bias. The main barriers described by primary study authors to the success of the interventions were related to training and maintaining the skills of healthcare staff, lack of instruments, and hospital environment factors.

Ensuring that participants acquired the necessary knowledge and skills to perform AVB was a core element of all interventions described in the studies. However, the diversity of methods, duration, and combinations of intervention used in the 16 studies (e.g. didactic training, simulation, hands-on supervision) precluded assessing the effectiveness of each component or combination. Although there seems to be consensus that it is essential to achieve and maintain the knowledge and skills of the professionals caring for obstetric patients, the most effective way to do this remains unknown, particularly in LMICs.[48 49] However, to a certain extent, focusing exclusively on these aspects underestimates other behavioural factors, especially social and structural environmental factors, that need to be addressed for professionals to change their behaviour. Increasing the use of AVB is influenced by decision-making processes and involves behavioural change.[50] The COM-B model, one of the most widely used frameworks to understand behaviour, proposes that three essential conditions (capability, opportunity and motivation) interact to generate any behavior.[51] Interventions that focus exclusively on healthcare providers´ education and training (capability) may ignore other aspects, such as personal motivation and opportunity (social and physical environmental factors), that could play an important role in increasing the use of AVB. Systems thinking approaches may also help to identify behavioural and other drivers that can act as barriers.[52]

At least half of the studies involved other healthcare professionals besides doctors. As the main obstetric care providers in many LMICs, with appropriate training and patient selection, obstetric nurses and midwives can play an important role to increase or reintroduce AVB use, especially in primary healthcare settings. Moreover, in LMICs, AVB in the hands of trained and competent midwives in primary care settings may be an important pathway to empower these professionals. In many LMIC peripheral settings, midwives are expected to manage a large number of births with little support or tools in case of complications, and limited possibilities for timely referral due to problems with transport, roads, and distances. [31]

For peer review only

Midwife-led models of care with back-up from obstetricians, if and when necessary, could be promising approaches to safely reintroduce AVB while ensuring sustainability.[53]
None of the studies reported the views of women, including their birth experience and satisfaction, during the interventions. This is an important dimension of healthcare evaluation since patient experience has been positively associated with clinical effectiveness and safety, and is a driver of healthcare provider decision-making.[54] Dignified and respectful care is an integral part of quality of care and especially relevant when managing complications or emergencies, such as the need for AVB, when women are often most vulnerable. Evidence shows that women recognize the unpredictable nature of birth but, when interventions are needed, they want to retain a sense of personal achievement and control through active decision-making.[55] The assessment of this dimension is important to address women's needs and fears related to AVB, which can directly affect the acceptability of this intervention.

Strengths of the review include its novelty (to our knowledge, this is the first systematic review on this topic), comprehensive literature search including grey literature sources, and adherence to rigorous methodological standards including double data extraction and quality assessment. Limitations of the review pertain mostly to the characteristics of the included studies, such as the paucity of studies that assessed relevant secondary outcomes (e.g. second stage CS), or used adequate statistical analyses (e.g. a priori power calculations or multivariate regression to control for confounders), and the limited information on participant characteristics. Additionally, we cannot exclude the possibility that our search may have missed relevant studies. Finally, we created a subjective classification to categorize the core components of the interventions and we acknowledge that this classification may not be ideal, and that there are other possible ways of doing this.

Based on the findings of this review, there does not seem to be a single recipe to successfully and safely increase AVB use. There is a need for well-designed, sufficiently powered experimental or implementation research studies, especially in LMICs. Ideally, future studies should design interventions after conducting local formative research to identify relevant barriers and facilitators for the desired behavioural change in the full range of relevant cadres and health care settings. Our findings suggest that, especially in studies conducted in LMICs, interventions should include a component of hands-on, onsite, prolonged supervision, as well as continuous training/retraining of new staff. Future studies should also assess and include women´s perceptions and views on AVB in the design of the study, and evaluate participants´ satisfaction with the initiative as one of their outcomes. Additionally, future studies should assess the effects of interventions on the rates of CS in the second stage of labour, and not only on the overall CS rate. Forceps and VE were the only instruments used in the included studies, with a clear predominance of VE in LMICs. The Odón device, a novel instrument that might be easier to use by all cadres of providers, is currently being evaluated for safety and feasibility.[56] This instrument could also be tested in future studies to increase AVD use. Finally, there is a need to assess the economic impact of strategies to increase AVB use, an important point especially for complex, multifaceted interventions that require substantial changes in organizations, staffing, and model of care.

For peer review only

The identification of essential components or key features would be an important step to ensure more effective interventions. The use of Qualitative Comparative Analysis (QCA) is gaining popularity and could accelerate this process.[57] QCA is a methodology used for complex health interventions to identify if certain combinations of intervention features (e.g. type of intervention, contextual characteristics, and how the intervention was delivered) are associated with successful implementation and with improved uptake of the desired outcome, in this case, AVB.[57 58]

The review identified a paucity of studies that were grounded in behavioural theories. This contributed to the fragmented understanding of the specific influencing factors of the intended AVB behaviours, and the lack of identification of additional categories of barriers and drivers for the success of the intervention. Theories of behaviour and behaviour change provide guidance on such categories and their interaction which can help to understand the process of behaviour change before conducting any exploratory piloting and formal testing of interventions.[51 59]

CONCLUSIONS

We identified relatively few, very heterogeneous, studies with multicomponent strategies to increase AVB use. The existing evidence is insufficient to indicate which intervention, or combination of interventions, is more effective to safely increase or reintroduce the use of AVB. More research is needed, in HICs as well as in LMICs, including studies that design interventions after formative research, take into account theories of behaviour change and systems thinking, and that assess all relevant outcomes.

- **Contributorship:** All authors included on a paper fulfil the criteria of authorship. APB, MRT and NO conceived and planned the study, screened and selected the studies for inclusion, conducted data extraction and analyses, and wrote the first draft of the study. EA, SS, ST, BN, and DG made substantial contributions to data analysis and interpretation, and critically reviewed the manuscript for important intellectual content. MRT, NO, EA, SS, ST, BN, DG, and APB approved the final version of the manuscript and agree to be accountable for all aspects of the work in ensuring that questions related to the accuracy or integrity of any part of the work are appropriately investigated and resolved.
- **Competing interests:** Barbara Nolens and Diederike Geelhoed were authors of studies included in the review but were not involved in the analyses of these studies. All authors have no other conflicts of interest to declare
- **Funding:** This research was funded by the UNDP-UNFPA-UNICEF-WHO- Bank Special Programme of Research, Development and Research Training in Human Reproduction (HRP), a cosponsored programme executed by the WHO in the Department of Sexual and Reproductive Health and Research.
- **Data sharing statement:** All data relevant to the study are included in the article or available in the supplementary files.

For peer review only

- **Ethics approval statement:** This study does not involve human participants. Ethical approval was not required for this systematic review because all data came from information freely available in the public domain.

References

1. Operative Vaginal Birth: ACOG Practice Bulletin, Number 219. *Obstet Gynecol* 2020;135(4):e149-e59. doi: 10.1097/AOG.0000000000003764
2. Pattinson RC, Vannevel V, Barnard D, et al. Failure to perform assisted deliveries is resulting in an increased neonatal and maternal morbidity and mortality: An expert opinion. *S Afr Med J* 2018;108(2):75-78. doi: 10.7196/SAMJ.2017.v108i2.12786
3. Rabiu KA, Adewunmi AA, Akinola OI, et al. Comparison of maternal and neonatal outcomes following caesarean section in second versus first stage of labour in a Tertiary Hospital in Nigeria. *Niger Postgrad Med J* 2011;18(3):165-71.
4. Selo-Ojeme D, Sathiyathasan S, Fayyaz M. Caesarean delivery at full cervical dilatation versus caesarean delivery in the first stage of labour: comparison of maternal and perinatal morbidity. *Arch Gynecol Obstet* 2008;278(3):245-9. doi: 10.1007/s00404-007-0548-5
5. Alexander JM, Leveno KJ, Rouse DJ, et al. Comparison of maternal and infant outcomes from primary cesarean delivery during the second compared with first stage of labor. *Obstet Gynecol* 2007;109(4):917-21. doi: 10.1097/01.AOG.0000257121.56126.fe
6. Gurung P, Malla S, Lama S, et al. Caesarean Section During Second Stage of Labor in a Tertiary Centre. *J Nepal Health Res Counc* 2017;15(2):178-81. doi: 10.3126/jnhrc.v15i2.18210
7. Bhargava S, Hooja N, Kala M, et al. Caesarean Delivery in the Second Stage of Labour at a Tertiary Care Hospital. *J Obstet Gynaecol India* 2019;69(6):558-60. doi: 10.1007/s13224-018-1179-4
8. Allen VM, O'Connell CM, Baskett TF. Maternal and perinatal morbidity of caesarean delivery at full cervical dilatation compared with caesarean delivery in the first stage of labour. *BJOG* 2005;112(7):986-90. doi: 10.1111/j.1471-0528.2005.00615.x
9. Cong A, de Vries B, Ludlow J. Does previous caesarean section at full dilatation increase the likelihood of subsequent spontaneous preterm birth? *Aust N Z J Obstet Gynaecol* 2018;58(3):267-73. doi: 10.1111/ajo.12713
10. Murphy DJ, Liebling RE, Verity L, et al. Early maternal and neonatal morbidity associated with operative delivery in second stage of labour: a cohort study. *Lancet* 2001;358(9289):1203-7. doi: 10.1016/S0140-6736(01)06341-3
11. Nolens B, Namiiro F, Lule J, et al. Prospective cohort study comparing outcomes between vacuum extraction and second-stage cesarean delivery at a Ugandan tertiary referral hospital. *Int J Gynaecol Obstet* 2018;142(1):28-36. doi: 10.1002/ijgo.12500
12. Panelli DM, Leonard SA, Joudi N, et al. Severe maternal and neonatal morbidity after attempted operative vaginal delivery. *Am J Obstet Gynecol MFM* 2021;3(3):100339. doi: 10.1016/j.ajogmf.2021.100339
13. Bailey PE, van Roosmalen J, Mola G, et al. Assisted vaginal delivery in low and middle income countries: an overview. *BJOG* 2017;124(9):1335-44. doi: 10.1111/1471-0528.14477
14. Bahl R, Strachan B, Murphy DJ. Outcome of subsequent pregnancy three years after previous operative delivery in the second stage of labour: cohort study. *BMJ* 2004;328(7435):311. doi: 10.1136/bmj.37942.546076.44
15. Sobhy S, Arroyo-Manzano D, Murugesu N, et al. Maternal and perinatal mortality and complications associated with caesarean section in low-income and middle-income countries: a systematic review and meta-analysis. *Lancet* 2019;393(10184):1973-82. doi: 10.1016/S0140-6736(18)32386-9
16. Ameh CA, Weeks AD. The role of instrumental vaginal delivery in low resource settings. *BJOG* 2009;116 Suppl 1:22-5. doi: 10.1111/j.1471-0528.2009.02331.x

For peer review only

17. Geelhoed D, Stokx J, Mariano X, et al. Risk factors for stillbirths in Tete, Mozambique. *Int J Gynaecol Obstet* 2015;130(2):148-52. doi: 10.1016/j.ijgo.2015.03.027
18. World Health Organization. Monitoring emergency obstetric care: a handbook. Geneva, Switzerland; 2009.
19. Paxton A, Bailey PE, Lobis S, Fry D. Measuring progress towards the millennium development goal for maternal health: arguing for an indicator of the health system's ability to respond to obstetric complications. Background paper commissioned by the UN millennium project task force on child health and maternal health; 2004. New York.
20. Ameh C, Msuya S, Hofman J, et al. Status of emergency obstetric care in six developing countries five years before the MDG targets for maternal and newborn health. *PLoS One* 2012;7(12):e49938. doi: 10.1371/journal.pone.0049938
21. Bailey P, Paxton A, Lobis S, et al. The availability of life-saving obstetric services in developing countries: an in-depth look at the signal functions for emergency obstetric care. *Int J Gynaecol Obstet* 2006;93(3):285-91. doi: 10.1016/j.ijgo.2006.01.028
22. Betran AP, Ye J, Moller AB, et al. The Increasing Trend in Caesarean Section Rates: Global, Regional and National Estimates: 1990-2014. *PLoS One* 2016;11(2):e0148343. doi: 10.1371/journal.pone.0148343
23. Bailey PE. The disappearing art of instrumental delivery: time to reverse the trend. *Int J Gynaecol Obstet* 2005;91(1):89-96. doi: 10.1016/j.ijgo.2005.05.016
24. Crossland N, Kingdon C, Balaam MC, et al. Women's, partners' and healthcare providers' views and experiences of assisted vaginal birth: a systematic mixed methods review. *Reprod Health* 2020;17(1):83. doi: 10.1186/s12978-020-00915-w
25. Feeley C, Crossland N, Betran AP, et al. Training and expertise in undertaking assisted vaginal delivery (AVD): a mixed methods systematic review of practitioners views and experiences. *Reprod Health* 2021;18(1):92. doi: 10.1186/s12978-021-01146-3
26. Sorensen BL, Rasch V, Massawe S, et al. Impact of ALSO training on the management of prolonged labor and neonatal care at Kagera Regional Hospital, Tanzania. *Int J Gynaecol Obstet* 2010;111(1):8-12. doi: 10.1016/j.ijgo.2010.04.031
27. Cottrell C, Clausen S, Sullivan D, et al. An Initiative to Increase Resident Performance of Operative Vaginal Deliveries. *J Obstet Gynaecol Can* 2021;43(8):1009-12. doi: 10.1016/j.jogc.2021.01.019
28. Berglund A, Lefevre-Cholay H, Bacci A, et al. Successful implementation of evidence-based routines in Ukrainian maternities. *Acta Obstet Gynecol Scand* 2010;89(2):230-7. doi: 10.3109/00016340903479894
29. Nolens B, Lule J, Namiiro F, et al. Audit of a program to increase the use of vacuum extraction in Mulago Hospital, Uganda. *BMC Pregnancy Childbirth* 2016;16:258. doi: 10.1186/s12884-016-1052-3
30. Dominico S, Bailey PE, Mwakatundu N, et al. Reintroducing vacuum extraction in primary health care facilities: a case study from Tanzania. *BMC Pregnancy Childbirth* 2018;18(1):248. doi: 10.1186/s12884-018-1888-9
31. Geelhoed D, de Deus V, Sitoe M, et al. Improving emergency obstetric care and reversing the underutilisation of vacuum extraction: a qualitative study of implementation in Tete Province, Mozambique. *BMC Pregnancy Childbirth* 2018;18(1):266. doi: 10.1186/s12884-018-1901-3
32. Torloni MR, Siaulys M, Betran AP, Sobhy S, Opiyo N, Daoud S, Thangaratinam S. Interventions to re-introduce or increase operative vaginal deliveries: a systematic review. PROSPERO 2020 CRD42020215224. https://www.crd.york.ac.uk/prospero/display_record.php?ID=CRD42020215224
33. Page MJ, McKenzie JE, Bossuyt PM, et al. The PRISMA 2020 statement: an updated guideline for reporting systematic reviews. *BMJ* 2021;372:n71. doi: 10.1136/bmj.n71
34. Kirkpatrick DL, Kirkpatrick JD. Implementing the four levels: a practical guide for effective evaluation of training programs. San Francisco, US: Berrett-Koehler Publishers, 2007.
35. Cochrane Effective Practice and Organisation of Care (EPOC). EPOC Resources for review authors, 2017. https://epoc.cochrane.org/sites/epoc.cochrane.org/files/public/uploads/Resources-for-authors2017/suggested_risk_of_bias_criteria_for_epoc_reviews.pdf Accessed on 20, May, 2021).

For peer review only

36. Sterne JA, Hernan MA, Reeves BC, et al. ROBINS-I: a tool for assessing risk of bias in non-randomised studies of interventions. *BMJ* 2016;355:i4919. doi: 10.1136/bmj.i4919
37. Fantom NJ, Serajuddin U. The World Bank's classification of countries by income (English). Policy Research working paper,no. WPS 7528 Washington, D.C. : World Bank Group. http://documents.worldbank.org/curated/en/408581467988942234/The-World-Banks-classification-of-countries-by-income.
38. Bardos J, Loudon H, Rekawek P, et al. Association Between Senior Obstetrician Supervision of Resident Deliveries and Mode of Delivery. *Obstet Gynecol* 2017;129(3):486-90. doi: 10.1097/AOG.0000000000001910
39. Becker DA, Blanchard CT, Szychowski JM, et al. Resident Operative Vaginal Delivery Volume after Educational Curriculum Implementation. *Am J Perinatol* 2020;37(13):1296-300. doi: 10.1055/s-0040-1710543
40. Dumont A, Fournier P, Abrahamowicz M, et al. Quality of care, risk management, and technology in obstetrics to reduce hospital-based maternal mortality in Senegal and Mali (QUARITE): a cluster-randomised trial. *Lancet* 2013;382(9887):146-57. doi: 10.1016/S0140-6736(13)60593-0
41. Gulmezoglu AM, Langer A, Piaggio G, et al. Cluster randomised trial of an active, multifaceted educational intervention based on the WHO Reproductive Health Library to improve obstetric practices. *BJOG* 2007;114(1):16-23. doi: 10.1111/j.1471-0528.2006.01091.x
42. Sequeira Dmello B, Sellah Z, Magembe G, et al. Learning from changes concurrent with implementing a complex and dynamic intervention to improve urban maternal and perinatal health in Dar es Salaam, Tanzania, 2011-2019. *BMJ Glob Health* 2021;6(1) doi: 10.1136/bmjgh-2020-004022
43. Skinner S, Davies-Tuck M, Wallace E, et al. Perinatal and Maternal Outcomes After Training Residents in Forceps Before Vacuum Instrumental Birth. *Obstet Gynecol* 2017;130(1):151-58. doi: 10.1097/AOG.0000000000002097
44. Solt I, Jackson S, Moore T, et al. Teaching forceps: the impact of proactive faculty. *Am J Obstet Gynecol* 2011;204(5):448 e1-4. doi: 10.1016/j.ajog.2010.12.056
45. Takeda J, Ohashi R. Effect of introducing forceps delivery at a local primary clinic. *Hypertension Research in Pregnancy* 2018;6(1):30-31. doi: 10.14390/jsshp.HRP2018-004
46. Mogilevkina I, Gurianov V, Lindmark G. Effectiveness of emergency obstetric care training at the regional level in Ukraine: a non-randomized controlled trial. *BMC Pregnancy Childbirth* 2022;22(1):145. doi: 10.1186/s12884-022-04458-9
47. Ameh CA. The effectiveness of emergency obstetric care training in Kenya. PhD thesis. University of Liverpool, 2014. https://livrepository.liverpool.ac.uk/2008539/3/AmehCha_Nov2014_2008539.pdf.
48. Ghag K, Bahl R, Winter C, et al. Key components influencing the sustainability of a multi-professional obstetric emergencies training programme in a middle-income setting: a qualitative study. *BMC Health Serv Res* 2021;21(1):384. doi: 10.1186/s12913-021-06385-5
49. Merriel A, Ficquet J, Barnard K, et al. The effects of interactive training of healthcare providers on the management of life-threatening emergencies in hospital. *Cochrane Database Syst Rev* 2019;9:CD012177. doi: 10.1002/14651858.CD012177.pub2
50. Altieri E, Grove J, Davies OL, et al. Harnessing the power of behavioural science to improve health. *Bull World Health Organ* 2021;99(11):754-54A. doi: 10.2471/BLT.21.287375
51. Michie S, van Stralen MM, West R. The behaviour change wheel: a new method for characterising and designing behaviour change interventions. *Implement Sci* 2011;6:42. doi: 10.1186/1748-5908-6-42
52. Adam T, de Savigny D. Systems thinking for strengthening health systems in LMICs: need for a paradigm shift. *Health Policy Plan* 2012;27 Suppl 4:iv1-3. doi: 10.1093/heapol/czs084
53. Long Q, Allanson ER, Pontre J, et al. Onsite midwife-led birth units (OMBUs) for care around the time of childbirth: a systematic review. *BMJ Glob Health* 2016;1(2):e000096. doi: 10.1136/bmjgh-2016-000096
54. Doyle C, Lennox L, Bell D. A systematic review of evidence on the links between patient experience and clinical safety and effectiveness. *BMJ Open* 2013;3(1) doi: 10.1136/bmjopen-2012-001570

For peer review only

55. Downe S, Finlayson K, Oladapo OT, et al. What matters to women during childbirth: A systematic qualitative review. *PLoS One* 2018;13(4):e0194906. doi: 10.1371/journal.pone.0194906
56. Hotton EJ, Alvarez M, Lenguerrand E, et al. The Odon Device for assisted vaginal birth: a feasibility study to investigate safety and efficacy-The ASSIST II study. *Pilot Feasibility Stud* 2021;7(1):72. doi: 10.1186/s40814-021-00814-2
57. Thomas J, O'Mara-Eves A, Brunton G. Using qualitative comparative analysis (QCA) in systematic reviews of complex interventions: a worked example. *Syst Rev* 2014;3:67. doi: 10.1186/2046-4053-3-67
58. Chatterley C, Javernick-Will A, Linden KG, et al. A qualitative comparative analysis of well-managed school sanitation in Bangladesh. *BMC Public Health* 2014;14:6. doi: 10.1186/1471-2458-14-6
59. Craig P, Dieppe P, Macintyre S, et al. Developing and evaluating complex interventions: the new Medical Research Council guidance. *BMJ* 2008;337:a1655. doi: 10.1136/bmj.a1655

Figure legend:

Figure 1. Flowchart of process of study identification and inclusion

List of supplementary files

Supplementary file 1: Search strategy

Supplementary file 2: Studies excluded

Supplementary file 3: Study details

Supplementary file 4: Study quality assessment

Supplementary file 5: Baseline rates

Supplementary file 6: Interventions details

Supplementary file 7: Outcomes reported in the studies

Supplementary file 8: Summary of effects

For peer review only

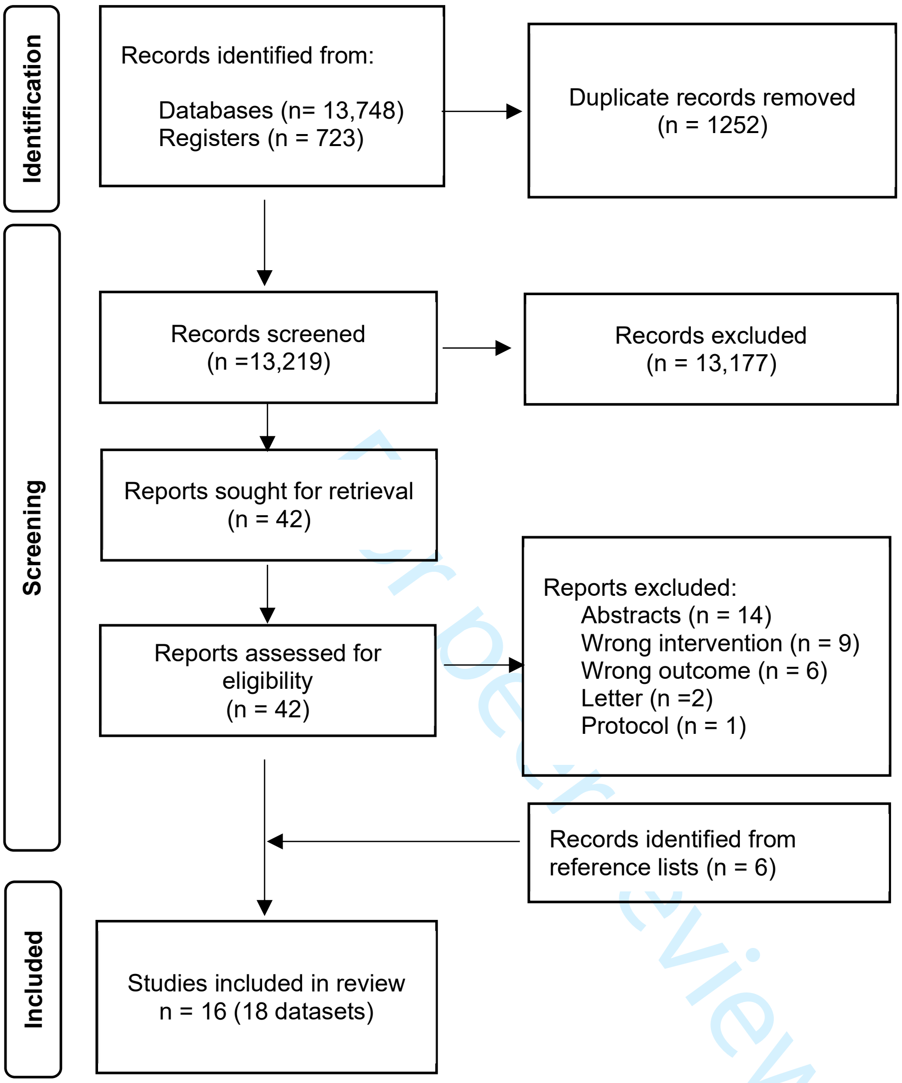
Identification
Records identified from:
Databases (n= 13,748)
Registers (n = 723)
Duplicate records removed
(n = 1252)
Screening
Records screened
(n =13,219)
Records excluded
(n = 13,177)
Reports sought for retrieval
(n = 42)
Reports assessed for eligibility
(n = 42)
Reports excluded:
Abstracts (n = 14)
Wrong intervention (n = 9)
Wrong outcome (n = 6)
Letter (n =2)
Protocol (n = 1)
Records identified from reference lists (n = 6)
Included
Studies included in review
n = 16 (18 datasets)

Supplementary file 1. Search strategy

Initial searches December 31, 2019. Updated on September 1, 2021

MEDLINE / PubMed

Searches
#1 “operative vaginal delivery” OR “operative vaginal deliver*” OR OVD [ti]
#2 “assisted vaginal delivery” OR “assisted vaginal deliver*” OR AVD [ti]
#3 “assisted vaginal birth” OR AVB [ti]
#4 “instrumental delivery” OR “instrumental deliver*” OR “instrumental vaginal delivery” OR “instrumental vaginal deliver*” [ti]
#5 “kiwi delivery” OR “kiwi deliver*” [ti]
#6 “ventouse delivery” OR “ventouse deliver*” OR “ventouse assisted delivery” OR “ventouse assisted deliver*” [ti]
#7 “vacuum delivery” OR “vacuum deliver*” OR “vacuum assisted delivery” OR “vacuum assisted deliver*” [ti]
#8 “forceps delivery” OR “forceps deliver*” OR “forceps assisted delivery” OR “forceps assisted deliver*” [ti]
#9 “obstetric forceps” OR “obstetrical forceps” [ti]
#10 ventouse OR “vacuum extraction” OR “vacuum extractor” [ti]
#11 #1 OR #2 OR #3 OR #4 OR #5 OR #6 OR #7 OR #8 OR #9 OR #10

EMBASE

Searches
#1 “operative vaginal delivery” [exp]
#2 “operative vaginal delivery” OR “operative vaginal deliver*” OR OVD [ti]
#3 “assisted vaginal delivery” [exp]
#4 “assisted vaginal delivery” OR “assisted vaginal deliver*” OR AVD [ti]
#5 “assisted vaginal birth” OR AVB [ti]
#6 “instrumental delivery” [exp] ('forceps delivery'/exp OR 'vacuum extraction'/exp)
#7 “instrumental delivery” OR “instrumental deliver*” OR “instrumental vaginal delivery” OR “instrumental vaginal deliver*” [ti]
#8 “kiwi delivery” OR “kiwi deliver*” [ti]
#9 “ventouse delivery” OR “ventouse deliver*” OR “ventouse assisted delivery” OR “ventouse assisted deliver*” [ti]
#10 “obstetric vacuum delivery kit” [exp]
#11 “obstetric vacuum delivery kit” OR “vacuum delivery” OR “vacuum deliver*” OR “vacuum assisted delivery” OR “vacuum assisted deliver*” [ti]
#12 “forceps delivery” [exp]
#13 “forceps delivery” OR “forceps deliver*” OR “forceps assisted delivery” OR “forceps assisted deliver*” [ti]
#14 “obstetric forceps” [exp]
#15 “obstetric forceps” OR “obstetrical forceps” [ti]
#16 “vacuum extraction” OR “vacuum extractor” [exp]
#17 ventouse OR “vacuum extraction” OR “vacuum extractor” [ti]
#18 #1 OR #2 OR #3 OR #4 OR #5 OR #6 OR #7 OR #8 OR #9 OR #10 OR #11 OR #12 OR #13 OR #14 OR #15 OR #16 OR #17

For peer review only - http://bmjopen.bmj.com/site/about/guidelines.xhtml

CINAHL

Searches
#1 “vaginal birth” [SU]<br>#2 “operative vaginal delivery” OR “operative vaginal deliver*” OR OVD [ti]<br>#3 “assisted vaginal delivery” OR “assisted vaginal deliver*” OR AVD [ti]<br>#4 “assisted vaginal birth” OR AVB [ti]<br>#5 “instrumental delivery” OR “instrumental deliver*” OR “instrumental vaginal delivery” OR “instrumental vaginal deliver*” [ti]<br>#6 “kiwi delivery” OR “kiwi deliver*” [ti]<br>#7 “ventouse delivery” OR “ventouse deliver*” OR “ventouse assisted delivery” OR “ventouse assisted deliver*” [ti]<br>#8 “vacuum delivery” OR “vacuum deliver*” OR “vacuum assisted delivery” OR “vacuum assisted deliver*” [ti]<br>#9 “forceps delivery” OR “forceps deliver*” OR “forceps assisted delivery” OR “forceps assisted deliver*” [ti]<br>#10 “obstetrical forceps” [SU]<br>#11 “obstetric forceps” OR “obstetrical forceps” [ti]<br>#12 “vacuum extraction, obstetrical” [SU]<br>#13 ventouse OR “vacuum extraction” OR “vacuum extractor” [ti]<br>#14 #1 OR #2 OR #3 OR #4 OR #5 OR #6 OR #7 OR #8 OR #9 OR #10 OR #11 OR #12 OR #13

LILACS

Searches
#1 “operative vaginal delivery” OR “operative vaginal deliver*” OR OVD [ti]<br>#2 “assisted vaginal delivery” OR “assisted vaginal deliver*” OR AVD [ti]<br>#3 “assisted vaginal birth” OR AVB [ti]<br>#4 “instrumental delivery” OR “instrumental deliver*” [ti]<br>#5 “instrumental vaginal delivery” OR “instrumental vaginal deliver*” [ti]<br>#6 “kiwi delivery” OR “kiwi deliver*” [ti]<br>#7 “ventouse delivery” OR “ventouse deliver*” [ti]<br>#8 “ventouse assisted delivery” OR “ventouse assisted deliver*” [ti]<br>#9 “vacuum delivery” OR “vacuum deliver*” [ti]<br>#10 “vacuum assisted delivery” OR “vacuum assisted deliver*” [ti]<br>#11 “forceps delivery” OR “forceps deliver*” [ti]<br>#12 “forceps assisted delivery” OR “forceps assisted deliver*” [ti]<br>#13 “obstetric forceps” OR “obstetrical forceps” [ti]<br>#14 ventouse OR “vacuum extraction” OR “vacuum extractor” [ti]

For peer review only - http://bmjopen.bmj.com/site/about/guidelines.xhtml

SCOPUS

Searches
#1 “operative vaginal delivery” OR “operative vaginal deliver*” OR OVD [ti]
#2 “assisted vaginal delivery” OR “assisted vaginal deliver*” OR AVD [ti]
#3 “assisted vaginal birth” OR AVB [ti]
#4 “instrumental delivery” OR “instrumental deliver*” OR “instrumental vaginal delivery” OR “instrumental vaginal deliver*” [ti]
#5 “kiwi delivery” OR “kiwi deliver*” [ti]
#6 “ventouse delivery” OR “ventouse deliver*” OR “ventouse assisted delivery” OR “ventouse assisted deliver*” [ti]
#7 “vacuum delivery” OR “vacuum deliver*” OR “vacuum assisted delivery” OR “vacuum assisted deliver*” [ti]
#8 “forceps delivery” OR “forceps deliver*” OR “forceps assisted delivery” OR “forceps assisted deliver*” [ti]
#9 “obstetric forceps” OR “obstetrical forceps” [ti]
#10 ventouse OR “vacuum extraction” OR “vacuum extractor” [ti]
#11 #1 OR #2 OR #3 OR #4 OR #5 OR #6 OR #7 OR #8 OR #9 OR #10

Cochrane Library

Searches
#1 “operative vaginal delivery” OR “operative vaginal deliver*” OR OVD [ti]
#2 “assisted vaginal delivery” OR “assisted vaginal deliver*” OR AVD [ti]
#3 “assisted vaginal birth” OR AVB [ti]
#4 “instrumental delivery” OR “instrumental deliver*” OR “instrumental vaginal delivery” OR “instrumental vaginal deliver*” [ti]
#5 “kiwi delivery” OR “kiwi deliver*” [ti]
#6 “ventouse delivery” OR “ventouse deliver*” OR “ventouse assisted delivery” OR “ventouse assisted deliver*” [ti]
#7 “vacuum delivery” OR “vacuum deliver*” OR “vacuum assisted delivery” OR “vacuum assisted deliver*”
#8 “forceps delivery” OR “forceps deliver*” OR “forceps assisted delivery” OR “forceps assisted deliver*” [ti]
#9 “obstetrical forceps” [MeSH]
#10 “obstetric forceps” OR “obstetrical forceps” [ti]
#11 “vacuum extraction, obstetrical” [MeSH]
#12 ventouse OR “vacuum extraction” OR “vacuum extractor” [ti]
#13 #1 OR #2 OR #3 OR #4 OR #5 OR #6 OR #7 OR #8 OR #9 OR #10 OR #11 OR #12

For peer review only

For peer review only - http://bmjopen.bmj.com/site/about/guidelines.xhtml

WHO Library

Searches
#1 “operative vaginal delivery” OR “operative vaginal deliver*” OR OVD [ti]<br>#2 “assisted vaginal delivery” OR “assisted vaginal deliver*” OR AVD [ti]<br>#3 “assisted vaginal birth” OR AVB [ti]<br>#4 “instrumental delivery” OR “instrumental deliver*” [ti]<br>#5 “instrumental vaginal delivery” OR “instrumental vaginal deliver*” [ti]<br>#6 “kiwi delivery” OR “kiwi deliver*” [ti]<br>#7 “ventouse delivery” OR “ventouse deliver*” [ti]<br>#8 “ventouse assisted delivery” OR “ventouse assisted deliver*” [ti]<br>#9 “vacuum delivery” OR “vacuum deliver*” [ti]<br>#10 “vacuum assisted delivery” OR “vacuum assisted deliver*” [ti]<br>#11 “forceps delivery” OR “forceps deliver*” [ti]<br>#12 “forceps assisted delivery” OR “forceps assisted deliver*” [ti]<br>#13 “obstetric forceps” OR “obstetrical forceps” [ti]<br>#14 ventouse OR “vacuum extraction” OR “vacuum extractor” [ti]<br>#15 #1 OR #2 OR #3 OR #4 OR #5 OR #6 OR #7 OR #8 OR #9 OR #10 OR #11 OR #12 OR #13 OR #14

Web of science

Searches
#1 “operative vaginal delivery” OR “operative vaginal deliver*” OR OVD [ti]<br>#2 “assisted vaginal delivery” OR “assisted vaginal deliver*” OR AVD [ti]<br>#3 “assisted vaginal birth” OR AVB [ti]<br>#4 “instrumental delivery” OR “instrumental deliver*” OR “instrumental vaginal delivery” OR “instrumental vaginal deliver*” [ti]<br>#5 “kiwi delivery” OR “kiwi deliver*” [ti]<br>#6 “ventouse delivery” OR “ventouse deliver*” OR “ventouse assisted delivery” OR “ventouse assisted deliver*” [ti]<br>#7 “vacuum delivery” OR “vacuum deliver*” OR “vacuum assisted delivery” OR “vacuum assisted deliver*” [ti]<br>#8 “forceps delivery” OR “forceps deliver*” OR “forceps assisted delivery” OR “forceps assisted deliver*” [ti]<br>#9 “obstetric forceps” OR “obstetrical forceps” [ti]<br>#10 ventouse OR “vacuum extraction” OR “vacuum extractor” [ti]<br>#11 #1 OR #2 OR #3 OR #4 OR #5 OR #6 OR #7 OR #8 OR #9 OR #10

For peer review only - http://bmjopen.bmj.com/site/about/guidelines.xhtml

MEDLINE/PubMed- Training programs

Search terms
(RCOG Operative vaginal Birth Simulation Training OR ROBuST) OR<br>(Advanced Life Support in Obstetrics OR ALSO) OR<br>(Programa de Rescate Obstétrico y Neonatal: Tratamiento Óptimo y Oportuno OR PRONTO) OR<br>(Essential Steps in the Management of Obstetric Emergencies OR ESMOE) OR<br>(assisted vaginal birth OR operative vaginal birth OR instrumental vaginal delivery OR forceps delivery OR vacuum delivery)

For peer review only

For peer review only - http://bmjopen.bmj.com/site/about/guidelines.xhtml

Supplementary file 2. Studies excluded

List of 32 studies identified through electronic search and excluded after full-text reading

Study reference	Reason for exclusion	Category for exclusion
Becker, D. A.; Blanchard, C. T.; Szychowski, J. M.; Rogers, S. L.; Brumfield, C. G.; Subramaniam, A. 743: The effect of an operative vaginal delivery curriculum for OBGYN residents American Journal of Obstetrics and Gynecology 2019;220(1):S488	Conference abstract of study later published and included (Becker 2020)	Abstract
Beneru, D.; Mcgee, T.; Jenkins, G. Caesarean sections at full dilatation before and after mandatory consultant presence. Australian and New Zealand Journal of Obstetrics and Gynaecology 2021;61(SUPPL 1):6	Conference abstract only	Abstract
Caughey, A.; Cheng, Y. W.; Zlatnik, M.; Nguy, M.; Nguyen, L.; Thiet, M. P. Perineal lacerations and operative vaginal delivery: Results from a quality improvement program American Journal of Obstetrics and Gynecology Dec 2007;197(6):S79-S79	Conference abstract only	Abstract
Coleman, L.; Basude, S. A simple program of focused education and simulation training to reduce the rate of obstetric anal sphincter injury at forceps delivery. BJOG. Apr 2015;122():78-78	Conference abstract only	Abstract
Fox, Nathan S.; Bardos, Jonah; Loudon, Holly; Rekawek, Patricia; Friedman, Frederick; Brodman, Michael. 523: Adding senior obstetrician supervision increases the forceps delivery rate and decreases the cesarean delivery rate for residents. American Journal of Obstetrics and Gynecology 2017; 216(1S):S309	Conference abstract only	Abstract
Halawani, M.; Webster, S.; Soliman, E.; El-Nouri, A. Reintroduction of operative vaginal delivery at a large tertiary referral hospital in Cairo, Egypt. International Journal of Gynecology and Obstetrics 2018;143:279	Conference abstract only	Abstract
Hodges, R.; Skinner, S.; Wallace, E.; Davies, M. Perinatal and maternal outcomes after training obstetric residents in forceps before vacuum operative birth. American Journal of Obstetrics and Gynecology 2016;214(1):S270-S271	Conference abstract of study later published and included (Skinner 2017)	Abstract
Jovic, E.; Sethna, F. Does consultant attendance for a second-stage delivery in theatre make a difference? Australian and New Zealand Journal of Obstetrics and Gynaecology 2021;61(SUPPL 1):71-72	Conference abstract only	Abstract
Muller, B.; Gilreath, N.; Wineland, R.; Sullivan, S.; Finneran, M.M. Impact of contemporary labor curves on primary cesarean delivery at a single institution American Journal of Obstetrics and Gynecology 2021;224(2):S386	Conference abstract only	Abstract
Negi, Masaru MD; Espinal, Mariana MD; Holveck, Beth RN; Mehlhaff, Krista MD; Cron, Julia MD; Pettker, Christian Michael MD Improvement of Operative Vaginal Delivery Training in Residency: A Single Institution Experience [17G], Obstetrics & Gynecology: May 2020 - Volume 135 - Issue - p 75S	Conference abstract only	Abstract
Russell, M.; Esen, U. Achieving safety and success of operative vaginal deliveries at a district teaching hospital - A triumph of audit and a targeted practical teaching workshop. International Journal of Gynecology and Obstetrics 2009;107(S2):S548	Conference abstract only	Abstract

For peer review only

Stephens-Hennessy, Beth M. Implementation of the California Maternal Quality Care Collaborative Toolkit to Support Vaginal Birth and Reduce Primary Cesarean Births JOGNN: Journal of Obstetric, Gynecologic & Neonatal Nursing 2018;47(3):S51-S52	Conference abstract only	Abstract
Wilkinson, M; Mumtaz, H; Nassar, M; Gopal, G. Does consultant presence at trials of operative vaginal delivery increase success? BJOG- 2021;128(S2):152	Conference abstract only	Abstract
Zaid, R. Z.; Yoong, W.; Lhodi, W.; Hirsch, M.; Anderson, K.; Balghari, Z.; Aziz, A.; Relph, S.; Sivashanmugarajan, V.; Govind, A. Delivering a modular, concise training package for instrumental vaginal deliveries. BJOG 2013;120(S2):594	Conference abstract only	Abstract
Dietz, Hans Peter; Ka Lai, Shek; Callaghan, Sascha; Shek, Ka Lai. Perinatal and Maternal Outcomes After Training Residents in Forceps Before Vacuum Instrumental Birth. Obstetrics & Gynecology 2017;130(4):910-910	Letter commenting study by Skinner 2017	Letter
Spencer, C.; Murphy, D.; Bewley, S. Caesarean delivery in the second stage of labour: better training in instrumental delivery may reduce rates. BMJ 2006;333(7569):613-614	Letter	Letter
van Tetering AAC, van Meurs A, Ntuyo P, van der Hout-van der Jagt MB, Mulders LGM, Nolens B, Namagambe I, Nakimuli A, Byamugisha J, Oei SG. Study protocol training for life: a stepped wedge cluster randomized trial about emergency obstetric simulation-based training in a low-income country. BMC Pregnancy Childbirth. 2020 Jul 28;20(1):429	Study protocol	Protocol
Ayala NK, Schlichting LE, Kole MB, Clark MA, Vivier PM, Viner-Brown SI, Werner EF. Operative vaginal delivery and third grade educational outcomes.Am J Obstet Gynecol MFM. 2020 Nov;2(4):100221.	Not a training or QI initiative to increase AVB	Wrong Intervention
Chang, Xavier; Chedraui, Peter; Ross, Michael G.; Hidalgo, Luis; Penafiel, Jaime Vacuum assisted delivery in Ecuador for prolonged second stage of labor: maternal-neonatal outcome The journal of maternal-fetal & neonatal medicine. 2007 2007;20(5):381-4	Not a training or QI initiative	Wrong Intervention
Chen CC, Lee JF. Effectiveness of the doula program in Northern Taiwan. Tzu Chi Med J. 2020 Apr 1;32(4):373-379.	Not a training or QI initiative to increase AVB	Wrong Intervention
Davison, Margaret Anne; Murray, Sarah; Whitaker, Lucy; Rendall, Lesley; Gammie, Nicky; Magowan, Brian. Comparison of instrumental vaginal births by assisted birth practitioner midwives and medical practitioners. British Journal of Midwifery 2014;22(10):700-705	Not a training or QI initiative to increase AVB	Wrong Intervention
Ghi T, Rizzo G; EGEO Group. The use of a hybrid mannequin for the modern high-fidelity simulation in the labor ward: the Italian experience of the Ecografia Gestione Emergenze Ostetriche (EGEO) group. Am J Obstet Gynecol. 2020 Jan;222(1):41-47.	Not a training or QI initiative to increase AVB	Wrong Intervention
Hildebrand E, Nelson M, Blomberg M. Long-term effects of the nine-item list intervention on obstetric and neonatal outcomes in Robson group 1 - A time series study. Acta Obstet Gynecol Scand. 2021 Jan;100(1):154-161.	Not a training or QI initiative to increase AVB	Wrong Intervention
Hinkson L, Henrich W, Tutschek B. Intrapartum ultrasound during rotational forceps delivery: a novel tool for safety, quality control, and teaching. Am J Obstet Gynecol. 2021 Jan;224(1):93.e1-93.e7.	Not a training or QI initiative to increase AVB	Wrong Intervention

For peer review only - http://bmjopen.bmj.com/site/about/guidelines.xhtml

Romero S, Pettersson K, Yousaf K, Westgren M, Ajne G. Perinatal outcome after vacuum assisted delivery with digital feedback on traction force; a randomised controlled study. BMC Pregnancy Childbirth. 2021 Feb 26;21(1):165. doi: 10.1186/s12884-021-03604-z. PMID: 33637058; PMCID: PMC7913459.	Not a training or QI initiative to increase AVB	Wrong Intervention
Waller-Wise R, Lewis S, Williams B. A Quality Improvement Project Utilizing a Clinical Practice Guideline in Women During Second-Stage Labor. J Perinat Educ. 2020 Apr 1;29(2):72-82. doi: 10.1891/J-PE-D-19-00014. PMID: 32308356; PMCID: PMC7159796.	Not a training or QI initiative to increase AVB	Wrong Intervention
Cheong, Y. C.; Abdullahi, H.; Lashen, H.; Fairlie, F. M. Can formal education and training improve the outcome of instrumental delivery? European Journal of Obstetrics and Gynecology and Reproductive Biology 2004;113(2):139-144	Increase of AVB use was not one of the Objectives or cited as one of the Outcomes in Methods	Wrong Outcome
Chikazawa, K.; Takagi, K.; Takahashi, H.; Akashi, K.; Nakamura, E.; Samejima, K.; Ushijima, J.; Horiuchi, I. Introduction of forceps delivery education for residents at a single perinatal institution. Hypertension Research in Pregnancy 2016;4(2):102-105	No data on AVB use before x after intervention	Wrong Outcome
Coste Mazeau, P; Boukeffa, N; Ticaud Boileau, N; Huet, S; Traverse, M; Eyraud, JL; Laguerre, A; Catalan, C; Riedl, C Evaluation of Suzor forceps training by studying obstetric anal sphincter injuries: a retrospective study. BMC Pregnancy Childbirth 20, 674 (2020)	No data on AVB use before x after intervention	Wrong Outcome
Dolo O, Clack A, Gibson H, Lewis N, Southall DP. Training of midwives in advanced obstetrics in Liberia. Bull World Health Organ. 2016 May 1;94(5):383-7.	No data on AVB use before x after intervention	Wrong Outcome
Egami N, Muta R, Anami A, Koga H. Impact of clinical practice guidelines for vacuum-assisted delivery on maternal and neonatal outcomes in Japan: A single-center observational study. J Obstet Gynaecol Res. 2021 Jan;47(1):167-173	No data on AVB use before x after intervention (assessed only success rates).	Wrong Outcome
van Tetering AAC, Segers MHM, Ntuyo P, Namagambe I, van der Hout-van der Jagt MB, Byamugisha JK, Oei SG. Evaluating the Instructional Design and Effect on Knowledge, Teamwork, and Skills of Technology-Enhanced Simulation-Based Training in Obstetrics in Uganda: Stepped-Wedge Cluster Randomized Trial. JMIR Med Educ. 2021 Feb 5;7(1):e17277	Present only Kirpatrick 1 and 2 outcomes	Wrong outcome

For peer review only

Supplementary file 3. Details of 16 studies on interventions to increase AVB use

Author	Study design / Year study conducted / Level of significance / Adjustments	Country / Setting	Population / Number of deliveries	Intervention strategy / Frequency and duration	Effect of intervention (Kirkpatrick levels 3 or 4)
Ameh 2014	Cluster-randomised trial (stepped wedge)<br><br>2009-2011<br><br>P < 0.05<br><br>Adjustment for clustering	Kenya<br><br>2 "level 5" hospitals and 8 regional hospitals<br><br>All health care facilities involved were provincial "referral hospitals"	All women (N=16,794 at baseline, N=17,404 intervention period Total N= 34,198)	Multifaceted intervention comprising training in emergency obstetric and neonatal care (EmONC), provision of EmOC equipment (including reusable Kiwi omnicups) and local supportive supervision.<br><br>Duration of training: 3 days	• Significant increase in vacuum extraction (from 0.2% to 1% at 12 months) P< 0.001<br>• No significant change in overall CS (baseline 21.5% vs. post-intervention 21.5%)<br>• No significant change in direct obstetric case fatality rate (from 3% to 2%)<br>• Significant decrease in NICU admission due to birth asphyxia (48% mean reduction at 12 months, from 7.6% to 4%) P = 0.03<br>• Significant decrease in stillbirth rate at 12 months (35% mean reduction at 12 months, from 5% to 3%) P = 0.03<br>• No significant change in fresh stillbirth rate (from 2.1% to 1.9%)
Bardos 2017	Before and after study<br><br>2011-2015<br><br>P< 0.05<br>Confounding variables included in multivariable logistic regression: maternal age ≥ 35, maternal obesity (BMI≥ 35 Kg/m2 at delivery), induction of labor, parity, and prior cesarean delivery. The last two variables were not included in the analysis of nulliparous patients only.	USA<br><br>1 Teaching hospital	Term, singleton, cephalic births cared by residents (N=1919 at baseline, N=3282 intervention period, Total N=5201)	Senior obstetrician (>20 years experience) teaching and supervising residents on forceps deliveries during the day time.<br><br>Frequency and duration not stated.	• Significant increase in forceps deliveries (0.6% to 2.6%, aOR 8.44, 95% CI 3.1 to 23.1)<br>• No significant change in vacuum deliveries (3.3% to 3.3%)<br>• Significant decrease in overall CS (27.3% to 24.5%, aOR 0.68, 95% CI 0.55 to 0.83)<br>• No significant change in:<br>-3rd-4th degree tears (1.4% to 2.0%)<br>-5 min Apgar score < 7 (0.3% to 0.5%)<br>The increase in forceps deliveries and the decrease in CS were seen only in daytime hours (7 AM to 7 PM), that is, the shift that was covered by senior obstetricians

For peer review only

**Author**	**Study design / Year study conducted/ Level of significance / Adjustments**	**Country / Setting**	**Population / Number of deliveries**	**Intervention strategy / Frequency and duration**	**Effect of intervention** (Kirkpatrick levels 3 or 4)
Becker 2020	Retrospective cohort study<br><br>2011-2018<br><br>P< 0.05<br><br>No adjustments	USA<br><br>1 Teaching hospital	All women with births after 20 weeks gestation (N=20,471 at baseline, N=6106 intervention period, Total N=26,577)	Twice-yearly AVB residency curriculum consisting of didactics (pretest and posttest of basic AVB knowledge, history, indications, safe use, risks of forceps and vacuum) and hands-on simulation of AVB.<br><br>Frequency and duration: One 2-hour workshop offered every 6 months	• Significant increase in forceps use, mean (SD), pre-1.2% (0.6) vs. post 2.0% (0.9), P = 0.027<br>• Significant decrease in vacuum use, mean (SD), pre-2.0% (0.5) vs. post-1.2% (0.3), P < 0.001<br>• No significant change in overall AVB, mean (SD), pre-3.2% (0.8) vs. post-3.1% (1.0)<br>• Increase in overall CS rates, from 27.9%-30.6% in pre-intervention years to 33.5%-33.9% in post-intervention years (no statistical analyses)

For peer review only

Author	Study design / Year study conducted/ Level of significance / Adjustments	Country / Setting	Population / Number of deliveries	Intervention strategy / Frequency and duration	Effect of intervention (Kirkpatrick levels 3 or 4)
Berglund 2010	Before and after study<br><br>2003-2006<br><br>P< 0.05<br><br>No adjustments	Ukraine<br><br>3 Maternity hospitals (Donetsk, Lutsk, Lviv)	All women (in the 3 sites combined: N=1696 at baseline, N= 6710 intervention period, Total N= 8406)	Development of obstetrical and neonatal protocols, staff training on Effective Perinatal Care (EPC), continuous monitoring and evaluation (with feedback to staff), revision of university curricula to include evidence-based perinatal care, provision of equipment, resuscitation and neonatal care.<br><br>Frequency and duration:<br>Initial (EPC) training: two weeks<br>Follow up EPC training: three days	• Significant decrease in overall AVB in one maternity:<br>Donetsk: from 3.7% to 0% P<0.0001<br>• No change in overall AVB in the other two maternities:<br>Lutsk: from 2.0% to 2.0%<br>Lviv: from 0% to 0%<br>• Significant decrease in CS in two maternities:<br>Donetsk: from 30% to 18.4% P<0.0001<br>Lutsk: from 33% to 12.7% P<0.0001<br>• No change in CS in one maternity:<br>Lviv: from 21.9% to 16.9%<br>• Significant decrease in episiotomies:<br>Donetsk: from 36.8%to 4.0% P<0.0001<br>Lutsk: from 19.4% to 5.7% P<0.0001<br>Lviv: 22.2% to 7.0% P<0.0001<br>• No effect on early neonatal mortality:<br>Donetsk: from 13.6% to 9.5%<br>Lutsk: from 4.6% to 3.0%<br>Lviv: from 6.4% to 0%<br>• Significant decrease in babies needing resuscitation:<br>Donetsk: from 13.1% to 7.4% P = 0.0025<br>Lutsk: from 2.4% to 0.9% P<0.0001<br>Lviv: 4.5% to 1.1% P=0.0003<br>• Significant decrease in NICU admission in two maternities:<br>Lutsk: from 7.3% to 4.4% P= 0.0115<br>Lviv: from 6.4% to 2.4% P = 0.0015<br>• No significant change in NICU admission in one maternity:<br>Donetsk: from 11.2% to 9.4%<br>• Significant decrease in the proportion of hypothermic infants:<br>Donetsk: from 59.5% to 0.6% P<0.0001<br>Lutsk: from 85.8% to 0.3% P<0.0001<br>Lviv: from 77% to 0.7% P<0.0001

For peer review only

4

Author	Study design / Year study conducted/ Level of significance / Adjustments	Country / Setting	Population / Number of deliveries	Intervention strategy / Frequency and duration	Effect of intervention (Kirkpatrick levels 3 or 4)
Cottrell 2021	Cohort study<br><br>2013-2017<br><br>P< 0.05<br><br>No adjustments	USA<br><br>1 Teaching hospital	Women with prolonged second stage labour (N=2720 at baseline, N= 2800 intervention period Total N=5520)	On-the-job, hands-on AVB training of residents and supervision by attending physicians.<br><br>Frequency and duration not stated.	• Significant increase in forceps deliveries, from 1.8% to 4.0%, P < 0.001<br>• Significant decrease in vacuum deliveries, from 8.7% to 6.5%, P = 0.002.<br>• No significant change in overall AVB, from 10.5% to 10.5%<br>• Increase in overall CS rate from 30.4% to 32.1%, no statistical analysis<br>• 3rd degree laceration: no change in forceps (post vs. pre, RR 0.5, 95% CI 0.24-1.21) and vacuum groups (post vs. pre, RR 0.8, 95% CI 0.37−1.55)<br>• 4th degree laceration: no change in vacuum (post vs. pre, RR 1.0, 95% CI 0.28-3.83); significant decrease in forceps group (post vs. pre, RR 0.1, 95% CI 0.01-0.73)<br>• Overall maternal complication rates: significant decrease in forceps group (post vs. pre, RR 0.40, 95% CI 0.19-0.75); no change in vacuum group (post vs. pre, RR 0.8, 95% CI 0.44-1.51)<br>• Facial laceration: no change in forceps (post vs. pre, RR 0.9, 95% CI 0.08-9.42) and vacuum groups (post vs. pre, RR 0.8, 95% CI 0.19-3.23)<br>• Scalp injury: no change in forceps (post vs. pre, Not estimable) and vacuum groups (post vs. pre, RR 0.5, 95% CI 0.15-1.46)<br>• Cephalohematoma: no change in forceps (post vs. pre, Not estimable) and vacuum groups (post vs. pre, RR 0.8, 95% CI 0.22-2.51)<br>• Overall neonatal complications: no change in forceps (post vs. pre, RR 0.4, 95% CI 0.09-2.09), vacuum groups (RR 0.6, 95% CI 0.31−1.25) and overall (post vs. pre, RR 0.4, 95% CI 0.09-2.09)<br>• No significant change in AVB failure rate (requiring CS) (1.8% requiring CS before the initiative and 1.4% after the initiative)

For peer review only

5

Author	Study design / Year study conducted/ Level of significance / Adjustments	Country / Setting	Population / Number of deliveries	Intervention strategy / Frequency and duration	Effect of intervention (Kirkpatrick levels 3 or 4)
Dmello 2021	Before and after intervention study<br><br>2011-2019<br><br>P <0.05<br><br>No adjustments	Tanzania<br>22 health facilities	All women<br>(N= 74,792 at baseline, N=666,490 during intervention period-continuous)<br>Total N=741,282	Complex, dynamic, multicomponent, multifaceted intervention to improve the quality of care and survival during pregnancy and childbirth.<br>Components of AVB training: theoretical, simulation, short on-site hands-on training and prolonged on-site supervision (in some settings), provision of VE, quarterly site visits to support on-the-job coaching. At large high-volume health facilities, an on-site mentor was stationed.VE rate was systematically targeted and monitored every month, reported quarterly and annually.<br>**Frequency and duration:**<br>5-day critical BEmONC skills course; 1-day modular course on specific topics addressing gaps detected in perinatal audits; on-site trainer: an experienced nurse or doctor supported by CCBRT was periodically stationed full time for periods from 2 weeks to 1 year in the labour ward of high-volume facilities for on-site coaching and support; and on-the-job coaching (mentors would spend 2–3 days in each of the 22 sites, building skills during routine service provision).	• Significant increase in VE from 0.03% in 2011 to 3.1% in 2019 (RR 1.43; 95% CI 1.41 to 1.43)<br>• Significant increase in overall CS rate from 2.3% in 2011 to 17.6% in 2019 (RR 1.15; 95% CI 1.14 to 1.15)<br>• Significant decrease in MMR: from 154 to 79 per 100 000 live birth (RR 0.92, 95% CI 0.9 to 0.95)<br>• Significant decrease in stillbirth rate from 26 to 21 per 1000 live births (RR 0.96, 95% CI 0.96 to 0.97)<br>• Significant increase in neonatal deaths (sum of deaths among inborn and referred babies) from 5.5 in 2011 to a peak of 14.5) in 2014 and then slightly declined to 12.1 in 2019 (RR 1.05, 95% CI 1.04 to 1.05)

For peer review only

6

Author	Study design / Year study conducted/ Level of significance / Adjustments	Country / Setting	Population / Number of deliveries	Intervention strategy / Frequency and duration	Effect of intervention (Kirkpatrick levels 3 or 4)
Dominico 2018	Before and after intervention study (ITS)<br><br>2011-2016<br><br>No statistical analyses<br><br>No adjustments	Tanzania<br><br>5 Tertiary hospitals<br>10 Primary health centres	All women<br>(N=2446 at baseline, N=125,908 intervention period<br>Total N=128,354)	Multifaceted intervention comprising decentralization of life-saving services; task-shifting obstetric procedures to other cadres; training that included skill building in vacuum extraction.<br><br>Frequency and duration not stated.	• Increase in vacuum extractions from 0.15% at baseline (no statistical analyses):<br>1st year after intervention: 0.88%<br>2nd year after intervention: 2.11%<br>3rd year after intervention: 1.54%<br>4th year after intervention: 1.26%<br>5th year after intervention: 1.73%<br>• Decrease in CS rates from 11.25% at baseline (no statistical analyses):<br>1st year after intervention: 10.85%<br>2nd year after intervention: 9.42%<br>3rd year after intervention: 9.88%<br>4th year after intervention: 9.80%<br>5th year after intervention: 11.18%
Dumont 2013	Cluster-randomised trial<br><br>2007-2011<br><br>P< 0.05<br><br>OR adjusted for country, hospital type and characteristics, patient characteristics, and birthweight (for perinatal outcomes).	Mali and Senegal<br><br>Referral hospitals (23 in intervention and 23 in control)	All women<br>(N=84,924 at baseline, N=106,243 intervention period<br>Total N=191,167 deliveries)	Multifaceted intervention focused on reducing MM and improving quality of obstetric care comprising onsite training in emergency obstetric care (using Advances in Labour and Risk Management (ALARM course) and update/creation of clinical guidelines, and audit and feedback on maternal deaths,<br><br>Frequency and duration: 6 days once a year (for 2 years)	• Significant increase in overall AVB rate in Senegal from 1.24% to 2.24% in intervention groups (adjusted OR 3.10, 95% CI 1.85 to 5.20)<br>• No significant change in overall AVB rate in Mali from 3.32% to 1.97% in intervention groups, (adjusted OR 0.51, 95% CI 0.16 to 1.59)<br>• Significant decrease in intrapartum CS (adjusted OR 0.87, 95% CI 0.82 to 0.92)<br>• Significant increase in emergency antepartum CS (OR 1.33, 95% CI 1.19 to 1·50), mainly due to pre-eclampsia/eclampsia<br>• Significant decrease in hospital maternal mortality (OR 0.85, 95% CI 0.73 to 0.98)<br>• Marginally significant increase in transfusions (adjusted OR 1.44, 95% CI 0.99 to 2.11)<br>• Significant decrease in neonatal mortality < 24 h (adjusted OR 0.74, 95% CI 0.61 to 0.90)<br>• No significant change in stillbirth rates (OR 1.05, 95% CI 0.91 to 1.22)

For peer review only

7

Author	Study design / Year study conducted / Level of significance / Adjustments	Country / Setting	Population / Number of deliveries	Intervention strategy / Frequency and duration	Effect of intervention (Kirkpatrick levels 3 or 4)
Geelhoed 2018	Qualitative implementation study with presentation of before and after routine health service data.<br><br>2015-2017<br><br>No statistical analyses<br><br>No adjustments	Mozambique<br><br>Tetu Province Hospitals and primary health facilities with > 100 births per 3-months-period; this amounted to 52-82 out of a total of some 120 health facilities	All women<br>(N= 88,000 at baseline, N=265,061 intervention period<br>Total N = 353,061 births)	Multifaceted intervention (training on the management of prolonged labour including vacuum extraction; accreditation in emergency obstetric care; monitoring and evaluation of routine data from all maternities; audit and feedback of CS.<br>Clinicians at the provincial hospital were assigned to provide in-service training to any MCH nurse or doctor wishing to strengthen their capabilities.<br>Key components of AVD module:<br>1) didactic training<br>2) simulation training<br>3) Hands-on training<br>4) Audit & feedack<br><br>Duration:<br>VE training: 1 week<br>QI strategy: 3 years	• Increase in vacuum deliveries from 0.2% at baseline (no statistical analyses):<br>1st yr after intervention: 1.0%<br>2nd yr after intervention: 2.3%<br>3rd yr after intervention: 2.2%<br>• Decrease in overall CS rate from baseline rate of 2.7% (no statistical analyses):<br>1st yr after intervention: 2.1%<br>2nd yr after intervention: 2.1%<br>3rd yr after intervention: 1.8%<br><br>• Decrease in institutional maternal mortality rate of 126/100,000 births at baseline (no statistical analyses):<br>1st year after intervention: 49/100,000<br>2nd year after intervention:48/100,000<br>3rd year after intervention: 44/100,000<br><br>• Decrease in institutional stillbirth rate from baseline of 17.7/1000 newborns (no statistical analyses):<br>1st year: 14.9/1000<br>2nd year: 12.4/1000<br>3rd year: 12.2/1000
Gulmezoglu 2006	Cluster-randomised trial<br><br>2001-2002<br><br>P <0.05<br><br>No multivariable regressions. End rates adjusted by baseline rates	Mexico, Thailand<br><br>**Mexico:** 22 health facilities not associated directly with a university or other academic or research department<br><br>**Thailand:** 18 primary and secondary health facilities	**Mexico:** All women<br>(N=19,592 at baseline, N=22,236 intervention<br>Total N=41,828)<br><br>**Thailand:** All women<br>(N=17,655 at baseline, N=17,998 intervention<br>Total N=35,653)	Multifaceted educational intervention to promote the use of the WHO Reproductive Health Library<br><br>Frequency and duration:<br>Three 1-day workshops over a period of 6 months. Other components were continuous over the time of the trial	**Mexico:** No significant changes in intervention or control groups from baseline to adjusted end-of-study VE rates (intervention: 0.6% to 0.2% and control: 0.4% to 0.1%). Difference in adjusted end-of-study VE rate: 0.1, 95% CI -0.1 to 0.2<br><br>**Thailand:** No significant changes in intervention or control groups from baseline to adjusted end-of-study VE rates (intervention: 7.5% to 7.7% and control: 6.3% to 7.7%). Difference in adjusted end-of-study VE rate: 0.0, 95% CI -1.5 to 1.4

For peer review only

Author	Study design / Year study conducted / Level of significance / Adjustments	Country / Setting	Population / Number of deliveries	Intervention strategy / Frequency and duration	**Effect of intervention** (Kirkpatrick levels 3 or 4)
Mogilevkina 2022	Non-randomized controlled trial<br><br>2004-2009<br>Baseline: 2004-2005<br><br>Intervention: 2006-2007 (not analyzed)<br><br>After intervention: 2008-2009<br><br>P<0.05<br><br>No adjustments	Ukraine<br>Intervention: 28 city or district hospitals<br><br>Control: 36 city or district hospitals	All women<br><br>Intervention hospitals:<br>2004-2005: 47,838<br>2008-2009: 61,116<br><br>Control hospitals:<br>2004-2005: 38,032<br>2008-2009: 42,866	Multifaceted EMOC intervention using the Advances in Labour and Risk Management (ALARM) International Program (AIP). One 5-day course focused on the five main causes of maternal mortality. AVB training is one of the sessions.	• Significant increase in VE from 0.1% to 0.7% in intervention group (OR: 2.86; 95% CI: 1.80-4.57)<br>• No significant change in Forceps rates (OR: 1.80; 95% CI: 1.00-3.25)<br>• Significant increase in overall CS rate from 13.12% to 15.9% in intervention group (OR: 1.11; 95%CI: 1.06-1.17)<br>• MM: non-significant change<br>• PPH > 1000 ml: non-significant change<br>• Post-partum hysterectomy: non-significant change<br>• Blood transfusion: significant decrease (OR: 0.56; 95% CI: 0.48-0.65)<br>• Plasma transfusions: significant reduction (OR: 0.70; 95% CI: 0.63-0.78)<br>• Uterus explorations: significant reduction (OR: 0.64; 95% CI: 0.59-0.69)
Nolens 2016	Before and after intervention study<br><br>2012-2014<br><br>P<0.05<br><br>No adjustments	Uganda<br><br>1 Teaching hospital	Women with "medium to high risk pregnancies" (N=12,143 at baseline N= 34,894 intervention period<br>Total N=47,037)	Development of a vacuum extraction guideline, supply of vacuum extraction equipment, training of staff in didactic and simulation sessions, and on the job supervision (for 1 week, after formal training sessions) and continued continuous supervision on labour ward for 4 months during which most residents had several labour ward shift<br><br>Frequency and duration: 4 months initial training accompanied by yearly refresher (theory and simulation training session)	• Significant increase in vacuum deliveries from 0.6% to 2.1%, P<0.01<br>• Significant increase in overall CS rate from 28.2% to 30.2%, P< 0.01<br>• No significant change in CS due to obstructed labour from 6.0% to 6.0%<br>• Significant decrease in women with ruptured uterus from 1.1% to 0.8%, P< 0.01<br>• No significant change in overall maternal deaths from 0.8% to 0.7%<br>• No significant change in maternal deaths due to intrapartum complications from 0.3% to 0.2%<br>• No significant change in maternal ICU admission from 1.9% to 1.8%<br>• Significant decrease in total perinatal deaths (stillbirths + neonatal deaths during admission) from 9.2% to 8.4%, P=0.02<br>• Significant decrease in intrapartum stillbirths from 3.4% to 2.6% , P< 0.01<br>• Significant increase in term admissions to NICU from 8.7% to 10.0%, , P< 0.01<br>• No significant change in term neonatal deaths from 1.3% to 1.4%

For peer review only

9

Author	Study design / Year study conducted/ Level of significance / Adjustments	Country / Setting	Population / Number of deliveries	Intervention strategy / Frequency and duration	Effect of intervention (Kirkpatrick levels 3 or 4)
Skinner 2017	Retrospective cohort study (ITS)<br><br>2005-2014<br><br>P<0.05<br><br>No adjustments	Australia<br><br>1 Teaching hospital	All women excluding those with preterm or anomalous births, or who had CS without prior instrumental attempt (Total N=72,490 births)	Residency training program: formalized lecture series and mannequin simulation instrumental birth training and; mandatory instrumental birth credentialing for all obstetric residents	• Significant increase in forceps births from 4.8% last year pre-intervention to 9.8%-last year post-intervention (average annual rate (β) 1.5 per 100 births, 95% CI 1.03 to 1.96 per 100 births)<br>• Significant decrease in vacuum births from 8.0% last year pre-intervention to 6.0% last year post-intervention (β -1.43, 95% CI -2.5 to -0.37)<br>• Significant decrease in postpartum haemorrhage (β -1.3, 95% CI -2.07 to -0.49)<br>• No significant change in 3rd-4th degree tears (β -1.04, 95% CI -3.1 to 1.00)<br>• No significant change in composite neonatal morbiditya (β -0.18, 95% CI -0.38 to 0.02)
Solt 2011	Retrospective cohort (pre-post intervention)<br><br>Year study conducted not stated<br><br>P<0.05<br><br>No adjustments	USA<br><br>1 Teaching hospital	All women (N=3481 at baseline N=4338 intervention period Total N=7819)	Day time coverage of labour and delivery by a specially recruited senior generalist obstetrician with 35 years' clinical experience<br><br>Duration: 2 years	• Significant increase in forceps deliveries, from 4.9% to 7.8%, P < 0001<br>• Significant decrease in vacuum deliveries, from 6.4% to 2.9%, P < 0001<br>• No significant change in Overall AVB: from 11.3% to 10.6%<br>• No significant change in Overall CS, from 25.5% to 27.3%<br>• No significant change in<br>-3rd-4th degree tears, from 3.6% to 3.1%<br>-5 min Apgar < 7, from 1.9% to 2.4%<br>-Birth injuries (fracture, laceration, palsy), from 0.2% to 0.3%

For peer review only

Author	Study design / Year study conducted/ Level of significance / Adjustments	Country / Setting	Population / Number of deliveries	Intervention strategy / Frequency and duration	Effect of intervention (Kirkpatrick levels 3 or 4)
Sorensen 2010	Prospective intervention study (before and after training evaluation)<br><br>2008<br><br>P<0.05<br><br>No adjustments	Tanzania<br><br>1 Teaching hospital	All women giving birth to a neonate ≥ 1000 g (N= 558 at baseline N= 550 intervention period Total N=1108)	2-day Advanced Life Support in Obstetrics (ALSO) provider course comprising lectures, workshops, and case discussions on the major topics of emergency obstetric and neonatal care (EmONC). In the workstation on vacuum delivery, hands-on skills were practiced using pelvic and fetal mannequins and the Kiwi OmniCup	• No change in vacuum delivery rates (0% to 0%)<br>• No significant change in CS before active labor, from 2.0% to 1.8%<br>• No significant change in emergency CS, from 6.9% to 6.3%<br>• No significant change in CS for prolonged labor, from 5.1% to 4.4%<br>• Significant decrease in death of liveborn with Apgar ≥ 4, from 1.1% to 0%, P=0.03<br>• No significant change in:<br>-Neonatal deaths, from 1.2% to 0.4%<br>-Stillbirths, from 1.7% to 2.5%<br>-Neonatal resuscitation, from 3.0% to 2.4%<br>-1-min Apgar score < 7 from 6.4% to 6.9%
Takeda 2018	Before and after study, ITS<br><br>2016<br><br>No statistical analyses<br>No adjustments	Japan<br><br>1 Primary health facility	All women (N=4607 at baseline, N= 650 intervention period Total N=5257)	Forceps delivery simulation training comprising lectures with forceps delivery videos, demonstrations, and supervised simulation forceps deliveries	• Forceps rate increased from baseline 0%-2.0% in the 10 baseline pre-intervention years to 4.3% in the post-intervention year (no statistical analyses)<br>• Vacuum rates decreased from 1.2-13.3% in the 10 baseline pre-intervention years to 2.3% in post-intervention year (no statistical analyses)<br>• Overall CS rates decreased from 7.3%-25.6% in the 10 baseline pre-intervention years to 22.2% in post-intervention year (no statistical analyses)

AVB: assisted vaginal birth. CI: confidence interval. EmONC: emergency obstetric and neonatal care. ITS: interrupted times series. NICU: neonatal intensive care unit. OR: odds ratio. RR: relative risk. SD: standard deviation

a. Composite neonatal morbidity: a composite of low Apgar at 5 min (<7), neonatal intensive care unit or special care nursery admission, cephalohematoma, lacerations or abrasions, bruising, intraventricular hemorrhage, brachial plexus injury, fractured clavicle, convulsions, phototherapy, neonatal feeding problems, and respiratory distress syndrome.

For peer review only

Supplementary file 4. Assessment of the quality of 16 included studies

Risk of bias of 13 non-randomized intervention studies[a]

Study	Bias due to confounding	Bias in selection of participants into the study	Bias in classification of interventions	Bias due to deviations from intended interventions	Bias due to missing data	Bias in measurement of outcomes	Bias in selection of the result	Overall bias [b]
Bardos 2017	Low	Low	Low	Low	Low	Low	Low	Low
Becker 2020	Moderate	Low	Low	Low	Low	Low	Low	Moderate
Berglund 2010	Moderate	Low	Low	Low	Low	Low	Low	Moderate
Cottrell 2021	Serious	Serious	Low	Low	Low	Low	Low	Serious
Dmello 2021	Moderate	Low	Low	Low	Low	Low	Low	Moderate
Dominico 2018	Serious	Low	Low	Low	Low	Low	Low	Serious
Geelhoed 2018	Serious	Low	Low	Low	Low	Low	Low	Serious
Mogilevkina 2022	Moderate	Low	Low	Low	Low	Low	Low	Moderate
Nolens 2016	Serious	Low	Low	Low	Low	Low	Low	Moderate
Skinner 2017	Low	Moderate	Low	Low	Low	Low	Low	Moderate
Solt 2011	Moderate	Low	Low	Low	Low	Low	Low	Moderate
Sorensen 2010	Moderate	Low	Low	Low	Low	Low	Low	Moderate
Takeda 2018 [c]	-	-	-	-	-	-	-	-

[a] Based on Risk of Bias for Non-Randomised Studies of Interventions (ROBINS-I) tool.
[b] Interpretation of overall risk of bias.
[c]Takeda 2018:Risk of bias not assessed due to insufficient information.

Low risk of bias: The study is comparable to a well performed randomised trial.
Moderate risk of bias: The study provides sound evidence for a nonrandomized study but cannot be considered comparable to a well performed randomised trial.
Serious risk of bias: The study has some important problems.
Critical risk of bias: The study is too problematic to provide any useful evidence and should not be included in any synthesis.
No information: No information on which to base a judgement about risk of bias.

For peer review only

Risk of bias of 3 randomized trials[a]

Study	Random sequence generation (selection bias)	Allocation concealment (selection bias)	Baseline outcome measurements	Baseline characteristics	Blinding of participants and personnel (performance bias)	Blinding of outcome assessment (detection bias)	Contamination	Incomplete outcome data (attrition bias)	Selective reporting (reporting bias)	Overall bias[b]
Ameh 2014	Low	Low	Low	Low	Low	Low	Low	Low	Low	Low
Dumont 2013	Low	Low	Low	Low	Low	Low	Low	Low	Low	Low
Gulmezoglu 2006	Low	Low	Low	Low	Low	Low	Low	Low	Low	Low

[a] Based on the Cochrane EPOC Risk of bias criteria for randomised trials.
[b] Interpretation of overall risk of bias

Low risk of bias: Low risk of bias for key quality domains (i.e. allocation sequence generation and concealment)
High risk of bias: High risk of bias for one or more of the key domains
Unclear risk of bias: Unclear risk of bias for one or more key domains

For peer review only

Supplementary file 5. Baseline rates of assisted vaginal births and cesarean deliveries in studies included in the systematic review

Baseline rates[a]	N of studies	References
VE rates (N=14 studies, 15 data sets[b])		
range	0% to 8.7%	
< 1%	8	Ameh 2014 (Kenya), Dmello 2021 (Tanzania), Dominico 2018 (Tanzania), Geelhoed 2018 (Mozambique), Gulmezoglu 2006 (Mexico[c]), Mogilevkina 2022 (Ukraine), Nolens 2016 (Uganda), Sorensen 2010 (Tanzania)
1- 4 %	3	Takeda 2018 (Japan), Becker 2020 (USA), Bardos 2017 (USA)
> 4%	4	Solt 2011 (USA), Gulmezoglu 2006 (Thailand[c]), Skinner 2017 (Australia), Cottrell 2021 (USA)
Forceps rates (N=7 studies)		
range	0% to 5.2%	
< 1%	3	Bardos 2017 (USA), Mogilevkina 2022 (Ukraine), Takeda 2018 (Japan)
1-3%	2	Becker 2020 (USA), Cottrell 2021 (USA)
>3%	2	Skinner 2017 (Australia), Solt 2011 (USA)
Overall AVB rates (N=5 studies, 6 data sets[d])		
range	1.8% to 11.3%	
1-3%	3	Dumont 2013 (Senegal and Mali[e]) , Berglund 2010[f] (Ukraine)
>3 to 4.9%	1	Becker 2020 (USA)
5 to 10%	0	
>10%	2	Cottrell 2021 (USA), Solt 2011 (USA)
Overall CS rates (N=12 studies)		
range	2.6% to 30.6%	
< 10 %	3	Dmello 2021 (Tanzania), Geelhoed 2018 (Mozambique), Mogilevkina 2022[g] (Ukraine)
11-20%	1	Dominico 2018 (Tanzania)
21-30%	6	Ameh 2014 (Kenya), Solt 2011(USA), Takeda 2018 (Japan), Bardos 2017 (USA), Nolens 2016 (Uganda), Berglund 2010[f] (Ukraine)
>30%	2	Cottrell 2021 (USA), Becker 2020 (USA)

[a.] In studies with >1 time point, we present the lowest AVB rate and the highest CS rate before intervention. [b] One study (Gulmezoglu 2006) conducted in two countries (Mexico & Thailand) analyzed separately. [c] Gulmezoglu 2006 (randomized trial): rates in control groups. [d] One study (Dumont 2013) conducted in two countries (Senegal & Mali). [e] Dumont 2013 (cluster randomized trial): rates in control groups. [f] Berglund 2010: combined rates from three sites. [g] Mogilevkina 2022 (non-randomized trial): rate in control group.
AVB: Assisted vaginal birth; CS: Cesarean section; VE: Vacuum extraction

For peer review only

Supplementary file 6. Details of interventions in 16 included studies

Study	Details of intervention strategy	Health provider included in intervention strategy / Number (N)	Mode of delivery of intervention strategy	Frequency and duration of intervention strategy	AVB instrument used in strategy
Ameh 2014	Multifaceted intervention:<br>1. **EmONC training**<br>• Training using adapted version of the Life Saving Skills-Emergency Obstetric and Newborn Care (LSS-EONC) training package, a competency-based training developed specifically for low resource settings. The course covers a list of 10 topics, including AVB. A cross cutting theme throughout the training is effective communication and teamwork. In the adaptation, forceps training was excluded and only VE was taught for AVB.<br>• **Multidisciplinary training approach:** midwives, medical doctors and clinical officers are trained together by multidisciplinary teams of experienced specialist obstetricians, midwives and anaesthetists (UK based volunteers and in-country volunteers).<br>• **On-site training:** the training is delivered as close as possible to the health care providers' place of service delivery.	Obstetricians, medical doctors, clinical officers, midwives<br><br>Overall, 400 maternity care workers were trained.<br><br>In each of the 10 hospitals, 72-100% of relevant staff were trained.	Face-to-face interactive learning sessions comprising of short lectures, simulation, and hands-on sessions using low fidelity obstetric, newborn and resuscitation manikins	3 days	Vacuum

For peer review only

2

	Details of intervention strategy				
Ameh 2014, continued	2. **Local supportive supervision:** local master trainers were trained (1-day "train the trainer" course) and mentored during another EmOC training course - covering the principles of supportive supervision, tools for supportive supervision, developing and implementing action plans from the process of supportive supervision. These local master trainers (4-6 per hospital) were usually obstetricians, senior midwives, clinical based on their knowledge and potential to teach/mentor others on the job. Local supportive supervisors were expected to provide on the job support to other colleagues, facilitating behaviour change after the training.<br><br>3. **Essential equipment:** all hospitals were provided with essential equipment including 4 reusable Kiwi omnicups per hospital.				

For peer review only

Study	Details of intervention strategy	Health provider included in intervention strategy / Number (N)	Mode of delivery of intervention strategy	Frequency and duration of intervention strategy	AVB instrument used in strategy
Bardos 2017	Senior obstetricians (more than 20 years experience) teaching and supervising of residents on forceps deliveries.<br>• One to two senior obstetricians covered labor and delivery at least 5 days a week.<br>• Two senior obstetricians supervised resident deliveries during daytime (7:00 am to 7:00 pm); one supervised resident deliveries during nights and weekends (with a second obstetrician available as backup in the event of an emergency or increased volume).	Residents<br><br>N not stated	On-site, face-to-face teaching and supervision of forceps deliveries	Not stated	Forceps
Becker 2020	Assisted vaginal birth (AVB) educational curriculum consisting of didactics and simulation. The curriculum comprised twice-yearly workshops for residents (excluding first year residents). A typical workshop would have 10 to 12 participants.<br><br>Components of the workshop<br>• 28-question pretest to evaluate basic AVB knowledge; 1-hour didactic on the history, indications, safe use, and risks of forceps and vacuums; and<br>• 1-hour hands-on simulation where residents are taught how to properly execute an AVB (forceps and vacuum) using a standard maternal pelvis model and dummy fetus.<br><br>The didactic portion of the workshop is delivered to the entire group of residents. The hands-on simulation is performed in groups of three to four residents paired with one to two faculty members. After the session, residents complete a 50-question posttest, with a passing mark of 70%.	Residents<br><br>N not stated	Face-to-face lectures, hands-on simulation using a standard maternal pelvis model and dummy fetus	One 2-hour workshop offered every 6 months<br><br>Duration of hands-on training / supervision not stated	Forceps, Vacuum

For peer review only

4

Study	Details of intervention strategy	Health provider included in intervention strategy / Number (N)	Mode of delivery of intervention strategy	Frequency and duration of intervention strategy	AVB instrument used in strategy
Berglund 2010	Multifaceted intervention comprising:<br>• Continuous monitoring and evaluation system with feedback to the staff, improving medical services and counseling with special attention to resuscitation, breast feeding, and prevention of infections and to strengthen continuity of care.<br>• Development and dissemination of national, evidence-based obstetrical and neonatal protocols.<br>• Staff training on evidence-based guideline. The Russian version of the training material 'Effective Perinatal Care' (EPC), developed by WHO Regional Office for Europe was used. The material is a comprehensive, didactic, interactive two-week training package including clinical activities and a team approach to maternity care<br>• Revision of university curricula to include evidence-based perinatal care.<br>• Participating maternities were provided with basic equipment for resuscitation and neonatal care and tools for the delivery room to encourage change of position during labor and more family-oriented care.<br><br>After evaluation of the progress in the maternities by trainers, additional practical training was provided: reinforcement training in neonatal care (nine days), reinforcement in modern perinatal technology (nine days) and care in second stage in preterm delivery (one day).	Obstetricians, neonatologists, midwives, pediatric nurses, pediatricians, anesthesiologists	Face-to-face, practical clinical scenarios,	Initial Effective Perinatal Care (EPC) training: 2 weeks<br><br>Follow up EPC training: 3 days	Vacuum

For peer review only

Study	Details of intervention strategy	Health provider included in intervention strategy / Number (N)	Mode of delivery of intervention strategy	Frequency and duration of intervention strategy	AVB instrument used in strategy
Cottrell 2021	On-the-job, hands-on AVB training of residents and supervision by attending physicians.	Residents<br><br>N not stated	Hands-on training	Not stated	Forceps, Vacuum
Dmello 2021	Multiple intervention components addressed gaps across the maternal and perinatal continuum of care (training, infrastructure, routine data quality strengthening and utilization). Multiple interventions were implemented to improve the quality of Basic Emergency Obstetric Care, towards addressing maternal and perinatal mortality; specifically targetted were the EMOC signal functions that include vacuum extraction.<br><br>Components:<br>• 2-week BEmONC national training, facilitated by national trainers;<br>• 5-day critical BEmONC skills course;<br>• 1-day modular course on specific topics addressing gaps detected in perinatal audits;<br>• on-site trainer: an experienced nurse or doctor supported by CCBRT was periodically stationed full time for periods from 2 weeks to 1 year in the labour ward of high-volume facilities for on-site coaching and support;<br>• on-the-job coaching (mentors would spend 2–3 days in each of the 22 sites, building skills during routine service provision).<br>Provision of vacuum extractors was a critical component of the intervention.	Doctors and midwives.<br><br>N=1540	Face to face, videos, simulation training, group discussions, hands-on coaching	Duration of each intervention component: in 2nd column<br><br>Frequency: NI	Vacuum

For peer review only

6

Study	Details of intervention strategy	Health provider included in intervention strategy / Number (N)	Mode of delivery of intervention strategy	Frequency and duration of intervention strategy	AVB instrument used in strategy
Dominico 2018	Strategy for the 10 primary health care centers<br>• Decentralization of life-saving services from district or regional hospitals to primary health care centres. They were upgraded to provide comprehensive emergency obstetric and newborn care (C-EmONC) (i.e. they were staffed and equipped to provide obstetric surgery, blood transfusion, and basis EmONC signal functions).<br>• Task-shifting obstetric procedures from doctors, to advanced level associate clinicians or assistant medical officers (AMOs), and anaesthesia to associate clinicians or nurse anaesthetists and clinical officers.<br>Strategy for the 5 hospitals<br>• Three months clinical training (that included skill building in vacuum extraction (VE) at the two largest medical training centres in the country.<br>• Training was followed by week-long Continuing Medical Education (CME) sessions that focused on specific topics that were identified as requiring further confidence- and skills-building, such as VE; training on mannequins.<br>• Training in VE also included how to manage complications such as postpartum haemorrhage and perineal lacerations.<br>• Human resources support, clinical audit, on-going mentoring supervision.<br>• On-the-job coaching and hands-on mentoring by experts during monthly on-site visits.<br>For all institutions:<br>• Weekly conferences allowing staff to discuss specific cases and networking with others to	Assistant medical officers, nurse-midwives<br><br>N = 80	Groups of 9-14 people (face-to-face). Training was competency-based<br><br>Trainees used anatomical models for practice. They would observe procedures being performed but had only few opportunities to perform a VE on a patient<br><br>eLearning sessions were developed.<br>In the case of VE, the trainers adopted WHO's video on VE and translated it into Swahili	Not stated	Vacuum

For peer review only

	Details of intervention strategy				
Dominico 2018, continued	For all institutions:<br>• Weekly conferences allowing staff to discuss specific cases and networking with others to facilitate specialist to be "on-call" so emergency consultations were possible day and night.<br>• A WhatApp group was created.<br>• Audit and feedback about procedures and outcomes				

For peer review only

8

Study	Details of intervention strategy	Health provider included in intervention strategy / Number (N)	Mode of delivery of intervention strategy	Frequency and duration of intervention strategy	AVB instrument used in strategy
Dumont 2013	Multicomponent intervention including:<br><br>6-day training workshop provided by certified instructors in each hospital to one doctor and one midwife (train the trainer) who were responsible for maternity services.<br>• Training used ALARM (Advances in Labour and Risk Management) international course.<br>• The session consisted of 3 days of training in best practices in emergency obstetric care, 1 day of training in maternal death reviews, 1 day of awareness training related to economic, socio cultural, and ethical barriers (including sexual and reproductive rights), and 1 day of training in adult education methods.<br>• At the end of the session, a normative evaluation was done.<br>• The trainers had to attend two recertification sessions (once a year) to verify their knowledge, update them on the clinical content and process of maternal death audits, discuss their roles, share their experiences, and confirm their capacity to provide leadership in their clinical settings.<br><br>Audit and feedback on maternal deaths.<br>• A multidisciplinary audit committee including physicians, midwives, nurses, administrators was created in each participating site and trained in the process of undertaking maternal death reviews.	Doctors and midwives<br><br>N = 46	Face-to-face	6 days once a year (for 2 years)	Forceps, Vacuum

For peer review only

	Details of intervention strategy				
Dumont 2013, continued	Audit and feedback on maternal deaths (continued)<br>• The audit cycle and onsite training were then launched in each intervention site with the support of external facilitators (certified instructors) during their quarterly educational outreach visits.<br><br>If needed, local trainers who took part in the initial training workshop developed **new clinical guidelines** or updated existing guidelines according to best practices for emergency obstetric care.				

For peer review only

Study	Details of intervention strategy	Health provider included in intervention strategy / Number (N)	Mode of delivery of intervention strategy	Frequency and duration of intervention strategy	AVB instrument used in strategy
Geelhoed 2018	Four complementary interventions:<br><br>1. One-week training on the management of prolonged labour, including vacuum extraction and craniotomy, developed and facilitated by a professor considered an expert on obstetric care in low-income countries both internationally and in Mozambique. Clinicians at the provincial hospital were assigned to provide in-service training to any MCH nurse or doctor wishing to strengthen their capabilities<br><br>2. Strengthening of the quarterly emergency obstetric care accreditation process<br><br>3. Monthly audit of clinical files of all cases of caesarean section in the provincial hospital (a large majority of all caesarean sections in the province) with specific feedback to clinicians involved, copied to relevant provincial managers<br><br>4. Strict monitoring and evaluation of routine data from all maternities, with specific feedback to district health directors and doctors, copied to all and to relevant provincial manager<br><br>Sharing of capabilities between staff was encouraged. Prizes and public recognition of successful hospitals.	Provincial level: nurse-midwives, general doctors, one consultant OB-GYN specialist<br><br>District level: nurse-midwives<br><br>335,971 births (2014 to 2017)	Face-to-face (1-week training including VE)	1-week training including VE (provided only once at provincial level with a consultant OB-GYN / facilitator, and repeated in a simplified and shortened format in most of the districts in the province in the following months)<br><br>QI strategy: 3 years	Vacuum

For peer review only

Study	Details of intervention strategy	Health provider included in intervention strategy / Number (N)	Mode of delivery of intervention strategy	Frequency and duration of intervention strategy	AVB instrument used in strategy
Gulmezoglu 2006	Multifaceted intervention addressing perceived barriers to evidence-based practice.<br>• Meeting with hospital directors/heads of obstetrics and gynaecology departments before intervention for their consent and support (to ensure organisational buy in).<br>• Provision of the WHO Reproductive Health Library (RHL), computers and printer in an accessible part of the labour ward/department (to ensure easy access to the RHL).<br>• Identification of a hospital RHL coordinator from staff (to help health care workers to use RHL and to maintain links with the research team in case of problems).<br>• RHL information materials (poster, short printouts) to promote awareness in health care workers.<br>• A series of interactive workshop delivered by a specialist (to teach principles of evidence-based practice and use of RHL). Only didactic material.	Doctors (obstetricians, neonatologist, anaesthetist), midwives, interns and students<br><br>N not stated	Face-to-face workshops	Three 1-day workshops over a period of 6 months. Other components were continuous over the time of the trial	Vacuum

For peer review only

Study	Details of intervention strategy	Health provider included in intervention strategy / Number (N)	Mode of delivery of intervention strategy	Frequency and duration of intervention strategy	AVB instrument used in strategy
Mogilevkina 2022	Staff of participating hospitals were trained using the Advances in Labour and Risk Management (ALARM) International Program (AIP). The course is designed to motivate and encourage health professionals to improve the delivery of emergency obstetric care in LMIC.<br>• It consists of a 5-day course which is based on adult learning principles that promote a collaborative, multidisciplinary approach of working and learning together. It focuses on the five main causes of maternal mortality.<br>• It includes interactive plenary sessions and hands-on skill workshops with obstetrical mannequins	Total = 577 professionals<br>-342 OB-GYNs<br>-235 midwives<br><br>They worked in 28 hospitals. Coverage of staff training was 65.5%(range 28.6% to 100%).	Face-to-face<br><br>Interactive plenary sessions<br><br>Hands-on skill workshops with obstetrical mannequins	On 5-day course (AVB is one session of the whole course; no details on duration of this session)	VE and Forceps
Nolens 2016	Development of a vacuum extraction **guideline, supply of vacuum extraction** equipment, **training** of staff, and **on the job supervision**.<br><br>• Standard operating procedures (SOP) for the use of vacuum extraction and sterilization of Kiwi vacuum extractors were developed.<br>• Group training of residents in the hospital, 1 week before their rotation in labor and delivery ward.<br>• Training consisted of discussion of the SOP on vacuum extraction and sterilization, watching the WHO RHL video on vacuum extraction and skills training on mannequins.<br>• On the job supervision in the week following training.<br>• Continuous supervision on labour ward for 4 months during which most residents had several labour ward shift	Residents<br><br>N = 45	Face-to-face, small groups (4-6 residents), on site<br><br>Group presentation of the guidelines and sterilization SOP<br><br>Video (WHO) on VE and discussion<br><br>Skills training on mannequins<br><br>Continuous onsite supervision for 4 months during labour ward shifts	4 months initial training accompanied by yearly theory and a yearly skills training session	Vacuum (Kiwi OmniCup)

For peer review only

Nolens 2016, continued	• After completion of this program with a duration of 4 months, training continued according to the existing curriculum complemented with the new SOP on vacuum extraction.<br>• The existing curriculum consisted of a yearly theory session and a yearly skills training session on mannequins per year group (there were 3 year groups: year 1, 2 and 3).<br>• Both the previously trained providers (residents in year 1, 2 and 3) and the newly arrived (year 1) residents were trained according to the existing curriculum after the initial training period of four months.<br>• Yearly refresher course (theoretical and skills-mannequin training) was provided to all residents and final-year medical students.				

For peer review only

Study	Details of intervention strategy	Health provider included in intervention strategy / Number (N)	Mode of delivery of intervention strategy	Frequency and duration of intervention strategy	AVB instrument used in strategy
Skinner 2017	Residency training program: policy requiring residents to develop competency in outlet, low, mid-cavity, and rotational instrumental birth.<br>• Before 2010, resident teaching in forceps and vacuum births was predominantly case based with additional informal lectures and mannequin simulation.<br>• In 2010, formalized lecture series and mannequin simulation training were implemented preceding mandatory instrumental credentialing for all obstetric residents.<br>• Credentialing required residents to be directly supervised by senior obstetricians in human instrumental birth until assessed as competent for unsupervised practice.<br>• Residents could only be credentialed in vacuum birth after being first credentialed in forceps birth.<br>• All residents were required to meet with training supervisors at 3-monthly intervals to review credentialing documents and implement remedial pathways if credentialing was not achieved in an appropriate timeframe.	Residents<br><br>N not stated	Lecture series, mannequin simulation, performance of supervised human instrumental birth	5 years (ongoing post-training intervention)	Forceps, Vacuum

For peer review only

Study	Details of intervention strategy	Health provider included in intervention strategy / Number (N)	Mode of delivery of intervention strategy	Frequency and duration of intervention strategy	AVB instrument used in strategy
Solt 2011	Day time coverage of labour and delivery (L&D) by a specially recruited senior generalist obstetrician with 35 years' clinical experience.<br>• The obstetrician was involved in teaching all aspects of obstetrical management and appropriate use of instrumental assisted delivery.<br>• The attending physician worked as the L&D laborist 4 days (40 hours) in a week and performed night call approximately 2 to 3 times per month.	Residents<br><br>N not stated	On-site labour and delivery coverage and resident training by obstetrician	2 years	Forceps (with predominance of Simpson or Luikart-Simpson forceps)<br><br>Vacuum

For peer review only

Study	Details of intervention strategy	Health provider included in intervention strategy / Number (N)	Mode of delivery of intervention strategy	Frequency and duration of intervention strategy	AVB instrument used in strategy
Sorensen 2010	2-day Advanced Life Support in Obstetrics (ALSO) provider course.<br>• The course consisted of lectures, workshops and case discussions on the major topics of emergency obstetric and neonatal care (EmONC).<br>• Each workstation lasted 75 minutes and case discussion 30-45 minutes.<br>• Intrapartum monitoring was taught in small groups discussing 5 partograph-based case stories with focus on assessment of fetal heart rate and progress of labor.<br>• Artificial rupture of membranes and oxytocin augmentation were emphasized as actions to perform before considering caesarean delivery.<br>• In the workstation on vacuum delivery, hands-on skills were practiced using pelvic and fetal mannequins and the “Kiwi OmniCup,” a hard plastic cup with a pump integrated in the handle.<br>• Neonatal resuscitation was covered by both a lecture and a workshop.	Doctors, assistant medical officers, nurse-midwives<br><br>N = 26	Face-to-face, workshop, lectures, simulations with mannequins	2 days	Vacuum

For peer review only

Study	Details of intervention strategy	Health provider included in intervention strategy / Number (N)	Mode of delivery of intervention strategy	Frequency and duration of intervention strategy	AVB instrument used in strategy
Takeda 2018	Forceps delivery simulation training.<br>• Consisting of a lecture with forceps delivery videos, a demonstration and simulation training experience.<br>• The lecture is about pelvic examination for precise assessment of fetal head descent based on trapezoidal fetal station and is supplemented by forceps delivery videos.<br>• Then, forceps delivery is demonstrated by instructors, using a pelvic model and fetal doll.<br>• Finally, trainees attempt a simulated forceps delivery under the supervision of a specialist.	Obstetrician<br><br>N = 1	Face-to-face lecture with forceps delivery videos, a demonstration, and practicing in simulation pelvic model	Not stated	Utokyo Naegele Forceps

For peer review only

Supplementary file 7. Types of outcomes reported in the 16 included studies

Outcome	N studies (Total= 16)	References
OVD rates		
VE rates	14	Ameh 2014, Bardos 2017, Becker 2020, Cottrell 2021, Dmello 2021, Dominico 2018, Geelhoed 2018, Gulmezoglu 2016, Mogilevkina 2022, Nolens 2016, Skinner 2017, Solt 2011, Sorensen 2010, Takeda 2018
Forceps rates	7	Bardos 2017, Becker 2020, Cottrell 2021, Mogilevkina 2022, Skinner 2017, Solt 2011, Takeda 2018
Overall OVD rates	5	Berglund 2010, Becker 2020, Cottrell 2021, Dumont 2013, Solt 2011
Caesarean rates		
No information	2	Gulmezoglu 2016, Skinner 2017[a]
Overall rate	12	Ameh 2014, Bardos 2017, Becker 2020, Berglund 2010, Cottrell 2021, Dmello 2021, Dominico 2018, Geelhoed 2018, Mogilevkina 2022, Nolens 2016, Solt 2011, Takeda 2018
Antepartum/before active labour CS	2	Dumont 2018, Sorensen 2010
Emergency CS	1	Sorensen 2010
Intrapartum CS	1	Dumont 2018
CS for prolonged labour	1	Sorensen 2010
CS due to obstructed labor	1	Nolens 2016
Maternal outcomes		
None	6	Becker 2020, Berglund 2010, Dominico 2018, Gulmezoglu 2006, Sorensen 2010, Takeda 2018
Maternal mortality	6	Ameh 2014[b], Dmello 2021, Dumont 2013, Geelhoed 2018, Mogilevkina 2022, Nolens 2016
3rd/4th degree perineal tears	4	Bardos 2017, Cottrell 2021, Solt 2011, Skinner 2017
PPH or transfusion	3	Dumont 2013, Mogilevkina 2022, Skinner 2017
Uterine rupture	1	Nolens 2016
ICU admission	1	Nolens 2016
Overall maternal complication rate	1	Cottrell 2021
Perinatal outcomes		
None	5	Becker 2020, Dominico 2018, Gulmezoglu 2006, Mogilevkina 2022, Takeda 2018
Stillbirths	6	Ameh 2014, Dmello 2021, Dumont 2013, Geelhoed 2018, Nolens 2016, Sorensen 2010
Neonatal deaths	5	Berglund 2010, Dmello 2021, Dumont 2013, Nolens 2016, Sorensen 2010
Perinatal death	1	Nolens 2016
Apgar scores	3	Bardos 2017, Solt 2011, Sorensen 2010
NICU admission	3	Ameh 2014, Berglund 2010, Nolens 2016
Birth injuries	2	Cottrell 2021, Solt 2011
Neonatal resuscitation	2	Berglund 2010, Sorensen 2010
Composite neonatal complications	2	Cottrell 2021, Skinner 2017

ICU – intensive care unit; NICU – neonatal intensive care unit; PPH – post-partum hemorrhage.
[a] Skinner 2017 reported only CS rates in women who attempted AVB. [b]Ameh 2014 reported direct obstetric case fatality rate.

Supplementary file 8. Summary of effects of the interventions in 16 studies included in the review

All percentages indicate baseline x post-intervention rates or incidence. Bold terms in the cells indicate rates or outcomes with statistically significant changes after intervention (compared to baseline), according to primary study authors

Study	Changes in AVB use	Changes in rate of CS	Changes in maternal & perinatal outcomes
Ameh 2014, Kenya	**VE rate: significant increase** (from 0.2% to 1%, at 12 months), P< 0.001	Overall CS rate did not change significantly (21.5% x 21.5%, baseline Table 40).	MM (Direct obstetric case fatality rate): No significant change<br>**NICU admission for birth asphyxia: significant decrease** (48% mean reduction from 7.6% to 4% at 12 months), P = 0.03<br>**Stillbirth rate: Significant decrease (**35% mean reduction at 12 months from 5% to 3%), , P = 0.03<br>Fresh stillbirth rate: No significant change
Bardos 2017	VE rate: No significant change (3.3% to 3.3%)<br>**Forceps rate: Significant increase** (0.6% to 2.6%, aOR 8.44, 95% CI 3.1–23.1). This effect was seen only during day- time (when senior obstetrician was present).	**Overall CS rate: Significant decrease** (27.3% to 24.5%, aOR 0.68, 95% CI 0.55–0.83). This effect was seen only during day-time (when senior obstetrician was present).	3rd-4th degree tears: no significant change<br>5 min Apgar score <7: no significant change<br>Episiotomy rates increased significantly (from 3.6% to 7.4%)
Becker 2020	Overall AVB rate: no significant changes (from 3.2% to 3.1%)<br>**VE rate: significant decrease** (from 2.0% to 1.2%), P < 0.001<br>**Forceps rate: significant increase** (from 1.2% to 2.0%), P = 0.027	Overall CS rates: increase (no statistical analyses) from 27.9%-30.6% in pre-intervention years to 33.5%-33.9% in post-intervention years	NI
Berglund 2010	**Overall AVB rates**: non- significant difference in 2 sites (Lusk 2% to 2%, Lviv 0% to 0%), **significant decrease in 1 site** (Donetsk from 3.7% to 0%), P<0.0001	**Overall CS**: **significant decrease in two sites** (Donetsk 30% to 18.4%, P<0.0001; Lutsk 33% to 12.7%, P<0.0001). Non-significant change in 1 site (Lviv 21.9% to 16.9%).<br>For 3 sites combined: 29.9% to 15.5% (weighted average, no statistical analysis)	**NICU admission: significant decrease in two sites** (Lutsk 7.3% to 4.4%, P= 0.0115; Lviv 6.4% to 2.4%, P= 0.0115); no significant change in one site (Donetsk)<br>Early neonatal death: no significant change (Donetsk: from 13.6% to 9.5%; Lutsk: from 4.6% to 3.0%; Lviv: 6.4% to 0%).<br>**Neonatal resuscitation: significant decrease** (Donetsk: from 13.1% to 7.4%, P = 0.0025; Lutsk: from 2.4% to 0.9%, P<0.0001; Lviv: from 4.5% to 1.1%, P=0.0003).<br>**Episiotomies: significant decrease** in all three maternities (Donetsk: from 36.8% to 4.0%, P<0.0001; Lutsk: from 19.4% to 5.7%, P<0.0001; Lviv: from 22.2% to 7.0%, P<0.0001).<br>**Hypothermic infants: significant decrease** (Donetsk from 59.5% to 0.6%, P<0.0001; Lutsk from 85.8% to 0.3%, P<0.0001; Lviv from 77% to 0.7%, P<0.0001).

For peer review only

Study	Changes in AVB use	Changes in rate of CS	Changes in maternal & perinatal outcomes
Cottrell 2021	Overall AVB rate: no significant change (10.5 to 10.5%)<br>AVB failure rate (requiring CS): no significant change (1.8% to 1.4%)<br>**VE rate: significant decrease** (from 8.7% to 6.5%, P = 0.002.)<br>**Forceps rate: significant increase** (from 1.8% to 4.4%, P < 0.001)	Overall CS increased (30.4% to 32.1%)-no statistical analysis	3rd-degree tears: no significant change in forceps or vacuum groups<br>4th-degree tears: no significant change in vacuum group<br>**4th-degree tears: significant decrease in forceps** group (RR 0.1, 95% CI 0.01-0.73)<br>**Overall maternal complication rates: significant decrease in forceps** (RR 0.40 95% CI 0.19−0.75). No significant change in VE group.<br>Facial laceration, scalp injury, cephalo-hematoma: No significant change in forceps or vacuum groups<br>Overall neonatal complications: No significant change in forceps or vacuum groups
Dmello 2021	**VE rate: significant increase** (from 0.03% in 2011 to 3.1% in 2019; RR 1.43; 95% CI 1.41 to 1.43)	Overall rate of CS: **significant increase** (from 2.3% in 2011 to 17.6% in 2019; RR 1.15; 95% CI 1.14 to 1.15)	During the decade, facility-based:<br>**MMR: significant decrease** (from 154 to 79 per 100 000 live births, RR 0.92, 95% CI 0.9 to 0.95)<br>**Stillbirth rate: significant decrease** (from 26 to 21 per 1000 live births, RR 0.96, 95% CI 0.96 to 0.97)<br>**Neonatal deaths** (sum of deaths among inborn and referred babies)**: significant increase** (from 5.5 in 2011 to a peak of 14.5) in 2014 and then slightly declined to 12.1 in 2019; RR 1.05, 95% CI 1.04 to 1.05)
Dominico 2018	VE rate: Increase (No statistical analyses)<br>1st yr after intervention: 0.15% to 0.88%<br>2nd yr after intervention: 0.15% to 2.11%<br>3rd yr after intervention: 0.15% to 1.54%<br>4th yr after intervention: 0.15% to 1.26%<br>5th yr after intervention: 0.15% to 1.73%	Overall CS rate: Decrease (No statistical analyses)<br>1st yr after intervention: 11.25% to 10.85%<br>2nd yr after intervention: 11.25% to 9.42%<br>3rd yr after intervention: 11.25% to 9.88%<br>4th yr after intervention: 11.25% to 9.80%<br>5th yr after intervention: 11.25% to 11.18%	NI

For peer review only

Study	Changes in AVB use	Changes in rate of CS	Changes in maternal & perinatal outcomes
Dumont 2013	Overall AVB rate: no significant change (all rates < 3%)<br>Senegal: Overall AVB, **significant increase** (1.24% to 2.24%, intervention group, aOR 3.10 (95% CI 1.85-5.20)<br>Mali: Overall AVB, no significant change (3.32% to 1.97% intervention groups, aOR 0.51 (95% CI 0.16 to 1.59)	**Intrapartum CS: significant decrease** (aOR 0.87, 95% CI 0.82 - 0.92, P<0.0001)<br>**Emergency antepartum CS: significant increase** (OR 1.33; 1·19–1·50, p<0·0001) mainly due to PE/E.	**Hospital MM: significant decrease** (OR 0.85, 95% CI 0.73–0.98)<br>**Transfusions: marginally significant increase** (OR 1·44, 95% CI 0·99–2·11, p=0·06)<br>**Neonatal mortality < 24 h: significant decrease** (adjusted OR 0·74; 95% CI 0·61–0·90)<br>Stillbirth: no significant change
Geelhoed 2018	VE rate: Increase (No statistical analyses)<br>1st yr after intervention:0.2% to 1.0%<br>2nd yr after intervention: 0.2% to 2.3%<br>3rd yr after intervention: 0.2% to 2.2%	Overall CS rate: Decrease (No statistical analyses)<br>1st yr after intervention: 2.7% to 2.1%<br>2nd yr after intervention: 2.7% to 2.1%<br>3rd yr after intervention: 2.7% to 1.8%	Institutional maternal mortality rate: reduction from 126/100,000 births (baseline) to 49/100,000, 48/100,000 (1st yr), 44/100,000 (1st, 2nd and 3rd yr). No statistical analysis<br>Institutional stillbirth rate: reduction from 17.7/1000 newborns (baseline) to 14.9/1000 to 12.4/1000 to 12.2/1000 (1st, 2nd, 3rd yr). No statistical analysis.
Gulmezoglu 2006 (Mexico)	VE rate: no significant changes in intervention or control groups from baseline to adjusted end-of-study rates (intervention: 0.6% to 0.2% and ctrl: 0.4% to 0.1%)	NI	NI
Gulmezoglu 2006 (Thailand)	VE rate: no significant changes in intervention or control groups from baseline to adjusted end-of-study rates (intervention: 7.5% to 7.7%, and ctrl: 6.3% to 7.7%)	NI	NI

For peer review only

Study	Changes in AVB use	Changes in rate of CS	Changes in maternal & perinatal outcomes
Mogilevkina 2022	**VE rates: significant increase** 0.1% to 0.7% in intervention group vs 0.11% to 0.26% in the control group (Ratio of the OR: 2.86; 95% CI: 1.80-4.57<br>Forceps rates: non-significant change 0.17% to 0.08% in intervention group vs 0.21% to 0.05% in the control group (Ratio of the OR: 1.80; 95% CI: 1.00-3.25)	**Overall CS rates: significant increase** (13.12% to 15.9%) in intervention group	MM: non-significant change<br>PPH > 1000 ml: non-significant change<br>Post-partum hysterectomy: non-significant change<br>**Blood transfusion: significant decrease** (OR: 0.56; 95%CI: 0.48-0.65)<br>**Plasma transfusions: significant reduction** (OR: 0.70; 95%CI: 0.63-0.78)<br>**Uterus explorations: significant reduction** (OR: 0.64; 95%CI: 0.59-0.69)
Nolens 2016	**VE rate: significant increase** (0.6% to 2.4%, P<0.01)	**Overall CS rate: Significant Increase** (28.2% to 30.2%, P< 0.01)<br>CS rate due to obstructed labor: No change (6.0% to 6.0%)<br>Slight change in indications for CS:<br>The percentage of CS for obstructed labour decreased from 23.1% for obstructed labour to 20.0% for obstructed labour. (OR 0.92, 95% CI 0.84-1.01 (NS)	**Uterine rupture: significant decrease** (1.1% to 0.8%, P< 0.01; OR 0.75 95% CI 0.61-0.92)<br>Maternal ICU admission due to intrapartum complication: no significant change (1.9% to 1.8%)<br>Maternal death due to intrapartum complication: no significant change (0.3% to 0.2%)<br>Overall maternal deaths: no significant change (0.8% to 0.7%)<br>**Total perinatal mortality: significant decrease** (9.2% to 8.4%, P=0.02)<br>**Intrapartum stillbirths: significant decrease** (3.4% to 2.6%, P< 0.01; OR 0.76 95% CI 0.67-0.85)<br>Term neonatal deaths: no significant change (1.3% to 1.4%)<br>**Term admission to NICU: significant increase** (8.7% to 10.0%, P< 0.01, OR 1.16 95% CI 1.08-1.25)
Skinner 2017	**VE rates decreased significantly** (from 7.98%-last yr pre-intervention to 6.0%-last yr after intervention)<br>**Forceps rate increased significantly** (from 4.75%-last yr pre-intervention to 9.77%-last yr after intervention)<br>Rate of failed AVB: no significant change	NI (only reported CS in wm who attempted AVB)	**PPH: significant decrease** (-1.3, 95% CI -2.07 to -0.49)<br>3rd-4th degree tears: no significant change (-1.04, 95% CI -3.1 to 1.00)<br>Composite neonatal morbidity: no significant change (-0.18, 95% CI -0.38 to 0.02)<br>Rate of episiotomies: no significant change

For peer review only

Study	Changes in AVB use	Changes in rate of CS	Changes in maternal & perinatal outcomes
Solt 2011	Overall AVB rates. Non-significant change (11.3% to 10.6%)<br>**VE rates: significant decrease** (from 6.4% to 2.9%, P < 0001)<br>**Forceps rate: significant increase** (from 4.9% to 7.8%, P < 0001)	Overall CS: no significant change (25.5% to 27.3%)	3rd and 4th-degree tears: non-significant change (3.6% to 3.1%)<br>5 min Apgar <7 : non-significant change (1.9% to 2.4%)<br>Birth injuries: non-significant change (0.2% to 0.3%)<br>Umbilical cord pH < 7.1: significant decrease (2.4% to 1.5%)
Sorensen 2010	VE rates: No change (0% to 0%).	CS before active labor: no significant change (from 2.0% to 1.8%)<br>Emergency CS: no significant change (from 6.9% to 6.3%)<br>CS for prolonged labor: no significant change (from 5.1% to 4.4%)	**Death of liveborn with Apgar ≥ 4: significant decrease** (from 1.1% to 0%, p=0.03)<br>Neonatal deaths: No significant change (1.2% to 0.4%)<br>1-min Apgar <7: No significant change (6.4% to 6.9%)<br>Stillbirths: No significant change (1.7% to 2.5%)<br>Neonatal resuscitation: No significant change (3.0% to 2.4%)<br>Babies with mothers within 10 minutes: significant increase (from 5.6% to 71.5%)
Takeda 2018	VE rates: decrease (from 1.2%-13.3% in the 10 pre-intervention years to 2.3% after the intervention). No statistical analyses<br>Forceps rate: increase (from 0%-2,0% in the 10 pre-intervention years to 4.3% in post-intervention year). No statistical analyses	Overall CS rates: decrease after intervention (from 7.2%-25.6% in pre-intervention years to 22.2% in post-intervention year). No statistical analyses	NI

aOR: Adjusted odds ratio. AVB: Assisted vaginal birth. CI: Confidence interventional. CS: Cesarean section. Difce: Difference. ICU: Intensive care unit. MM: Maternal mortality. MMR: Maternal mortality ration. NI: No information. NICU: Neonatal intensive care unit. OR: Odds ratio. PPH: Post-partum hemorrhage. RR: Relative risk. VE: Vacuum extraction. Yr: Year

For peer review only

PRISMA 2020 Checklist

Section and Topic	Item #	Checklist item	Location where item is reported
**TITLE**			
Title	1	Identify the report as a systematic review.	p.1
**ABSTRACT**			
Abstract	2	See the PRISMA 2020 for Abstracts checklist.	p.2
**INTRODUCTION**			
Rationale	3	Describe the rationale for the review in the context of existing knowledge.	p.3
Objectives	4	Provide an explicit statement of the objective(s) or question(s) the review addresses.	p.3
**METHODS**			
Eligibility criteria	5	Specify the inclusion and exclusion criteria for the review and how studies were grouped for the syntheses.	p.4-5
Information sources	6	Specify all databases, registers, websites, organisations, reference lists and other sources searched or consulted to identify studies. Specify the date when each source was last searched or consulted.	p.5
Search strategy	7	Present the full search strategies for all databases, registers and websites, including any filters and limits used.	Supl. File 1
Selection process	8	Specify the methods used to decide whether a study met the inclusion criteria of the review, including how many reviewers screened each record and each report retrieved, whether they worked independently, and if applicable, details of automation tools used in the process.	
Data collection process	9	Specify the methods used to collect data from reports, including how many reviewers collected data from each report, whether they worked independently, any processes for obtaining or confirming data from study investigators, and if applicable, details of automation tools used in the process.	p.5
Data items	10a	List and define all outcomes for which data were sought. Specify whether all results that were compatible with each outcome domain in each study were sought (e.g. for all measures, time points, analyses), and if not, the methods used to decide which results to collect.	p.4-5
	10b	List and define all other variables for which data were sought (e.g. participant and intervention characteristics, funding sources). Describe any assumptions made about any missing or unclear information.	p.5
Study risk of bias assessment	11	Specify the methods used to assess risk of bias in the included studies, including details of the tool(s) used, how many reviewers assessed each study and whether they worked independently, and if applicable, details of automation tools used in the process.	p.5-6
Effect measures	12	Specify for each outcome the effect measure(s) (e.g. risk ratio, mean difference) used in the synthesis or presentation of results.	NA
Synthesis methods	13a	Describe the processes used to decide which studies were eligible for each synthesis (e.g. tabulating the study intervention characteristics and comparing against the planned groups for each synthesis (item #5)).	p.6
	13b	Describe any methods required to prepare the data for presentation or synthesis, such as handling of missing summary statistics, or data conversions.	p.6
	13c	Describe any methods used to tabulate or visually display results of individual studies and syntheses.	p.6
	13d	Describe any methods used to synthesize results and provide a rationale for the choice(s). If meta-analysis was performed, describe the model(s), method(s) to identify the presence and extent of statistical heterogeneity, and software package(s) used.	NA
	13e	Describe any methods used to explore possible causes of heterogeneity among study results (e.g. subgroup analysis, meta-regression).	NA
	13f	Describe any sensitivity analyses conducted to assess robustness of the synthesized results.	NA
Reporting bias assessment	14	Describe any methods used to assess risk of bias due to missing results in a synthesis (arising from reporting biases).	NA
Certainty assessment	15	Describe any methods used to assess certainty (or confidence) in the body of evidence for an outcome.	NA

For peer review only - http://bmjopen.bmj.com/site/about/guidelines.xhtml

PRISMA 2020 Checklist

Section and Topic	Item #	Checklist item	Location where item is reported
**RESULTS**			
Study selection	16a	Describe the results of the search and selection process, from the number of records identified in the search to the number of studies included in the review, ideally using a flow diagram.	p.6 and Fig 1
	16b	Cite studies that might appear to meet the inclusion criteria, but which were excluded, and explain why they were excluded.	p.6 and Supl File 2
Study characteristics	17	Cite each included study and present its characteristics.	p.6-7, p.9 Table 1-2 Supl File 3, 5-7
Risk of bias in studies	18	Present assessments of risk of bias for each included study.	p.7, Supl File 4
Results of individual studies	19	For all outcomes, present, for each study: (a) summary statistics for each group (where appropriate) and (b) an effect estimate and its precision (e.g. confidence/credible interval), ideally using structured tables or plots.	p.11-12, Table 3-4, Supl File 8
Results of syntheses	20a	For each synthesis, briefly summarise the characteristics and risk of bias among contributing studies.	NA
	20b	Present results of all statistical syntheses conducted. If meta-analysis was done, present for each the summary estimate and its precision (e.g. confidence/credible interval) and measures of statistical heterogeneity. If comparing groups, describe the direction of the effect.	NA
	20c	Present results of all investigations of possible causes of heterogeneity among study results.	NA
	20d	Present results of all sensitivity analyses conducted to assess the robustness of the synthesized results.	NA
Reporting biases	21	Present assessments of risk of bias due to missing results (arising from reporting biases) for each synthesis assessed.	NA
Certainty of evidence	22	Present assessments of certainty (or confidence) in the body of evidence for each outcome assessed.	NA
**DISCUSSION**			
Discussion	23a	Provide a general interpretation of the results in the context of other evidence.	p.14-15
	23b	Discuss any limitations of the evidence included in the review.	p.15
	23c	Discuss any limitations of the review processes used.	p.15
	23d	Discuss implications of the results for practice, policy, and future research.	p.15-16
**OTHER INFORMATION**			
Registration and protocol	24a	Provide registration information for the review, including register name and registration number, or state that the review was not registered.	p.4
	24b	Indicate where the review protocol can be accessed, or state that a protocol was not prepared.	p.4
	24c	Describe and explain any amendments to information provided at registration or in the protocol.	NA
Support	25	Describe sources of financial or non-financial support for the review, and the role of the funders or sponsors in the review.	p.16
Competing interests	26	Declare any competing interests of review authors.	p.16
Availability of data, code and other materials	27	Report which of the following are publicly available and where they can be found: template data collection forms; data extracted from included studies; data used for all analyses; analytic code; any other materials used in the review.	NA

From: Page MJ, McKenzie JE, Bossuyt PM, Boutron I, Hoffmann TC, Mulrow CD, et al. The PRISMA 2020 statement: an updated guideline for reporting systematic reviews. BMJ 2021;372:n71. doi: 10.1136/bmj.n71

For more information, visit: http://www.prisma-statement.org/

For peer review only - http://bmjopen.bmj.com/site/about/guidelines.xhtml

BMJ Open

Interventions to reintroduce or increase assisted vaginal births: a systematic review of the literature

Journal:	*BMJ Open*
Manuscript ID	bmjopen-2022-070640.R1
Article Type:	Original research
Date Submitted by the Author:	20-Jan-2023
Complete List of Authors:	Torloni, Maria Regina; Federal University of Sao Paulo, Department of Medicine<br>Opiyo, Newton; World Health Organization, UNDP/UNFPA/UNICEF/WHO/World Bank Special Programme of Research, Development and Research Training in Human Reproduction (HRP) Department of Reproductive Health and Research<br>Altieri, Elena; World Health Organization, Behavioral Insights Unit<br>Sobhy, Soha ; Queen Mary University of London, Women's Health Research Unit, Wolfson Institute of Population Health<br>Thangaratinam, Shakila; Queen Mary University of London, BARC (Barts Research Centre for Women's Health), Blizard Institute, Barts and the London School of Medicine and Dentistry; University of Birmingham, Institute of Metabolism and Systems Research<br>Nolens, Barbara; Canisius Wilhelmina Hospital,<br>Geelhoed, D. ; Tete Provincial Hospital<br>Betran, Ana Pilar; World Health Organization, UNDP/UNFPA/UNICEF/WHO/World Bank Special Programme of Research, Development and Research Training in Human Reproduction (HRP) Department of Reproductive Health and Research
**Primary Subject Heading**:	Obstetrics and gynaecology
Secondary Subject Heading:	Global health
Keywords:	OBSTETRICS, PERINATOLOGY, PUBLIC HEALTH

SCHOLARONE™
Manuscripts

BMJ

I, the Submitting Author has the right to grant and does grant on behalf of all authors of the Work (as defined in the below author licence), an exclusive licence and/or a non-exclusive licence for contributions from authors who are: i) UK Crown employees; ii) where BMJ has agreed a CC-BY licence shall apply, and/or iii) in accordance with the terms applicable for US Federal Government officers or employees acting as part of their official duties; on a worldwide, perpetual, irrevocable, royalty-free basis to BMJ Publishing Group Ltd ("BMJ") its licensees and where the relevant Journal is co-owned by BMJ to the co-owners of the Journal, to publish the Work in this journal and any other BMJ products and to exploit all rights, as set out in our licence.

The Submitting Author accepts and understands that any supply made under these terms is made by BMJ to the Submitting Author unless you are acting as an employee on behalf of your employer or a postgraduate student of an affiliated institution which is paying any applicable article publishing charge ("APC") for Open Access articles. Where the Submitting Author wishes to make the Work available on an Open Access basis (and intends to pay the relevant APC), the terms of reuse of such Open Access shall be governed by a Creative Commons licence – details of these licences and which Creative Commons licence will apply to this Work are set out in our licence referred to above.

Other than as permitted in any relevant BMJ Author's Self Archiving Policies, I confirm this Work has not been accepted for publication elsewhere, is not being considered for publication elsewhere and does not duplicate material already published. I confirm all authors consent to publication of this Work and authorise the granting of this licence.

For peer review only

Interventions to reintroduce or increase assisted vaginal births: a systematic review of the literature

Maria Regina Torloni[a], Newton Opiyo[b], Elena Altieri[c], Soha Sobhy[d], Shakila Thangaratinam[e], Barbara Nolens[f], Diederike Geelhoed[g], Ana Pilar Betrán[b]

a. Evidence Based Healthcare Post-Graduate Program, São Paulo Federal University, São Paulo, Brazil. Email: torlonimr@gmail.com

b. UNDP-UNFPA-UNICEF-WHO-World Bank Special Programme of Research, Development and Research Training in Human Reproduction (HRP), Department of Sexual and Reproductive Health and Research, World Health Organization, Geneva, Switzerland
Email: opiyon@who.int Email: betrana@who.int

c. Behavioral Insights Unit, World Health Organization, Geneva, Switzerland
Email: altierie@who.int

d. Academic clinical lecturer, Women's Health Research Unit, Wolfson Institute of Population Health, Queen Mary University of London, UK. Email: soha.sobhy@qmul.ac.uk

e. Co-Director of WHO Collaborating Centre for Global Women's Health, Institute of Metabolism and Systems Research, University of Birmingham, UK
Email: s.thangaratinam.1@bham.ac.uk

f. Canisius-Wilhelmina Hospital, Nijmegen, the Netherlands (consultant obstetrician/gynaecologist). Mulago National Referral Hospital, Kampala, Uganda (visiting)
Email: bnolens@yahoo.com

g. Formerly (2013-2017), senior technical advisor SRHR to Provincial Directorate of Health, Cidade de Tete, Tete Province, Mozambique, for Danish Ministry of Foreign Affairs (DANIDA) Email: geelhoed.d.w@gmail.com

Corresponding author: Maria Regina Torloni, Evidence Based Healthcare Post-Graduate Rua: Napoleão de Barros, 865 - Vila Clementino, CEP 04024-002 São Paulo – SP, Brazil.
Tel: 55 11 5576-4848. Email: torlonimr@gmail.com

Word count: 4250. Tables: 4, Figure: 1, References: 59

For peer review only

ABSTRACT

Objective: To synthesize the evidence from studies that implemented interventions to increase/reintroduce the use of assisted vaginal births (AVB).
Design: Systematic review (PROSPERO#CRD42020215224)
Eligibility criteria: We included experimental, semi-experimental, and observational studies that reported any intervention to reintroduce/increase AVB use.
Data sources: We searched PubMed, EMBASE, CINAHL, LILACS, Scopus, Cochrane, WHO Library, Web of Science, ClinicalTrials.gov and WHO.int/ictrp through September 2021.
Risk of bias: For trials, we used the Cochrane-EPOC tool; for other designs we used ROBINS-I.
Data extraction and synthesis: Due to heterogeneity in interventions, we did not conduct meta-analyses. We present data descriptively, grouping studies according to settings: high-income countries (HICs) or low-middle income countries (LMICs). We classified direction of intervention effects as a)statistically significant increase or decrease, b)no statistically significant change, or c)statistical significance not reported in primary study. We provide qualitative syntheses of the main barriers and enablers for success of the intervention.
Results: We included 16 studies (10 from LMICs), mostly of low or moderate methodological quality, which described interventions with various components (e.g. didactic sessions, simulation, hands-on training, guidelines, audit/feedback). All HICs studies described isolated initiatives to increase AVB use; 9/10 LMIC studies tested initiatives to increase AVB use as part of larger multicomponent interventions to improve maternal/perinatal healthcare. No study assessed women´s views or designed interventions using behavioural theories. Overall, interventions were less successful in LMICs than in HICs. Increase in AVB use was not associated with significant increase in adverse maternal or perinatal outcomes. The main barriers to the successful implementation of the initiatives were related to staff and hospital environment. **Conclusions:** There is insufficient evidence to indicate which intervention, or combination of interventions, is more effective to safely increase AVB use. More research is needed, especially in LMICs, including studies that design interventions taking into account theories of behaviour change.

Keywords: Assisted Vaginal Birth; Obstetrical Forceps; Vacuum Extraction, Obstetrical; Simulation Training; Emergency Obstetric Care; Systematic Review

Article summary: Strengths and Limitations

- This is the first systematic review on initiatives to increase the use of assisted vaginal birth, a life-saving procedure that has been steadily declining, especially in low- and middle-income countries.
- We conducted a comprehensive literature search (including grey literature), without date, publication status or language restrictions, in 10 electronic databases.
- The systematic review followed strict methodological standards including double data extraction and quality assessment of all studies.
- Many of the included studies had limited information on participant characteristics, lacked adequate statistical analyses, and did not report important outcomes.
- We created a subjective classification to categorize the core components of the interventions.

For peer review only

INTRODUCTION

Assisted vaginal birth (AVB) refers to a vaginal birth conducted with the help of an instrument, such as a forceps or a vacuum extractor (VE). Common indications for AVB include prolonged second stage, maternal exhaustion or medical indications (e.g. maternal heart disease), and suspected or confirmed foetal compromise in the second stage of labour.[1] AVB can reduce maternal and perinatal mortality and morbidity associated with complications in the second stage of labour.[2] AVB can also avoid a second-stage caesarean section (CS) and its associated short- and long-term morbidity for the mother and baby, especially in low- and middle-income countries (LMICs).[3-9] Compared to second-stage CS, AVB is associated with lower risks of haemorrhage, post-operative infection, faster maternal recovery, shorter hospital stay, and cost-savings for health systems.[10-13] Moreover, women are more likely to have a spontaneous vaginal birth in their next pregnancy after an AVB than after a CS.[14]

The benefits associated with AVB may be greater in low-resource settings where many births occur in primary care facilities with limited access to emergency obstetric care, and where the need for a CS requires a time-consuming referral to a higher level health facility, where the safety of a CS cannot be guaranteed.[13,15-17] In these settings, AVB can be a life-saving intervention for mothers and babies who are facing second stage complications. The World Health Organization (WHO) and other UN agencies have included AVB as one of the seven critical signal functions of basic emergency obstetric care (EmOC), and one of the nine signal functions of comprehensive EmOC.[18] However, reports indicate that, in most LMICs, AVB is the least likely function to be performed in primary care facilities.[19-21]

While CS rates have continued to increase in the last decades, rates of AVB have declined, particularly in LMICs.[22,23] Although unreliable data about type of birth may affect the accuracy of estimates especially in low income settings, the prevalence of AVB in high income countries (HICs) is currently 5%-20%, compared to <5% in most LMICs, with many countries having <1% of all women giving birth by AVB.[13] The reasons for the decline of AVB vary across settings and include the increasing use of epidurals for labour analgesia, lack of skilled operators and functioning equipment, concerns about trauma to the neonate and mother, fear of mother-to-child transmission of HIV, fear of litigation in case of complications, and policies that exclude some professional cadres (e.g. midwives) from performing AVB.[2,13,24,25]

In recent years, several investigators have reported efforts to increase or reintroduce AVB, particularly in LMICs, using a variety of strategies targeted at perceived barriers to the use of this procedure.[26-31] However, to our knowledge, there are no systematic reviews on this topic.

The general objective of this review was to identify, critically appraise, and synthesize the evidence from studies which implemented interventions or programs to increase or reintroduce AVB use. The specific objectives of the review were a) to assess the effectiveness and safety of strategies aimed at increasing or reintroducing AVB use, b) to identify the core elements of interventions to increase AVB use, and c) to describe the main barriers and enablers to the implementation of these initiatives, according to study authors.

For peer review only - http://bmjopen.bmj.com/site/about/guidelines.xhtml

METHODS

The protocol for this review was registered in PROSPERO (CRD42020215224).[32] We report the review according to PRISMA guidelines.[33]

Patient and Public Involvement

Patients and the public were not involved in the design, or conduct, or reporting, or dissemination plans of our systematic review.

Ethics approval

This study does not involve human participants. Ethical approval was not required for this systematic review because all data came from information freely available in the public domain.

Types of studies

We included studies that assessed the effects (benefits and harms) of interventions to reintroduce or increase AVB use with any of the following designs: randomized, semi-randomized, cluster randomized, stepped wedge, or non-randomized trials, cohort studies, cross-sectional studies, before and after intervention studies, interrupted time-series, large scale intervention case studies (implementation research studies) and mixed-methods (quantitative and qualitative) studies. Studies conducted in all settings (low-, middle-, and high-income countries) were eligible for inclusion. We excluded studies presented only as congress abstracts as these have insufficient information about the intervention and/or outcomes.

Participants

Studies involving health personnel who provide labour and delivery care to pregnant women (including qualified or trainee physicians, obstetricians, nurses, midwives, and health assistants) were eligible for inclusion. We also included studies that implemented training programs or quality improvement (QI) interventions to reintroduce or increase the use of AVB targeted at health systems, healthcare facilities, or hospitals that provide labour and delivery care. We excluded studies that involved only undergraduate students.

Interventions

Studies involving any intervention to introduce, reintroduce, or increase AVB use with any instrument were eligible for inclusion. Educational or training programs in any format and of any duration were eligible, including individual or group training, workshops, or courses conducted online or face-to-face, onsite or offsite, involving simulation, hands-on training, and on the job supervision and auditing. We also included studies that used other strategies to increase AVB use, like different models of care, task-shifting, reorganization of staff, use of opinion leaders or champions, and the provision or continued availability of functional equipment. We included studies that reported the use of specific, isolated interventions to increase AVB use as well as studies that promoted this intervention as part of larger educational/training or QI initiatives that included other components of EmOC. Eligible comparators were no intervention, usual care or practice in accordance with local protocols, or alternative interventions to reintroduce or increase the use of AVB, as reported in the primary studies.

For peer review only

Outcome measures

The primary outcome was change in the rate of AVB (overall or by specific instrument). Secondary outcomes were changes in CS rates (overall, intrapartum, and second stage CS), maternal mortality and morbidity, neonatal mortality and morbidity, maternal birth experience, health provider satisfaction with training program or QI intervention, and change in health provider AVB knowledge or skills. Studies that only reported secondary outcomes without data on the primary outcome were not included.

We only included in the review studies that stated that increase in AVB use was one of their objectives, or that change in AVB rate was one of the study outcomes (in the Methods section). All studies also had to provide data (before and after the intervention, or in the intervention and control groups) on AVBs performed by the study participants (Kirkpatrick level-3) or AVB prevalence in the hospital or setting (Kirkpatrick level-4).[34] We excluded studies that only provided data on participants´ satisfaction with the intervention (Kirkpatrick level-1) or their knowledge acquisition (Kirkpatrick level-2).[34]

Search strategy

We searched MEDLINE/PubMed, EMBASE, CINAHL, LILACS, Scopus, the Cochrane Library, WHO Library, and Web of Science using the following combination of free text and MeSH terms adapted to each database: “forceps” OR “vacuum extraction” Or “ventouse” OR “operative vaginal delivery” OR “assisted vaginal birth” AND “training” OR “programs” OR “quality improvement initiatives” (complete search strategies in Supplementary file 1). We also searched MEDLINE/PubMed using the names of specific EmOC training programs (ROBuST, ALSO, PRONTO, ESMOE). We searched two clinical trials registries (International Clinical Trials Registry Platform www.who.int/ictrp/en/ and ClinicalTrials.gov (https://clinicaltrials.gov/) for ongoing studies or completed unpublished studies. We also searched websites of key organizations involved in EmOC training (WHO, UNFPA, UNICEF, JPHIEGO, FIGO, ICM, RCOG, Pathfinder, Population Council, Advanced Life Support Group, and Childhealth Advocacy International). The searches were conducted in December 2019 and updated in September 2021. The searches were done without any language, date, or publication status restrictions. We complemented the electronic searches by screening the reference lists of all studies selected for full-text reading and related reviews, and by contacting authors of relevant abstracts for additional, potentially relevant, studies.

Process of study selection and data extraction

The identified references were entered in Covidence (https://www.covidence.org/). Four reviewers (MRT, NO, SS, APB) working in pairs independently screened titles and abstracts of all unique citations and applied the pre-specified study eligibility criteria to select studies for full-text reading. Three independent reviewers (MRT, NO, APB) read these studies and selected those that fulfilled the aforementioned inclusion criteria. The same three reviewers extracted data on key information (study design, setting, participants, interventions, outcomes, and barriers and enablers to intervention success described by the authors) independently into a pilot-tested data extraction form specifically designed for this review. In all steps of the selection and extraction process, disagreements were solved by discussion.

For peer review only - http://bmjopen.bmj.com/site/about/guidelines.xhtml

In case consensus could not be reached, a fourth reviewer was called to arbitrate.

Quality assessment of included studies

We used the criteria suggested by the Cochrane Effective Practice and Organisation of Care (EPOC) group to assess the risk of bias of the randomized trials included in the review.[35] Each trial was rated as having a "High", "Low" or "Unclear" overall risks of bias based on nine domains: random sequence generation and allocation concealment (selection bias), baseline outcome measurements and baseline characteristics, blinding of participants and personnel (performance bias), blinding of outcome assessment (detection bias), contamination, incomplete outcome data (attrition bias), and selective reporting (reporting bias). For other study designs we used the "Risk of Bias for Non-Randomised Studies of Interventions" (ROBINS-I) tool.[36] Risk of bias domains in this tool include the likelihood of bias attributable to confounding, selection of participants, classification of interventions, deviations from intended interventions, missing data, measurement of outcomes, and selection of reported results. Based on this approach, the overall risk of bias in each of these studies was rated as "Low risk", "Moderate risk", "Serious risk", "Critical risk", or "No information". Two review authors (NO, APB) independently conducted all quality assessments in duplicate. Disagreements were resolved by discussion, with the participation of a 3rd reviewer when needed. We did not exclude studies based on their risk of bias ratings.

Data synthesis

We intended to pool data from similar study designs into meta-analyses to assess the effectiveness of the intervention on the outcomes of interest. However, this was not possible because the interventions were very heterogeneous, with most individual studies using multiple different components in their educational and training interventions. Therefore, we present the data descriptively, grouping studies into two main categories according to the setting where they were conducted, i.e., LMICs or HICs, according to the World Bank.[37] We classified the direction of effect of the intervention on each outcome as a) statistically significant increase or decrease, b) no statistically significant change, or c) statistical significance not reported by the primary study authors. Statistical significance was judged using standard statistics (P values or 95% confidence intervals reported in primary studies). We list the main barriers and enablers related to the implementation or success of the intervention as reported by the study authors.

RESULTS

We identified 13,219 unique records from electronic databases and clinical trials registries. We excluded 13,177 records following review of titles and abstracts, and retrieved the full texts of the remaining 42 records for detailed eligibility assessment; 32 of these records were excluded due to various reasons (Supplementary file 2). We included in the review six additional studies not captured by the electronic search that were identified from screening the reference lists of studies selected for full text reading. Sixteen studies,15 published papers [26-31 38-46] and one PhD level thesis available online[47], presenting 18 datasets, fulfilled the selection criteria and were included in the review (Figure 1).

For peer review only

Main characteristics and quality of included studies

Six studies were conducted in three HICs (US=4 [27 38 39 44], Australia=1[43], Japan=1[45]). The other ten studies [26 28-31 40-42 46 47] were conducted in nine LMICs (Kenya, Mali, Mexico, Mozambique, Senegal, Tanzania, Thailand, Uganda and Ukraine). Most of these countries were in sub-Saharan Africa (Tanzania=3 [26 30 42], Kenya=1[47], Mozambique=1[31], Senegal and Mali=1[40], Uganda=1[29]). Two studies [40 41] were conducted in two different LMICs and their results are presented in separate datasets. The other 14 studies were conducted in a single country. Most HIC studies (5/6) were conducted in single facilities while most LMIC studies (8/10) were multisite (ranging from 3 to 82 facilities). Most of the studies involved public facilities located in urban areas, included more than one type of healthcare professionals, and used initiatives to increase the use of VE, or forceps and VE. Midwives/nurses were not included in any of the HIC studies but participated in the AVB training interventions in all of the LMIC studies except one[29] (which focused exclusively on training obstetrics and gynaecology residents). The studies were published mostly in 2010-2019 (11/16) and used non-randomized designs (13/16). Most studies were judged to have a moderate (8/16) or serious (3/16) risk of bias (Table 1). Study details and quality assessments are provided in Supplementary files 3 and 4. Fourteen studies reported baseline rates of VE that ranged from 0% to 8.7%, seven studies reported baseline rates of forceps that ranged from 0% to 5.2%, and five studies presented baselines rates of AVB in general that ranged from 1.8% to 11.3%. Overall baseline CS rates were reported by 12 studies and ranged from 2.6% to 30.6%. In general, LMICs had the lowest and HICs had the highest baseline AVB and CS rates, respectively (Supplementary file 5).

For peer review only

Table 1. Main characteristics of 16 studies on interventions to increase AVB use

Characteristic	N of studies	References
Economic category (UN)[a]		
Low income	3	Dumont 2013 (Mali), Geelhoed 2018, Nolens 2016
Lower middle income	7	Ameh 2014, Berglund 2010, Dmello 2021, Dumont 2013 (Senegal), Dominico 2018, Mogilevkina 2022, Sorensen 2010
Upper middle income	2	Gulmezoglu 2006 (Mexico and Thailand)
High income	6	Bardos 2017, Becker 2020, Cottrell 2021, Skinner 2017, Solt 2011, Takeda 2018
Type of health facility[b]		
Public	12	Ameh 2014, Becker 2020, Berglund 2010, Dmello 2021, Dominico 2018, Dumont 2013, Geelhoed 2018, Gulmezoglu 2006 (Thailand), Mogilevkina 2022, Nolens 2015, Skinner 2017, Sorensen 2010
Private	1	Solt 2011
Public and private	2	Bardos 2017, Gulmezoglu 2006 (Mexico)
Unclear or not stated	2	Cottrell 2021, Takeda 2018
Location of facility		
Urban areas	14	Ameh 2014, Bardos 2017, Becker 2020, Berglund 2010, Cottrell 2021, Dmello 2021, Dumont 2013, Gulmezoglu 2006, Mogilevkina 2022, Nolens 2016, Skinner 2017, Solt 2011, Sorensen 2010, Takeda 2018
Rural areas	1	Geelhoed 2018
Urban and rural areas	1	Dominico 2018
Number of facilities		
1	7	Bardos 2017, Becker 2020, Cottrell 2021, Nolens 2016, Solt 2011, Sorensen 2010, Takeda 2018
3	2	Berglund 2010, Skinner 2017
10-82	7	Ameh 2014, Dmello 2021, Dominico 2018, Dumont 2013, Geelhoed 2018, Gulmezoglu 2006, Mogilevkina 2022
Participants		
Resident physicians	4	Bardos 2017, Cottrell 2021, Nolens 2016, Solt 2011
Other physicians	2	Gulmezoglu 2006, Takeda 2018
>1 type of professional[c]	8	Ameh 2014, Becker 2020, Dmello 2021, Dominico 2018, Geelhoed 2018, Mogilevkina 2022, Skinner 2017, Sorensen 2010
Unclear/not stated	2	Berglund 2010, Dumont 2013
Type of instrument		
Forceps	2	Bardos 2017, Takeda 2018
Vacuum	7	Ameh 2014, Dmello 2021, Dominico 2018, Geelhoed 2018, Gulmezoglu 2006, Nolens 2016, Sorensen 2010
Forceps and vacuum	6	Becker 2020 , Cottrell 2021, Dumont 2013, Mogilevkina 2022, Skinner 2017, Solt 2011
Unclear/not stated	1	Berglund 2010
Year of publication		
2020 or later	4	Becker 2020 , Cottrell 2021, Dmello 2021, Mogilevkina 2022
2010 to 2019	11	Ameh 2014, Bardos 2017, Berglund 2010, Dominico 2018, Dumont 2013, Geelhoed 2018, Nolens 2016, Skinner 2017, Solt 2011, Sorensen 2010, Takeda 2018
2000 to 2009	1	Gulmezoglu 2006
Study design		
Randomized trials	3	Ameh 2014, Dumont 2013, Gulmezoglu 2006
Before & after studies	7	Bardos 2017, Berglund 2010, Dmello 2021, Mogilevkina 2022, Nolens 2016, Sorensen 2010, Takeda 2018
Interrupted time series	3	Dominico 2018, Geelhoed 2018, Skinner 2017
Cohort studies	3	Becker 2020, Cottrell 2021, Solt 2011
Risk of bias		
Low	4	Ameh 2014, Bardos 2017, Dumont 2013, Gulmezoglu 2006
Moderate	8	Berglund 2010, Becker 2020, Dmello 2021, Mogilevkina 2022, Nolens 2016, Skinner 2017, Solt 2011, Sorensen 2010
Serious	3	Cottrell 2021, Dominico 2018, Geelhoed 2018
Unclear[d]	1	Takeda 2018

AVB – assisted vaginal birth; EmOC – emergency obstetrics care. a. One study (Dumont 2013) conducted in two countries: Mali (low-income) and Senegal (lower middle income). b. One study (Gulmezoglu 2006) conducted in public facilities (Thailand) & public and private facilities ((Mexico). c.Doctors and nurse/midwives [Ameh 2018, Dmello 2021, Geelhoed 2018, Mogilevkina 2022]; Doctors, advance level associate clinicians and assistant medical officers [Dominico 2018]; Obstetricians and residents [Becker 2020, Skinner 2004]; Nurse-midwives, assistant medical officers, doctors [Sorensen 2010]. d Risk of bias assessment not done for one study (Takeda 2018) due to insufficient information

For peer review only

Core components of the interventions

Table 2 presents the main components of the interventions described in each study (details in Supplementary file 6). In almost all (9/10) of the studies conducted in LMICs, strategies to increase the use of AVB (mostly VE) were part of larger multicomponent interventions usually focused on improving emergency obstetric care, while all six studies from HICs tested interventions exclusively focused on increasing the use of forceps or both instruments. Didactic training (e.g. lectures, theoretical classes) about AVB was part of the interventions in all studies conducted in LMICs, and in half of those conducted in HICs. The proportion of studies that included AVB simulation training was higher in LMIC (7/10) than in HIC studies (3/6). At least half of the studies conducted in both settings included short (up to 30 days) hands-on training periods when the health care professionals conducted AVB in women under the supervision of instructors. More prolonged (>30 days) on-site hands-on supervision was part of the intervention in most (4/6) of the studies conducted in HICs, but in less than half (4/10) of the LMIC studies. The provision of AVB guidelines and equipment was part of the intervention in about one third (3/10) of the LMIC studies and in none of the HIC studies. Audit and feedback of AVB rates was a component of the intervention in almost half (4/10) of the LMIC studies and in only one of the HIC studies (Table 2).

For peer review only

Table 2. Main components of interventions to increase AVB use in 16 studies

Study	Instrument of training	Focused only on AVB	Didactic training	Simulation training	Short[a] practical hands-on training	Long[b] onsite, hands-on supervision	AVB guidelines	Provision of Equipment	Audit & feedback
10 Studies conducted in LMICs									
Ameh 2014 (Kenya)	VE	No	Yes	Yes	Yes	Yes	No	Yes	No
Berglund 2010 (Ukraine)	VE or F	No	Yes	No	No	No	Yes	No	Yes
Dmello 2021 (Tanzania)	VE	No	Yes	Yes	Yes	Yes	No	Yes	Yes
Dominico 2018 (Tanzania)	VE	No	Yes	Yes	Yes	Yes	No	No	Yes
Dumont 2013 (Senegal & Mali)	VE or F	No	Yes	Yes	No	No	Yes	No	No
Geelhoed 2018 (Mozambique)	VE	No	Yes	No	Yes	No	No	No	Yes
Gulmezoglu 2006 (Mexico & Thailand)	VE	No	Yes	No	No	No	No	No	No
Mogilevkina 2022 (Ukraine)	VE or F	No	Yes	Yes	No	No	No	No	No
Nolens 2016 (Uganda)	VE	Yes	Yes	Yes	Yes	Yes	Yes	Yes	No
Sorensen 2010 (Tanzania)	VE	No	Yes	Yes	No	No	No	No	No
6 Studies conducted in HICs									
Bardos 2017 (USA)	F	Yes	No	No	Yes	Yes	No	No	No
Becker 2020 (USA)	VE and F	Yes	Yes	Yes	No	No	No	No	No
Cottrell 2021 (USA)	VE and F	Yes	No	No	Yes	Yes	No	No	No
Skinner 2017 (Australia)	VE and F	Yes	Yes	Yes	Yes	Yes	No	No	Yes
Solt 2011 (USA)	VE and F	Yes	No	No	Yes	Yes	No	No	No
Takeda 2018 (Japan)	F	Yes	Yes	Yes	No	No	No	No	No

AVB: Assisted vaginal birth, F: forceps, VE: vacuum extraction
[a] Short: up to 30 days.
[b] Long: More than 30 days.

For peer review only

Effects of the interventions

Table 3 presents the effects of the intervention on the primary outcome (AVB use) as described by the study authors. Only two of the studies [38 40] conducted analyses taking into account possible confounding factors that could have impacted the effects of the interventions on the rates of AVB (Supplementary file 3). In LMICs, the intervention was associated with a significant increase in VE or overall AVB rates in 5 [29 40 42 46 47] of the 12 datasets from the 10 studies. Two additional studies [30 31] reported increases in VE rates but did not conduct statistical analyses. Therefore, in LMICs, interventions were associated with a successful effect (significant increase in AVB) in approximately 42% (5/12) of the datasets. Four of the 6 studies conducted in HICs reported significant increases in forceps use associated with significant decreases in VE rates. The reasons for this effect are not described in the individual studies. One study conducted in Japan[45] also reported an increase in forceps and decrease in VE but did not provide statistical analyses, and one American study[38] reported a significant increase in forceps with non-significant change in VE rates. Since all studies in HICs focused mainly on increasing forceps use, we considered that the interventions were successful (significant increase) in 83% (5/6) of the studies conducted in these settings.

Many studies did not report any of the secondary outcomes of this review (Supplementary file 7). The effects of the interventions on the rates of CS were heterogeneous, ranging from significant decrease in overall CS rates[38 40], to non-significant changes in overall CS rates[44 47] or CS for prolonged labour[26], and significant increases in overall CS[29 42 46]. However, none of the 16 studies reported the effects of the intervention on second-stage CS rates (Supplementary file 7 and 8). In most of the studies that provided this information, significant increases in the rates of AVB were not associated with significant increases in adverse maternal or perinatal outcomes (Supplementary file 8). Out of the five studies[27 38 39 43 44] conducted in HIC countries with statistically significant increases in the use of forceps, one[39] did not assess any maternal or perinatal outcomes, and one[38] reported non-significant changes in adverse maternal and perinatal outcomes (3rd/4th degree tears, low 5-minute Apgar scores). The other three HIC studies[27 43 44] reported that increased rates of forceps were associated with either non-significant changes in several adverse maternal or perinatal outcomes (3rd/4th degree tears, birth injuries, overall neonatal complications, composite neonatal morbidity, low Apgar scores) or significant decreases in some of these outcomes (4th degree tears, overall maternal complications, postpartum haemorrhage, low cord pH) in this population. In all five studies conducted in LMIC with significant increases in AVB rates[29 40 42 46 47], there were no significant increases in any of the adverse maternal outcomes reported, and several studies reported significant decreases in maternal mortality[40 42], uterine rupture[29], and transfusions[46]. One LMIC study[46] did not report any perinatal outcomes, and two studies[47 40] reported either non-significant changes or significant decreases in adverse perinatal outcomes including stillbirth, neonatal death, and admission to neonatal intensive care. The other two studies[42 29] had heterogeneous perinatal findings. One study conducted in Tanzania[42] reported that the increase in VE use was associated with a significant decrease in stillbirths along with a significant increase in neonatal deaths but this last outcome included both inborn and referred babies (who were born in other

For peer review only

settings). A study conducted Uganda[29] reported that increase in VE was associated with significant decreases in total perinatal mortality and intrapartum stillbirths, non-significant changes in neonatal deaths, and a significant increase in admissions of term infants to the neonatal intensive care unit (Supplementary file 8).

The two studies that assessed participants´ changes in knowledge and skills (Kirkpatrick level-2) reported significant increases in these outcomes immediately after the training intervention.[39 47] Only one study assessed participants´ satisfaction with the intervention (Kirkpatrick level-1) and reported that the trainees (mostly midwives) reacted positively to all lectures and breakout sessions (mean scores 9.3/10).[47] None of the studies assessed maternal birth experience or satisfaction during the interventions.

For peer review only

Table 3. Effectiveness of interventions to increase AVB use

Study	Overall AVB rate	VE rate	Forceps rate
Low- and Middle-Income countries (10 studies, 12 datasets [a])			
Ameh 2014 (Kenya)		Increase*	
Berglund 2010[b] (Ukraine)	NS change		
Dmello 2021 (Tanzania)		Increase*	
Dominico 2018 (Tanzania)		Increase	
Dumont 2013 (Mali) [a]	NS change		
Dumont 2013 (Senegal) [a]	Increase*		
Geelhoed 2018 (Mozambique)		Increase	
Gulmezoglu 2006 (Mexico) [a]		NS change	
Gulmezoglu 2006 (Thailand) [a]		NS change	
Mogilevkina 2022 (Ukraine)		Increase*	Decrease*
Nolens 2016 (Uganda)		Increase*	
Sorensen 2010 (Tanzania)		NS change	
High income countries (6 studies)			
Bardos 2017 (USA)		NS change	Increase*
Becker 2020 (USA)	NS change	Decrease*	Increase*
Cottrell 2021 (USA)	NS change	Decrease*	Increase*
Skinner 2017 (Australia)		Decrease*	Increase*
Solt 2011 (USA)	NS change	Decrease*	Increase*
Takeda 2018 (Japan)		Decrease	Increase

AVB: Assisted vaginal birth. CS: Cesarean section. NS: non- significant. VE: Vacuum extraction.
a. Multi-country study with two separate datasets. b. Effect in 2 out of 3 sites.
* Statistically significant

For peer review only

Barriers and enablers to the success of the interventions

The main barriers to the success of interventions to increase AVB use were related to healthcare professionals, environmental factors, and training. One of the main barriers reported by study authors was the difficulty in ensuring that the staff acquired and maintained all necessary skills to conduct AVB over time, including obstetrics clinical skills (correct diagnosis for the need to conduct an AVB), technical skills to safely use the instrument, and non-technical skills (communication with the woman and team). Additional barriers were the lack of continued hands-on supervision and practice, lack of institutional support for instrumental births, lack of skilled personnel (staff shortage, high turnover, need for retraining) and instruments, insufficient pain relief for women, and fear of malpractice litigation in case of adverse outcomes. The main enablers to the success of the interventions included issues related to multidisciplinary training, clarity of the roles of healthcare providers, availability of trained and skilled healthcare professionals, and involving women in decision-making (Table 4).

For peer review only

Table 4. Barriers and enablers to the implementation or success of interventions to increase AVB use

**Barrier categories**	**Barriers identified**
Health professionals	Lack of skilled personnel (due to lack of training, high staff turnover, staff shortage)<br>Lack of confidence<br>Lack of supervision in general and especially in night shifts<br>Fear of HIV transmission<br>AVB perceived as a complicated and dangerous intervention<br>Resistance to change, previous attitudes and beliefs not supportive of AVB<br>Fear of malpractice litigation if complications arise
Environment	Lack of clarity on scope of midwifery practice and functions<br>Power struggles in the maternity units<br>Unsupportive work environment
Training	Lack of theoretical and formalized education on AVB<br>Need to learn skills for correct AVB indications and when to stop trying and do a CS<br>Lack of access to repeated opportunities to practice<br>Teacher/mentor preference influence the type of instrument to use and exposure to learning opportunities<br>Lack of access to or of willing clinical mentors<br>Poor communication with mentors
Women – Healthcare provider relationship	Insufficient pain relief for women<br>Lack of acceptance by the women (associated with negative interactions with staff, poor communication, little involvement in decision-making and mistrust of caregiver)
Equipment	Lack of equipment or maintenance of equipment<br>Difficulties related to the need to sterilize instruments (unavailability of the material/equipment for this purpose)
**Enabler categories**	**Enablers identified**
Health professionals	Senior midwifery support<br>Ownership of the initiative/strategy which is facilitated by Opinion Leaders<br>Retention of trained staff
Environment	Provision of opportunities for staff to gain experience<br>In areas where there is high population resistance to CS, AVB when indicated can be popular and acceptable<br>Clarity on the scope of the midwifery practice and functions<br>Establishment of task-shifting strategies<br>Involving senior staff and hospital administrators at the planning stage<br>Local stakeholders (Ministry of Health, hospital administrators) buy-in/endorsement<br>Advocacy efforts<br>Supportive environment including close supervision, pro-active teaching by experienced colleagues enable the development of competencies
Training	Incorporating AVB training in the medical curriculum<br>Training using a range of tools and modalities including videos and simulation (these do not replace hands-on clinical teaching)<br>Training multidisciplinary teams<br>Retraining (in-service)
Women – Healthcare provider relationship	Good communication between healthcare providers and with the woman to foster trust<br>Women's and partners' involvement in decision-making and satisfactory communication between women and providers<br>Acceptance of intervention by the woman is facilitated by positive interactions with staff, respectful care and on-going communication and trust
Equipment	Availability of functioning, user friendly equipment

AVB: Assisted vaginal birth, CS: Caesarean section

For peer review only

DISCUSSION

We found relatively few studies, mostly conducted in LMICs, which reported interventions to increase the use of AVB. Due to the heterogeneity of the interventions, we could not conduct meta-analyses. In LMICs, most studies tested initiatives to increase AVB (mostly VE) as part of larger multicomponent interventions focused on improving maternal/perinatal healthcare. On the other hand, the studies conducted in HICs involved stand-alone interventions focused specifically on increasing AVB use (mostly forceps). Effectiveness differed across studies but, overall, the interventions were less successful at increasing AVB use in LMICs (where baseline rates were lower) than in HICs. Increases in operative births were not associated with significant increase in adverse maternal or perinatal outcomes, in the studies that reported these outcomes. Caution is recommended in interpreting the findings of this review because most studies had moderate or serious risk of bias. The main barriers described by primary study authors to the success of the interventions were related to training and maintaining the skills of healthcare staff, lack of instruments, and hospital environment factors.

Ensuring that participants acquired the necessary knowledge and skills to perform AVB was a core element of all interventions described in the studies. However, the diversity of methods, duration, and combinations of intervention used in the 16 studies (e.g. didactic training, simulation, hands-on supervision) precluded assessing the effectiveness of each component or combination. Although there seems to be consensus that it is essential to achieve and maintain the knowledge and skills of the professionals caring for obstetric patients, the most effective way to do this remains unknown, particularly in LMICs.[48 49] However, to a certain extent, focusing exclusively on these aspects underestimates other behavioural factors, especially social and structural environmental factors, that need to be addressed for professionals to change their behaviour. Increasing the use of AVB is influenced by decision-making processes and involves behavioural change.[50] The COM-B model, one of the most widely used frameworks to understand behaviour, proposes that three essential conditions (capability, opportunity and motivation) interact to generate any behaviour.[51] Interventions that focus exclusively on healthcare providers´ education and training (capability) may ignore other aspects, such as personal motivation and opportunity (social and physical environmental factors), that could play an important role in increasing the use of AVB. Systems thinking approaches may also help to identify behavioural and other drivers that can act as barriers.[52]

At least half of the studies involved other healthcare professionals besides doctors. As the main obstetric care providers in many LMICs, with appropriate training and patient selection, obstetric nurses and midwives can play an important role to increase or reintroduce AVB use, especially in primary healthcare settings. Moreover, in LMICs, AVB in the hands of trained and competent midwives in primary care settings may be an important pathway to empower these professionals. In many LMIC peripheral settings, midwives are expected to manage a large number of births with little support or tools in case of complications, and limited possibilities for timely referral due to problems with transport, roads, and distances.[31]

Midwife-led models of care with back-up from obstetricians, if and when necessary, could be promising approaches to safely reintroduce AVB while ensuring sustainability.[53]

None of the studies reported the views of women, including their birth experience and satisfaction, during the interventions. This is an important dimension of healthcare evaluation since patient experience has been positively associated with clinical effectiveness and safety, and is a driver of healthcare provider decision-making.[54] Dignified and respectful care is an integral part of quality of care and especially relevant when managing complications or emergencies, such as the need for AVB, when women are often most vulnerable. Evidence shows that women recognize the unpredictable nature of birth but, when interventions are needed, they want to retain a sense of personal achievement and control through active decision-making.[55] The assessment of this dimension is important to address women's needs and fears related to AVB, which can directly affect the acceptability of this intervention.

Strengths of the review include its novelty (to our knowledge, this is the first systematic review on this topic), comprehensive literature search including grey literature sources, and adherence to rigorous methodological standards including double data extraction and quality assessment. Limitations of the review pertain mostly to the characteristics of the included studies, such as the paucity of studies that assessed relevant secondary outcomes (e.g. second stage CS), or used adequate statistical analyses (e.g. a priori power calculations or multivariate regression to control for confounders), and the limited information on participant characteristics. Additionally, we cannot exclude the possibility that our search may have missed relevant studies. Finally, we created a subjective classification to categorize the core components of the interventions and we acknowledge that this classification may not be ideal, and that there are other possible ways of doing this.

Based on the findings of this review, there does not seem to be a single recipe to successfully and safely increase AVB use. There is a need for well-designed, sufficiently powered experimental or implementation research studies, especially in LMICs. Ideally, future studies should design interventions after conducting local formative research to identify relevant barriers and facilitators for the desired behavioural change in the full range of relevant cadres and health care settings. Our findings suggest that, especially in studies conducted in LMICs, interventions should include a component of hands-on, onsite, prolonged supervision, as well as continuous training/retraining of new staff. Future studies should also assess and include women´s perceptions and views on AVB in the design of the study, and evaluate participants´ satisfaction with the initiative as one of their outcomes. Additionally, future studies should assess the effects of interventions on the rates of CS in the second stage of labour, and not only on the overall CS rate. Forceps and VE were the only instruments used in the included studies, with a clear predominance of VE in LMICs. The Odón device, a novel instrument that might be easier to use by all cadres of providers, is currently being evaluated for safety and feasibility.[56] This instrument could also be tested in future studies to increase AVD use. Finally, there is a need to assess the economic impact of strategies to increase AVB use, an important point especially for complex, multifaceted interventions that require substantial changes in organizations, staffing, and model of care.

For peer review only

The identification of essential components or key features would be an important step to ensure more effective interventions. The use of Qualitative Comparative Analysis (QCA) is gaining popularity and could accelerate this process.[57] QCA is a methodology used for complex health interventions to identify if certain combinations of intervention features (e.g. type of intervention, contextual characteristics, and how the intervention was delivered) are associated with successful implementation and with improved uptake of the desired outcome, in this case, AVB.[57 58]

The review identified a paucity of studies that were grounded in behavioural theories. This contributed to the fragmented understanding of the specific influencing factors of the intended AVB behaviours, and the lack of identification of additional categories of barriers and drivers for the success of the intervention. Theories of behaviour and behaviour change provide guidance on such categories and their interaction which can help to understand the process of behaviour change before conducting any exploratory piloting and formal testing of interventions.[51 59]

CONCLUSIONS

We identified relatively few, very heterogeneous, studies with multicomponent strategies to increase AVB use. The existing evidence is insufficient to indicate which intervention, or combination of interventions, is more effective to safely increase or reintroduce the use of AVB. More research is needed, in HICs as well as in LMICs, including studies that design interventions after formative research, take into account theories of behaviour change and systems thinking, and that assess all relevant outcomes.

- **Contributorship:** All authors included on a paper fulfil the criteria of authorship. APB, MRT and NO conceived and planned the study, screened and selected the studies for inclusion, conducted data extraction and analyses, and wrote the first draft of the study. EA, SS, ST, BN, and DG made substantial contributions to data analysis and interpretation, and critically reviewed the manuscript for important intellectual content. MRT, NO, EA, SS, ST, BN, DG, and APB approved the final version of the manuscript and agree to be accountable for all aspects of the work in ensuring that questions related to the accuracy or integrity of any part of the work are appropriately investigated and resolved.
- **Competing interests:** Barbara Nolens and Diederike Geelhoed were authors of studies included in the review but were not involved in the analyses of these studies. All authors have no other conflicts of interest to declare
- **Funding:** This research was funded by the UNDP-UNFPA-UNICEF-WHO- Bank Special Programme of Research, Development and Research Training in Human Reproduction (HRP), a cosponsored programme executed by the WHO in the Department of Sexual and Reproductive Health and Research.
- **Data sharing statement:** All data relevant to the study are included in the article or available in the supplementary files.

- **Ethics approval statement:**: This study does not involve human participants. Ethical approval was not required for this systematic review because all data came from information freely available in the public domain.

References

1. Operative Vaginal Birth: ACOG Practice Bulletin, Number 219. *Obstet Gynecol* 2020;135(4):e149-e59. doi: 10.1097/AOG.0000000000003764
2. Pattinson RC, Vannevel V, Barnard D, et al. Failure to perform assisted deliveries is resulting in an increased neonatal and maternal morbidity and mortality: An expert opinion. *S Afr Med J* 2018;108(2):75-78. doi: 10.7196/SAMJ.2017.v108i2.12786
3. Rabiu KA, Adewunmi AA, Akinola OI, et al. Comparison of maternal and neonatal outcomes following caesarean section in second versus first stage of labour in a Tertiary Hospital in Nigeria. *Niger Postgrad Med J* 2011;18(3):165-71.
4. Selo-Ojeme D, Sathiyathasan S, Fayyaz M. Caesarean delivery at full cervical dilatation versus caesarean delivery in the first stage of labour: comparison of maternal and perinatal morbidity. *Arch Gynecol Obstet* 2008;278(3):245-9. doi: 10.1007/s00404-007-0548-5
5. Alexander JM, Leveno KJ, Rouse DJ, et al. Comparison of maternal and infant outcomes from primary cesarean delivery during the second compared with first stage of labor. *Obstet Gynecol* 2007;109(4):917-21. doi: 10.1097/01.AOG.0000257121.56126.fe
6. Gurung P, Malla S, Lama S, et al. Caesarean Section During Second Stage of Labor in a Tertiary Centre. *J Nepal Health Res Counc* 2017;15(2):178-81. doi: 10.3126/jnhrc.v15i2.18210
7. Bhargava S, Hooja N, Kala M, et al. Caesarean Delivery in the Second Stage of Labour at a Tertiary Care Hospital. *J Obstet Gynaecol India* 2019;69(6):558-60. doi: 10.1007/s13224-018-1179-4
8. Allen VM, O'Connell CM, Baskett TF. Maternal and perinatal morbidity of caesarean delivery at full cervical dilatation compared with caesarean delivery in the first stage of labour. *BJOG* 2005;112(7):986-90. doi: 10.1111/j.1471-0528.2005.00615.x
9. Cong A, de Vries B, Ludlow J. Does previous caesarean section at full dilatation increase the likelihood of subsequent spontaneous preterm birth? *Aust N Z J Obstet Gynaecol* 2018;58(3):267-73. doi: 10.1111/ajo.12713
10. Murphy DJ, Liebling RE, Verity L, et al. Early maternal and neonatal morbidity associated with operative delivery in second stage of labour: a cohort study. *Lancet* 2001;358(9289):1203-7. doi: 10.1016/S0140-6736(01)06341-3
11. Nolens B, Namiiro F, Lule J, et al. Prospective cohort study comparing outcomes between vacuum extraction and second-stage cesarean delivery at a Ugandan tertiary referral hospital. *Int J Gynaecol Obstet* 2018;142(1):28-36. doi: 10.1002/ijgo.12500
12. Panelli DM, Leonard SA, Joudi N, et al. Severe maternal and neonatal morbidity after attempted operative vaginal delivery. *Am J Obstet Gynecol MFM* 2021;3(3):100339. doi: 10.1016/j.ajogmf.2021.100339
13. Bailey PE, van Roosmalen J, Mola G, et al. Assisted vaginal delivery in low and middle income countries: an overview. *BJOG* 2017;124(9):1335-44. doi: 10.1111/1471-0528.14477
14. Bahl R, Strachan B, Murphy DJ. Outcome of subsequent pregnancy three years after previous operative delivery in the second stage of labour: cohort study. *BMJ* 2004;328(7435):311. doi: 10.1136/bmj.37942.546076.44
15. Sobhy S, Arroyo-Manzano D, Murugesu N, et al. Maternal and perinatal mortality and complications associated with caesarean section in low-income and middle-income countries: a systematic review and meta-analysis. *Lancet* 2019;393(10184):1973-82. doi: 10.1016/S0140-6736(18)32386-9
16. Ameh CA, Weeks AD. The role of instrumental vaginal delivery in low resource settings. *BJOG* 2009;116 Suppl 1:22-5. doi: 10.1111/j.1471-0528.2009.02331.x

For peer review only

17. Geelhoed D, Stokx J, Mariano X, et al. Risk factors for stillbirths in Tete, Mozambique. *Int J Gynaecol Obstet* 2015;130(2):148-52. doi: 10.1016/j.ijgo.2015.03.027
18. World Health Organization. Monitoring emergency obstetric care: a handbook. Geneva, Switzerland; 2009.
19. Paxton A, Bailey PE, Lobis S, Fry D. Measuring progress towards the millennium development goal for maternal health: arguing for an indicator of the health system's ability to respond to obstetric complications. Background paper commissioned by the UN millennium project task force on child health and maternal health; 2004. New York.
20. Ameh C, Msuya S, Hofman J, et al. Status of emergency obstetric care in six developing countries five years before the MDG targets for maternal and newborn health. *PLoS One* 2012;7(12):e49938. doi: 10.1371/journal.pone.0049938
21. Bailey P, Paxton A, Lobis S, et al. The availability of life-saving obstetric services in developing countries: an in-depth look at the signal functions for emergency obstetric care. *Int J Gynaecol Obstet* 2006;93(3):285-91. doi: 10.1016/j.ijgo.2006.01.028
22. Betran AP, Ye J, Moller AB, et al. The Increasing Trend in Caesarean Section Rates: Global, Regional and National Estimates: 1990-2014. *PLoS One* 2016;11(2):e0148343. doi: 10.1371/journal.pone.0148343
23. Bailey PE. The disappearing art of instrumental delivery: time to reverse the trend. *Int J Gynaecol Obstet* 2005;91(1):89-96. doi: 10.1016/j.ijgo.2005.05.016
24. Crossland N, Kingdon C, Balaam MC, et al. Women's, partners' and healthcare providers' views and experiences of assisted vaginal birth: a systematic mixed methods review. *Reprod Health* 2020;17(1):83. doi: 10.1186/s12978-020-00915-w
25. Feeley C, Crossland N, Betran AP, et al. Training and expertise in undertaking assisted vaginal delivery (AVD): a mixed methods systematic review of practitioners views and experiences. *Reprod Health* 2021;18(1):92. doi: 10.1186/s12978-021-01146-3
26. Sorensen BL, Rasch V, Massawe S, et al. Impact of ALSO training on the management of prolonged labor and neonatal care at Kagera Regional Hospital, Tanzania. *Int J Gynaecol Obstet* 2010;111(1):8-12. doi: 10.1016/j.ijgo.2010.04.031
27. Cottrell C, Clausen S, Sullivan D, et al. An Initiative to Increase Resident Performance of Operative Vaginal Deliveries. *J Obstet Gynaecol Can* 2021;43(8):1009-12. doi: 10.1016/j.jogc.2021.01.019
28. Berglund A, Lefevre-Cholay H, Bacci A, et al. Successful implementation of evidence-based routines in Ukrainian maternities. *Acta Obstet Gynecol Scand* 2010;89(2):230-7. doi: 10.3109/00016340903479894
29. Nolens B, Lule J, Namiiro F, et al. Audit of a program to increase the use of vacuum extraction in Mulago Hospital, Uganda. *BMC Pregnancy Childbirth* 2016;16:258. doi: 10.1186/s12884-016-1052-3
30. Dominico S, Bailey PE, Mwakatundu N, et al. Reintroducing vacuum extraction in primary health care facilities: a case study from Tanzania. *BMC Pregnancy Childbirth* 2018;18(1):248. doi: 10.1186/s12884-018-1888-9
31. Geelhoed D, de Deus V, Sitoe M, et al. Improving emergency obstetric care and reversing the underutilisation of vacuum extraction: a qualitative study of implementation in Tete Province, Mozambique. *BMC Pregnancy Childbirth* 2018;18(1):266. doi: 10.1186/s12884-018-1901-3
32. Torloni MR, Siaulys M, Betran AP, Sobhy S, Opiyo N, Daoud S, Thangaratinam S. Interventions to re-introduce or increase operative vaginal deliveries: a systematic review. PROSPERO 2020 CRD42020215224. https://www.crd.york.ac.uk/prospero/display_record.php?ID=CRD42020215224
33. Page MJ, McKenzie JE, Bossuyt PM, et al. The PRISMA 2020 statement: an updated guideline for reporting systematic reviews. *BMJ* 2021;372:n71. doi: 10.1136/bmj.n71
34. Kirkpatrick DL, Kirkpatrick JD. Implementing the four levels: a practical guide for effective evaluation of training programs. San Francisco, US: Berrett-Koehler Publishers, 2007.
35. Cochrane Effective Practice and Organisation of Care (EPOC). EPOC Resources for review authors, 2017. https://epoc.cochrane.org/sites/epoc.cochrane.org/files/public/uploads/Resources-for-authors2017/suggested_risk_of_bias_criteria_for_epoc_reviews.pdf Accessed on 20, May, 2021).

For peer review only - http://bmjopen.bmj.com/site/about/guidelines.xhtml

36. Sterne JA, Hernan MA, Reeves BC, et al. ROBINS-I: a tool for assessing risk of bias in non-randomised studies of interventions. *BMJ* 2016;355:i4919. doi: 10.1136/bmj.i4919
37. Fantom NJ, Serajuddin U. The World Bank's classification of countries by income (English). Policy Research working paper,no. WPS 7528 Washington, D.C. : World Bank Group. http://documents.worldbank.org/curated/en/408581467988942234/The-World-Banks-classification-of-countries-by-income.
38. Bardos J, Loudon H, Rekawek P, et al. Association Between Senior Obstetrician Supervision of Resident Deliveries and Mode of Delivery. *Obstet Gynecol* 2017;129(3):486-90. doi: 10.1097/AOG.0000000000001910
39. Becker DA, Blanchard CT, Szychowski JM, et al. Resident Operative Vaginal Delivery Volume after Educational Curriculum Implementation. *Am J Perinatol* 2020;37(13):1296-300. doi: 10.1055/s-0040-1710543
40. Dumont A, Fournier P, Abrahamowicz M, et al. Quality of care, risk management, and technology in obstetrics to reduce hospital-based maternal mortality in Senegal and Mali (QUARITE): a cluster-randomised trial. *Lancet* 2013;382(9887):146-57. doi: 10.1016/S0140-6736(13)60593-0
41. Gulmezoglu AM, Langer A, Piaggio G, et al. Cluster randomised trial of an active, multifaceted educational intervention based on the WHO Reproductive Health Library to improve obstetric practices. *BJOG* 2007;114(1):16-23. doi: 10.1111/j.1471-0528.2006.01091.x
42. Sequeira Dmello B, Sellah Z, Magembe G, et al. Learning from changes concurrent with implementing a complex and dynamic intervention to improve urban maternal and perinatal health in Dar es Salaam, Tanzania, 2011-2019. *BMJ Glob Health* 2021;6(1) doi: 10.1136/bmjgh-2020-004022
43. Skinner S, Davies-Tuck M, Wallace E, et al. Perinatal and Maternal Outcomes After Training Residents in Forceps Before Vacuum Instrumental Birth. *Obstet Gynecol* 2017;130(1):151-58. doi: 10.1097/AOG.0000000000002097
44. Solt I, Jackson S, Moore T, et al. Teaching forceps: the impact of proactive faculty. *Am J Obstet Gynecol* 2011;204(5):448 e1-4. doi: 10.1016/j.ajog.2010.12.056
45. Takeda J, Ohashi R. Effect of introducing forceps delivery at a local primary clinic. *Hypertension Research in Pregnancy* 2018;6(1):30-31. doi: 10.14390/jsshp.HRP2018-004
46. Mogilevkina I, Gurianov V, Lindmark G. Effectiveness of emergency obstetric care training at the regional level in Ukraine: a non-randomized controlled trial. *BMC Pregnancy Childbirth* 2022;22(1):145. doi: 10.1186/s12884-022-04458-9
47. Ameh CA. The effectiveness of emergency obstetric care training in Kenya. PhD thesis. University of Liverpool, 2014. https://livrepository.liverpool.ac.uk/2008539/3/AmehCha_Nov2014_2008539.pdf.
48. Ghag K, Bahl R, Winter C, et al. Key components influencing the sustainability of a multi-professional obstetric emergencies training programme in a middle-income setting: a qualitative study. *BMC Health Serv Res* 2021;21(1):384. doi: 10.1186/s12913-021-06385-5
49. Merriel A, Ficquet J, Barnard K, et al. The effects of interactive training of healthcare providers on the management of life-threatening emergencies in hospital. *Cochrane Database Syst Rev* 2019;9:CD012177. doi: 10.1002/14651858.CD012177.pub2
50. Altieri E, Grove J, Davies OL, et al. Harnessing the power of behavioural science to improve health. *Bull World Health Organ* 2021;99(11):754-54A. doi: 10.2471/BLT.21.287375
51. Michie S, van Stralen MM, West R. The behaviour change wheel: a new method for characterising and designing behaviour change interventions. *Implement Sci* 2011;6:42. doi: 10.1186/1748-5908-6-42
52. Adam T, de Savigny D. Systems thinking for strengthening health systems in LMICs: need for a paradigm shift. *Health Policy Plan* 2012;27 Suppl 4:iv1-3. doi: 10.1093/heapol/czs084
53. Long Q, Allanson ER, Pontre J, et al. Onsite midwife-led birth units (OMBUs) for care around the time of childbirth: a systematic review. *BMJ Glob Health* 2016;1(2):e000096. doi: 10.1136/bmjgh-2016-000096
54. Doyle C, Lennox L, Bell D. A systematic review of evidence on the links between patient experience and clinical safety and effectiveness. *BMJ Open* 2013;3(1) doi: 10.1136/bmjopen-2012-001570

For peer review only

55. Downe S, Finlayson K, Oladapo OT, et al. What matters to women during childbirth: A systematic qualitative review. *PLoS One* 2018;13(4):e0194906. doi: 10.1371/journal.pone.0194906
56. Hotton EJ, Alvarez M, Lenguerrand E, et al. The Odon Device for assisted vaginal birth: a feasibility study to investigate safety and efficacy-The ASSIST II study. *Pilot Feasibility Stud* 2021;7(1):72. doi: 10.1186/s40814-021-00814-2
57. Thomas J, O'Mara-Eves A, Brunton G. Using qualitative comparative analysis (QCA) in systematic reviews of complex interventions: a worked example. *Syst Rev* 2014;3:67. doi: 10.1186/2046-4053-3-67
58. Chatterley C, Javernick-Will A, Linden KG, et al. A qualitative comparative analysis of well-managed school sanitation in Bangladesh. *BMC Public Health* 2014;14:6. doi: 10.1186/1471-2458-14-6
59. Craig P, Dieppe P, Macintyre S, et al. Developing and evaluating complex interventions: the new Medical Research Council guidance. *BMJ* 2008;337:a1655. doi: 10.1136/bmj.a1655

Figure legend:

Figure 1. Flowchart of process of study identification and inclusion

List of supplementary files

Supplementary file 1: Search strategy

Supplementary file 2: Studies excluded

Supplementary file 3: Study details

Supplementary file 4: Study quality assessment

Supplementary file 5: Baseline rates

Supplementary file 6: Interventions details

Supplementary file 7: Outcomes reported in the studies

Supplementary file 8: Summary of effects

For peer review only

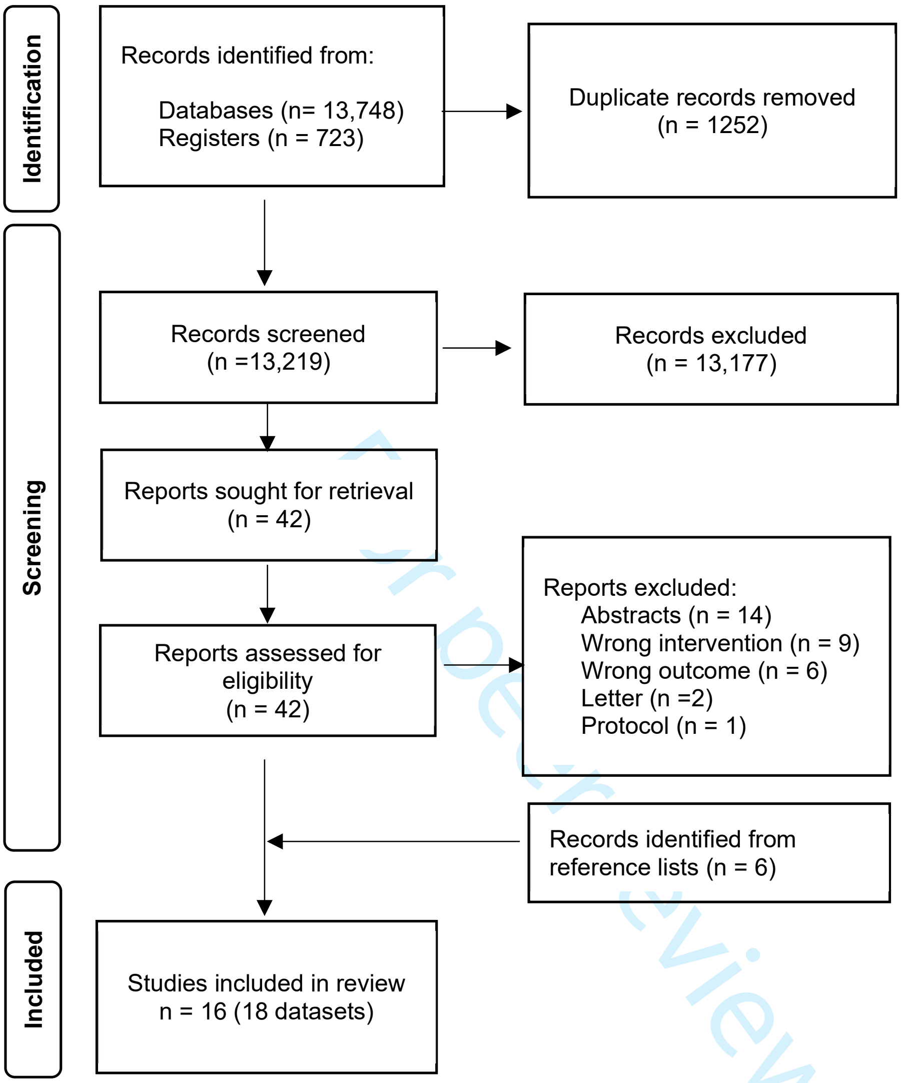
Identification
Records identified from:
Databases (n= 13,748)
Registers (n = 723)
Duplicate records removed
(n = 1252)
Screening
Records screened
(n =13,219)
Records excluded
(n = 13,177)
Reports sought for retrieval
(n = 42)
Reports assessed for eligibility
(n = 42)
Reports excluded:
Abstracts (n = 14)
Wrong intervention (n = 9)
Wrong outcome (n = 6)
Letter (n =2)
Protocol (n = 1)
Records identified from reference lists (n = 6)
Included
Studies included in review
n = 16 (18 datasets)

Supplementary file 1. Search strategy

Initial searches December 31, 2019. Updated on September 1, 2021

MEDLINE / PubMed

Searches
#1 “operative vaginal delivery” OR “operative vaginal deliver*” OR OVD [ti]
#2 “assisted vaginal delivery” OR “assisted vaginal deliver*” OR AVD [ti]
#3 “assisted vaginal birth” OR AVB [ti]
#4 “instrumental delivery” OR “instrumental deliver*” OR “instrumental vaginal delivery” OR “instrumental vaginal deliver*” [ti]
#5 “kiwi delivery” OR “kiwi deliver*” [ti]
#6 “ventouse delivery” OR “ventouse deliver*” OR “ventouse assisted delivery” OR “ventouse assisted deliver*” [ti]
#7 “vacuum delivery” OR “vacuum deliver*” OR “vacuum assisted delivery” OR “vacuum assisted deliver*” [ti]
#8 “forceps delivery” OR “forceps deliver*” OR “forceps assisted delivery” OR “forceps assisted deliver*” [ti]
#9 “obstetric forceps” OR “obstetrical forceps” [ti]
#10 ventouse OR “vacuum extraction” OR “vacuum extractor” [ti]
#11 #1 OR #2 OR #3 OR #4 OR #5 OR #6 OR #7 OR #8 OR #9 OR #10

EMBASE

Searches
#1 “operative vaginal delivery” [exp]
#2 “operative vaginal delivery” OR “operative vaginal deliver*” OR OVD [ti]
#3 “assisted vaginal delivery” [exp]
#4 “assisted vaginal delivery” OR “assisted vaginal deliver*” OR AVD [ti]
#5 “assisted vaginal birth” OR AVB [ti]
#6 “instrumental delivery” [exp] ('forceps delivery'/exp OR 'vacuum extraction'/exp)
#7 “instrumental delivery” OR “instrumental deliver*” OR “instrumental vaginal delivery” OR “instrumental vaginal deliver*” [ti]
#8 “kiwi delivery” OR “kiwi deliver*” [ti]
#9 “ventouse delivery” OR “ventouse deliver*” OR “ventouse assisted delivery” OR “ventouse assisted deliver*” [ti]
#10 “obstetric vacuum delivery kit” [exp]
#11 “obstetric vacuum delivery kit” OR “vacuum delivery” OR “vacuum deliver*” OR “vacuum assisted delivery” OR “vacuum assisted deliver*” [ti]
#12 “forceps delivery” [exp]
#13 “forceps delivery” OR “forceps deliver*” OR “forceps assisted delivery” OR “forceps assisted deliver*” [ti]
#14 “obstetric forceps” [exp]
#15 “obstetric forceps” OR “obstetrical forceps” [ti]
#16 “vacuum extraction” OR “vacuum extractor” [exp]
#17 ventouse OR “vacuum extraction” OR “vacuum extractor” [ti]
#18 #1 OR #2 OR #3 OR #4 OR #5 OR #6 OR #7 OR #8 OR #9 OR #10 OR #11 OR #12 OR #13 OR #14 OR #15 OR #16 OR #17

For peer review only - http://bmjopen.bmj.com/site/about/guidelines.xhtml

CINAHL

Searches
#1 “vaginal birth” [SU]<br>#2 “operative vaginal delivery” OR “operative vaginal deliver*” OR OVD [ti]<br>#3 “assisted vaginal delivery” OR “assisted vaginal deliver*” OR AVD [ti]<br>#4 “assisted vaginal birth” OR AVB [ti]<br>#5 “instrumental delivery” OR “instrumental deliver*” OR “instrumental vaginal delivery” OR “instrumental vaginal deliver*” [ti]<br>#6 “kiwi delivery” OR “kiwi deliver*” [ti]<br>#7 “ventouse delivery” OR “ventouse deliver*” OR “ventouse assisted delivery” OR “ventouse assisted deliver*” [ti]<br>#8 “vacuum delivery” OR “vacuum deliver*” OR “vacuum assisted delivery” OR “vacuum assisted deliver*” [ti]<br>#9 “forceps delivery” OR “forceps deliver*” OR “forceps assisted delivery” OR “forceps assisted deliver*” [ti]<br>#10 “obstetrical forceps” [SU]<br>#11 “obstetric forceps” OR “obstetrical forceps” [ti]<br>#12 “vacuum extraction, obstetrical” [SU]<br>#13 ventouse OR “vacuum extraction” OR “vacuum extractor” [ti]<br>#14 #1 OR #2 OR #3 OR #4 OR #5 OR #6 OR #7 OR #8 OR #9 OR #10 OR #11 OR #12 OR #13

LILACS

Searches
#1 “operative vaginal delivery” OR “operative vaginal deliver*” OR OVD [ti]<br>#2 “assisted vaginal delivery” OR “assisted vaginal deliver*” OR AVD [ti]<br>#3 “assisted vaginal birth” OR AVB [ti]<br>#4 “instrumental delivery” OR “instrumental deliver*” [ti]<br>#5 “instrumental vaginal delivery” OR “instrumental vaginal deliver*” [ti]<br>#6 “kiwi delivery” OR “kiwi deliver*” [ti]<br>#7 “ventouse delivery” OR “ventouse deliver*” [ti]<br>#8 “ventouse assisted delivery” OR “ventouse assisted deliver*” [ti]<br>#9 “vacuum delivery” OR “vacuum deliver*” [ti]<br>#10 “vacuum assisted delivery” OR “vacuum assisted deliver*” [ti]<br>#11 “forceps delivery” OR “forceps deliver*” [ti]<br>#12 “forceps assisted delivery” OR “forceps assisted deliver*” [ti]<br>#13 “obstetric forceps” OR “obstetrical forceps” [ti]<br>#14 ventouse OR “vacuum extraction” OR “vacuum extractor” [ti]

For peer review only

For peer review only - http://bmjopen.bmj.com/site/about/guidelines.xhtml

SCOPUS

Searches
#1 “operative vaginal delivery” OR “operative vaginal deliver*” OR OVD [ti]
#2 “assisted vaginal delivery” OR “assisted vaginal deliver*” OR AVD [ti]
#3 “assisted vaginal birth” OR AVB [ti]
#4 “instrumental delivery” OR “instrumental deliver*” OR “instrumental vaginal delivery” OR “instrumental vaginal deliver*” [ti]
#5 “kiwi delivery” OR “kiwi deliver*” [ti]
#6 “ventouse delivery” OR “ventouse deliver*” OR “ventouse assisted delivery” OR “ventouse assisted deliver*” [ti]
#7 “vacuum delivery” OR “vacuum deliver*” OR “vacuum assisted delivery” OR “vacuum assisted deliver*” [ti]
#8 “forceps delivery” OR “forceps deliver*” OR “forceps assisted delivery” OR “forceps assisted deliver*” [ti]
#9 “obstetric forceps” OR “obstetrical forceps” [ti]
#10 ventouse OR “vacuum extraction” OR “vacuum extractor” [ti]
#11 #1 OR #2 OR #3 OR #4 OR #5 OR #6 OR #7 OR #8 OR #9 OR #10

Cochrane Library

Searches
#1 “operative vaginal delivery” OR “operative vaginal deliver*” OR OVD [ti]
#2 “assisted vaginal delivery” OR “assisted vaginal deliver*” OR AVD [ti]
#3 “assisted vaginal birth” OR AVB [ti]
#4 “instrumental delivery” OR “instrumental deliver*” OR “instrumental vaginal delivery” OR “instrumental vaginal deliver*” [ti]
#5 “kiwi delivery” OR “kiwi deliver*” [ti]
#6 “ventouse delivery” OR “ventouse deliver*” OR “ventouse assisted delivery” OR “ventouse assisted deliver*” [ti]
#7 “vacuum delivery” OR “vacuum deliver*” OR “vacuum assisted delivery” OR “vacuum assisted deliver*”
#8 “forceps delivery” OR “forceps deliver*” OR “forceps assisted delivery” OR “forceps assisted deliver*” [ti]
#9 “obstetrical forceps” [MeSH]
#10 “obstetric forceps” OR “obstetrical forceps” [ti]
#11 “vacuum extraction, obstetrical” [MeSH]
#12 ventouse OR “vacuum extraction” OR “vacuum extractor” [ti]
#13 #1 OR #2 OR #3 OR #4 OR #5 OR #6 OR #7 OR #8 OR #9 OR #10 OR #11 OR #12

For peer review only - http://bmjopen.bmj.com/site/about/guidelines.xhtml

WHO Library

Searches
#1 “operative vaginal delivery” OR “operative vaginal deliver*” OR OVD [ti]<br>#2 “assisted vaginal delivery” OR “assisted vaginal deliver*” OR AVD [ti]<br>#3 “assisted vaginal birth” OR AVB [ti]<br>#4 “instrumental delivery” OR “instrumental deliver*” [ti]<br>#5 “instrumental vaginal delivery” OR “instrumental vaginal deliver*” [ti]<br>#6 “kiwi delivery” OR “kiwi deliver*” [ti]<br>#7 “ventouse delivery” OR “ventouse deliver*” [ti]<br>#8 “ventouse assisted delivery” OR “ventouse assisted deliver*” [ti]<br>#9 “vacuum delivery” OR “vacuum deliver*” [ti]<br>#10 “vacuum assisted delivery” OR “vacuum assisted deliver*” [ti]<br>#11 “forceps delivery” OR “forceps deliver*” [ti]<br>#12 “forceps assisted delivery” OR “forceps assisted deliver*” [ti]<br>#13 “obstetric forceps” OR “obstetrical forceps” [ti]<br>#14 ventouse OR “vacuum extraction” OR “vacuum extractor” [ti]<br>#15 #1 OR #2 OR #3 OR #4 OR #5 OR #6 OR #7 OR #8 OR #9 OR #10 OR #11 OR #12 OR #13 OR #14

Web of science

Searches
#1 “operative vaginal delivery” OR “operative vaginal deliver*” OR OVD [ti]<br>#2 “assisted vaginal delivery” OR “assisted vaginal deliver*” OR AVD [ti]<br>#3 “assisted vaginal birth” OR AVB [ti]<br>#4 “instrumental delivery” OR “instrumental deliver*” OR “instrumental vaginal delivery” OR “instrumental vaginal deliver*” [ti]<br>#5 “kiwi delivery” OR “kiwi deliver*” [ti]<br>#6 “ventouse delivery” OR “ventouse deliver*” OR “ventouse assisted delivery” OR “ventouse assisted deliver*” [ti]<br>#7 “vacuum delivery” OR “vacuum deliver*” OR “vacuum assisted delivery” OR “vacuum assisted deliver*” [ti]<br>#8 “forceps delivery” OR “forceps deliver*” OR “forceps assisted delivery” OR “forceps assisted deliver*” [ti]<br>#9 “obstetric forceps” OR “obstetrical forceps” [ti]<br>#10 ventouse OR “vacuum extraction” OR “vacuum extractor” [ti]<br>#11 #1 OR #2 OR #3 OR #4 OR #5 OR #6 OR #7 OR #8 OR #9 OR #10

For peer review only - http://bmjopen.bmj.com/site/about/guidelines.xhtml

MEDLINE/PubMed- Training programs

Search terms
(RCOG Operative vaginal Birth Simulation Training OR ROBuST) OR<br>(Advanced Life Support in Obstetrics OR ALSO) OR<br>(Programa de Rescate Obstétrico y Neonatal: Tratamiento Óptimo y Oportuno OR PRONTO) OR<br>(Essential Steps in the Management of Obstetric Emergencies OR ESMOE) OR<br>(assisted vaginal birth OR operative vaginal birth OR instrumental vaginal delivery OR forceps delivery OR vacuum delivery)

For peer review only

For peer review only - http://bmjopen.bmj.com/site/about/guidelines.xhtml

Supplementary file 2. Studies excluded

List of 32 studies identified through electronic search and excluded after full-text reading

Study reference	Reason for exclusion	Category for exclusion
Becker, D. A.; Blanchard, C. T.; Szychowski, J. M.; Rogers, S. L.; Brumfield, C. G.; Subramaniam, A. 743: The effect of an operative vaginal delivery curriculum for OBGYN residents American Journal of Obstetrics and Gynecology 2019;220(1):S488	Conference abstract of study later published and included (Becker 2020)	Abstract
Beneru, D.; Mcgee, T.; Jenkins, G. Caesarean sections at full dilatation before and after mandatory consultant presence. Australian and New Zealand Journal of Obstetrics and Gynaecology 2021;61(SUPPL 1):6	Conference abstract only	Abstract
Caughey, A.; Cheng, Y. W.; Zlatnik, M.; Nguy, M.; Nguyen, L.; Thiet, M. P. Perineal lacerations and operative vaginal delivery: Results from a quality improvement program American Journal of Obstetrics and Gynecology Dec 2007;197(6):S79-S79	Conference abstract only	Abstract
Coleman, L.; Basude, S. A simple program of focused education and simulation training to reduce the rate of obstetric anal sphincter injury at forceps delivery. BJOG. Apr 2015;122():78-78	Conference abstract only	Abstract
Fox, Nathan S.; Bardos, Jonah; Loudon, Holly; Rekawek, Patricia; Friedman, Frederick; Brodman, Michael. 523: Adding senior obstetrician supervision increases the forceps delivery rate and decreases the cesarean delivery rate for residents. American Journal of Obstetrics and Gynecology 2017; 216(1S):S309	Conference abstract only	Abstract
Halawani, M.; Webster, S.; Soliman, E.; El-Nouri, A. Reintroduction of operative vaginal delivery at a large tertiary referral hospital in Cairo, Egypt. International Journal of Gynecology and Obstetrics 2018;143:279	Conference abstract only	Abstract
Hodges, R.; Skinner, S.; Wallace, E.; Davies, M. Perinatal and maternal outcomes after training obstetric residents in forceps before vacuum operative birth. American Journal of Obstetrics and Gynecology 2016;214(1):S270-S271	Conference abstract of study later published and included (Skinner 2017)	Abstract
Jovic, E.; Sethna, F. Does consultant attendance for a second-stage delivery in theatre make a difference? Australian and New Zealand Journal of Obstetrics and Gynaecology 2021;61(SUPPL 1):71-72	Conference abstract only	Abstract
Muller, B.; Gilreath, N.; Wineland, R.; Sullivan, S.; Finneran, M.M. Impact of contemporary labor curves on primary cesarean delivery at a single institution American Journal of Obstetrics and Gynecology 2021;224(2):S386	Conference abstract only	Abstract
Negi, Masaru MD; Espinal, Mariana MD; Holveck, Beth RN; Mehlhaff, Krista MD; Cron, Julia MD; Pettker, Christian Michael MD Improvement of Operative Vaginal Delivery Training in Residency: A Single Institution Experience [17G], Obstetrics & Gynecology: May 2020 - Volume 135 - Issue - p 75S	Conference abstract only	Abstract
Russell, M.; Esen, U. Achieving safety and success of operative vaginal deliveries at a district teaching hospital - A triumph of audit and a targeted practical teaching workshop. International Journal of Gynecology and Obstetrics 2009;107(S2):S548	Conference abstract only	Abstract

For peer review only

Stephens-Hennessy, Beth M. Implementation of the California Maternal Quality Care Collaborative Toolkit to Support Vaginal Birth and Reduce Primary Cesarean Births JOGNN: Journal of Obstetric, Gynecologic & Neonatal Nursing 2018;47(3):S51-S52	Conference abstract only	Abstract
Wilkinson, M; Mumtaz, H; Nassar, M; Gopal, G. Does consultant presence at trials of operative vaginal delivery increase success? BJOG- 2021;128(S2):152	Conference abstract only	Abstract
Zaid, R. Z.; Yoong, W.; Lhodi, W.; Hirsch, M.; Anderson, K.; Balghari, Z.; Aziz, A.; Relph, S.; Sivashanmugarajan, V.; Govind, A. Delivering a modular, concise training package for instrumental vaginal deliveries. BJOG 2013;120(S2):594	Conference abstract only	Abstract
Dietz, Hans Peter; Ka Lai, Shek; Callaghan, Sascha; Shek, Ka Lai. Perinatal and Maternal Outcomes After Training Residents in Forceps Before Vacuum Instrumental Birth. Obstetrics & Gynecology 2017;130(4):910-910	Letter commenting study by Skinner 2017	Letter
Spencer, C.; Murphy, D.; Bewley, S. Caesarean delivery in the second stage of labour: better training in instrumental delivery may reduce rates. BMJ 2006;333(7569):613-614	Letter	Letter
van Tetering AAC, van Meurs A, Ntuyo P, van der Hout-van der Jagt MB, Mulders LGM, Nolens B, Namagambe I, Nakimuli A, Byamugisha J, Oei SG. Study protocol training for life: a stepped wedge cluster randomized trial about emergency obstetric simulation-based training in a low-income country. BMC Pregnancy Childbirth. 2020 Jul 28;20(1):429	Study protocol	Protocol
Ayala NK, Schlichting LE, Kole MB, Clark MA, Vivier PM, Viner-Brown SI, Werner EF. Operative vaginal delivery and third grade educational outcomes.Am J Obstet Gynecol MFM. 2020 Nov;2(4):100221.	Not a training or QI initiative to increase AVB	Wrong Intervention
Chang, Xavier; Chedraui, Peter; Ross, Michael G.; Hidalgo, Luis; Penafiel, Jaime Vacuum assisted delivery in Ecuador for prolonged second stage of labor: maternal-neonatal outcome The journal of maternal-fetal & neonatal medicine. 2007 2007;20(5):381-4	Not a training or QI initiative	Wrong Intervention
Chen CC, Lee JF. Effectiveness of the doula program in Northern Taiwan. Tzu Chi Med J. 2020 Apr 1;32(4):373-379.	Not a training or QI initiative to increase AVB	Wrong Intervention
Davison, Margaret Anne; Murray, Sarah; Whitaker, Lucy; Rendall, Lesley; Gammie, Nicky; Magowan, Brian. Comparison of instrumental vaginal births by assisted birth practitioner midwives and medical practitioners. British Journal of Midwifery 2014;22(10):700-705	Not a training or QI initiative to increase AVB	Wrong Intervention
Ghi T, Rizzo G; EGEO Group. The use of a hybrid mannequin for the modern high-fidelity simulation in the labor ward: the Italian experience of the Ecografia Gestione Emergenze Ostetriche (EGEO) group. Am J Obstet Gynecol. 2020 Jan;222(1):41-47.	Not a training or QI initiative to increase AVB	Wrong Intervention
Hildebrand E, Nelson M, Blomberg M. Long-term effects of the nine-item list intervention on obstetric and neonatal outcomes in Robson group 1 - A time series study. Acta Obstet Gynecol Scand. 2021 Jan;100(1):154-161.	Not a training or QI initiative to increase AVB	Wrong Intervention
Hinkson L, Henrich W, Tutschek B. Intrapartum ultrasound during rotational forceps delivery: a novel tool for safety, quality control, and teaching. Am J Obstet Gynecol. 2021 Jan;224(1):93.e1-93.e7.	Not a training or QI initiative to increase AVB	Wrong Intervention

For peer review only - http://bmjopen.bmj.com/site/about/guidelines.xhtml

Romero S, Pettersson K, Yousaf K, Westgren M, Ajne G. Perinatal outcome after vacuum assisted delivery with digital feedback on traction force; a randomised controlled study. BMC Pregnancy Childbirth. 2021 Feb 26;21(1):165. doi: 10.1186/s12884-021-03604-z. PMID: 33637058; PMCID: PMC7913459.	Not a training or QI initiative to increase AVB	Wrong Intervention
Waller-Wise R, Lewis S, Williams B. A Quality Improvement Project Utilizing a Clinical Practice Guideline in Women During Second-Stage Labor. J Perinat Educ. 2020 Apr 1;29(2):72-82. doi: 10.1891/J-PE-D-19-00014. PMID: 32308356; PMCID: PMC7159796.	Not a training or QI initiative to increase AVB	Wrong Intervention
Cheong, Y. C.; Abdullahi, H.; Lashen, H.; Fairlie, F. M. Can formal education and training improve the outcome of instrumental delivery? European Journal of Obstetrics and Gynecology and Reproductive Biology 2004;113(2):139-144	Increase of AVB use was not one of the Objectives or cited as one of the Outcomes in Methods	Wrong Outcome
Chikazawa, K.; Takagi, K.; Takahashi, H.; Akashi, K.; Nakamura, E.; Samejima, K.; Ushijima, J.; Horiuchi, I. Introduction of forceps delivery education for residents at a single perinatal institution. Hypertension Research in Pregnancy 2016;4(2):102-105	No data on AVB use before x after intervention	Wrong Outcome
Coste Mazeau, P; Boukeffa, N; Ticaud Boileau, N; Huet, S; Traverse, M; Eyraud, JL; Laguerre, A; Catalan, C; Riedl, C Evaluation of Suzor forceps training by studying obstetric anal sphincter injuries: a retrospective study. BMC Pregnancy Childbirth 20, 674 (2020)	No data on AVB use before x after intervention	Wrong Outcome
Dolo O, Clack A, Gibson H, Lewis N, Southall DP. Training of midwives in advanced obstetrics in Liberia. Bull World Health Organ. 2016 May 1;94(5):383-7.	No data on AVB use before x after intervention	Wrong Outcome
Egami N, Muta R, Anami A, Koga H. Impact of clinical practice guidelines for vacuum-assisted delivery on maternal and neonatal outcomes in Japan: A single-center observational study. J Obstet Gynaecol Res. 2021 Jan;47(1):167-173	No data on AVB use before x after intervention (assessed only success rates).	Wrong Outcome
van Tetering AAC, Segers MHM, Ntuyo P, Namagambe I, van der Hout-van der Jagt MB, Byamugisha JK, Oei SG. Evaluating the Instructional Design and Effect on Knowledge, Teamwork, and Skills of Technology-Enhanced Simulation-Based Training in Obstetrics in Uganda: Stepped-Wedge Cluster Randomized Trial. JMIR Med Educ. 2021 Feb 5;7(1):e17277	Present only Kirpatrick 1 and 2 outcomes	Wrong outcome

For peer review only

Supplementary file 3. Details of 16 studies on interventions to increase AVB use

Author	Study design / Year study conducted / Level of significance / Adjustments	Country / Setting	Population / Number of deliveries	Intervention strategy / Frequency and duration	Effect of intervention (Kirkpatrick levels 3 or 4)
Ameh 2014	Cluster-randomised trial (stepped wedge)<br><br>2009-2011<br><br>P < 0.05<br><br>Adjustment for clustering	Kenya<br><br>2 "level 5" hospitals and 8 regional hospitals<br><br>All health care facilities involved were provincial "referral hospitals"	All women (N=16,794 at baseline, N=17,404 intervention period Total N= 34,198)	Multifaceted intervention comprising training in emergency obstetric and neonatal care (EmONC), provision of EmOC equipment (including reusable Kiwi omnicups) and local supportive supervision.<br><br>Duration of training: 3 days	• Significant increase in vacuum extraction (from 0.2% to 1% at 12 months) P< 0.001<br>• No significant change in overall CS (baseline 21.5% vs. post-intervention 21.5%)<br>• No significant change in direct obstetric case fatality rate (from 3% to 2%)<br>• Significant decrease in NICU admission due to birth asphyxia (48% mean reduction at 12 months, from 7.6% to 4%) P = 0.03<br>• Significant decrease in stillbirth rate at 12 months (35% mean reduction at 12 months, from 5% to 3%) P = 0.03<br>• No significant change in fresh stillbirth rate (from 2.1% to 1.9%)
Bardos 2017	Before and after study<br><br>2011-2015<br><br>P< 0.05<br>Confounding variables included in multivariable logistic regression: maternal age $\geq$ 35, maternal obesity (BMI$\geq$ 35 Kg/m2 at delivery), induction of labor, parity, and prior cesarean delivery. The last two variables were not included in the analysis of nulliparous patients only.	USA<br><br>1 Teaching hospital	Term, singleton, cephalic births cared by residents (N=1919 at baseline, N=3282 intervention period, Total N=5201)	Senior obstetrician (>20 years experience) teaching and supervising residents on forceps deliveries during the day time.<br><br>Frequency and duration not stated.	• Significant increase in forceps deliveries (0.6% to 2.6%, aOR 8.44, 95% CI 3.1 to 23.1)<br>• No significant change in vacuum deliveries (3.3% to 3.3%)<br>• Significant decrease in overall CS (27.3% to 24.5%, aOR 0.68, 95% CI 0.55 to 0.83)<br>• No significant change in:<br>-3rd-4th degree tears (1.4% to 2.0%)<br>-5 min Apgar score < 7 (0.3% to 0.5%)<br>The increase in forceps deliveries and the decrease in CS were seen only in daytime hours (7 AM to 7 PM), that is, the shift that was covered by senior obstetricians

For peer review only

2

Author	Study design / Year study conducted/ Level of significance / Adjustments	Country / Setting	Population / Number of deliveries	Intervention strategy / Frequency and duration	Effect of intervention (Kirkpatrick levels 3 or 4)
Becker 2020	Retrospective cohort study<br><br>2011-2018<br><br>P< 0.05<br><br>No adjustments	USA<br><br>1 Teaching hospital	All women with births after 20 weeks gestation (N=20,471 at baseline, N=6106 intervention period, Total N=26,577)	Twice-yearly AVB residency curriculum consisting of didactics (pretest and posttest of basic AVB knowledge, history, indications, safe use, risks of forceps and vacuum) and hands-on simulation of AVB.<br><br>Frequency and duration: One 2-hour workshop offered every 6 months	• Significant increase in forceps use, mean (SD), pre-1.2% (0.6) vs. post 2.0% (0.9), P = 0.027<br>• Significant decrease in vacuum use, mean (SD), pre-2.0% (0.5) vs. post-1.2% (0.3), P < 0.001<br>• No significant change in overall AVB, mean (SD), pre-3.2% (0.8) vs. post-3.1% (1.0)<br>• Increase in overall CS rates, from 27.9%-30.6% in pre-intervention years to 33.5%-33.9% in post-intervention years (no statistical analyses)

For peer review only

Author	Study design / Year study conducted/ Level of significance / Adjustments	Country / Setting	Population / Number of deliveries	Intervention strategy / Frequency and duration	Effect of intervention (Kirkpatrick levels 3 or 4)
Berglund 2010	Before and after study<br><br>2003-2006<br><br>P< 0.05<br><br>No adjustments	Ukraine<br><br>3 Maternity hospitals (Donetsk, Lutsk, Lviv)	All women (in the 3 sites combined: N=1696 at baseline, N= 6710 intervention period, Total N= 8406)	Development of obstetrical and neonatal protocols, staff training on Effective Perinatal Care (EPC), continuous monitoring and evaluation (with feedback to staff), revision of university curricula to include evidence-based perinatal care, provision of equipment, resuscitation and neonatal care.<br><br>Frequency and duration:<br>Initial (EPC) training: two weeks<br>Follow up EPC training: three days	• Significant decrease in overall AVB in one maternity:<br>Donetsk: from 3.7% to 0% P<0.0001<br>• No change in overall AVB in the other two maternities:<br>Lutsk: from 2.0% to 2.0%<br>Lviv: from 0% to 0%<br>• Significant decrease in CS in two maternities:<br>Donetsk: from 30% to 18.4% P<0.0001<br>Lutsk: from 33% to 12.7% P<0.0001<br>• No change in CS in one maternity:<br>Lviv: from 21.9% to 16.9%<br>• Significant decrease in episiotomies:<br>Donetsk: from 36.8%to 4.0% P<0.0001<br>Lutsk: from 19.4% to 5.7% P<0.0001<br>Lviv: 22.2% to 7.0% P<0.0001<br>• No effect on early neonatal mortality:<br>Donetsk: from 13.6% to 9.5%<br>Lutsk: from 4.6% to 3.0%<br>Lviv: from 6.4% to 0%<br>• Significant decrease in babies needing resuscitation:<br>Donetsk: from 13.1% to 7.4% P = 0.0025<br>Lutsk: from 2.4% to 0.9% P<0.0001<br>Lviv: 4.5% to 1.1% P=0.0003<br>• Significant decrease in NICU admission in two maternities:<br>Lutsk: from 7.3% to 4.4% P= 0.0115<br>Lviv: from 6.4% to 2.4% P = 0.0015<br>• No significant change in NICU admission in one maternity:<br>Donetsk: from 11.2% to 9.4%<br>• Significant decrease in the proportion of hypothermic infants:<br>Donetsk: from 59.5% to 0.6% P<0.0001<br>Lutsk: from 85.8% to 0.3% P<0.0001<br>Lviv: from 77% to 0.7% P<0.0001

For peer review only

**Author**	**Study design / Year study conducted/ Level of significance / Adjustments**	**Country / Setting**	**Population / Number of deliveries**	**Intervention strategy / Frequency and duration**	**Effect of intervention** (Kirkpatrick levels 3 or 4)
Cottrell 2021	Cohort study<br><br>2013-2017<br><br>P< 0.05<br><br>No adjustments	USA<br><br>1 Teaching hospital	Women with prolonged second stage labour (N=2720 at baseline, N= 2800 intervention period Total N=5520)	On-the-job, hands-on AVB training of residents and supervision by attending physicians.<br><br>Frequency and duration not stated.	• Significant increase in forceps deliveries, from 1.8% to 4.0%, P < 0.001<br>• Significant decrease in vacuum deliveries, from 8.7% to 6.5%, P = 0.002.<br>• No significant change in overall AVB, from 10.5% to 10.5%<br>• Increase in overall CS rate from 30.4% to 32.1%, no statistical analysis<br>• 3rd degree laceration: no change in forceps (post vs. pre, RR 0.5, 95% CI 0.24-1.21) and vacuum groups (post vs. pre, RR 0.8, 95% CI 0.37−1.55)<br>• 4th degree laceration: no change in vacuum (post vs. pre, RR 1.0, 95% CI 0.28-3.83); significant decrease in forceps group (post vs. pre, RR 0.1, 95% CI 0.01-0.73)<br>• Overall maternal complication rates: significant decrease in forceps group (post vs. pre, RR 0.40, 95% CI 0.19-0.75); no change in vacuum group (post vs. pre, RR 0.8, 95% CI 0.44-1.51)<br>• Facial laceration: no change in forceps (post vs. pre, RR 0.9, 95% CI 0.08-9.42) and vacuum groups (post vs. pre, RR 0.8, 95% CI 0.19-3.23)<br>• Scalp injury: no change in forceps (post vs. pre, Not estimable) and vacuum groups (post vs. pre, RR 0.5, 95% CI 0.15-1.46)<br>• Cephalohematoma: no change in forceps (post vs. pre, Not estimable) and vacuum groups (post vs. pre, RR 0.8, 95% CI 0.22-2.51)<br>• Overall neonatal complications: no change in forceps (post vs. pre, RR 0.4, 95% CI 0.09-2.09), vacuum groups (RR 0.6, 95% CI 0.31−1.25) and overall (post vs. pre, RR 0.4, 95% CI 0.09-2.09)<br>• No significant change in AVB failure rate (requiring CS) (1.8% requiring CS before the initiative and 1.4% after the initiative)

For peer review only

**Author**	**Study design / Year study conducted/ Level of significance / Adjustments**	**Country / Setting**	**Population / Number of deliveries**	**Intervention strategy / Frequency and duration**	**Effect of intervention** (Kirkpatrick levels 3 or 4)
Dmello 2021	Before and after intervention study<br><br>2011-2019<br><br>P <0.05<br><br>No adjustments	Tanzania<br>22 health facilities	All women<br>(N= 74,792 at baseline, N=666,490 during intervention period-continuous)<br>Total N=741,282	Complex, dynamic, multicomponent, multifaceted intervention to improve the quality of care and survival during pregnancy and childbirth.<br>Components of AVB training: theoretical, simulation, short on-site hands-on training and prolonged on-site supervision (in some settings), provision of VE, quarterly site visits to support on-the-job coaching. At large high-volume health facilities, an on-site mentor was stationed.VE rate was systematically targeted and monitored every month, reported quarterly and annually.<br>**Frequency and duration:**<br>5-day critical BEmONC skills course; 1-day modular course on specific topics addressing gaps detected in perinatal audits; on-site trainer: an experienced nurse or doctor supported by CCBRT was periodically stationed full time for periods from 2 weeks to 1 year in the labour ward of high-volume facilities for on-site coaching and support; and on-the-job coaching (mentors would spend 2–3 days in each of the 22 sites, building skills during routine service provision).	• Significant increase in VE from 0.03% in 2011 to 3.1% in 2019 (RR 1.43; 95% CI 1.41 to 1.43)<br>• Significant increase in overall CS rate from 2.3% in 2011 to 17.6% in 2019 (RR 1.15; 95% CI 1.14 to 1.15)<br>• Significant decrease in MMR: from 154 to 79 per 100 000 live birth (RR 0.92, 95% CI 0.9 to 0.95)<br>• Significant decrease in stillbirth rate from 26 to 21 per 1000 live births (RR 0.96, 95% CI 0.96 to 0.97)<br>• Significant increase in neonatal deaths (sum of deaths among inborn and referred babies) from 5.5 in 2011 to a peak of 14.5) in 2014 and then slightly declined to 12.1 in 2019 (RR 1.05, 95% CI 1.04 to 1.05)

For peer review only

Author	Study design / Year study conducted/ Level of significance / Adjustments	Country / Setting	Population / Number of deliveries	Intervention strategy / Frequency and duration	Effect of intervention (Kirkpatrick levels 3 or 4)
Dominico 2018	Before and after intervention study (ITS)<br><br>2011-2016<br><br>No statistical analyses<br><br>No adjustments	Tanzania<br><br>5 Tertiary hospitals<br>10 Primary health centres	All women<br>(N=2446 at baseline, N=125,908 intervention period<br>Total N=128,354)	Multifaceted intervention comprising decentralization of life-saving services; task-shifting obstetric procedures to other cadres; training that included skill building in vacuum extraction.<br><br>Frequency and duration not stated.	• Increase in vacuum extractions from 0.15% at baseline (no statistical analyses):<br>1st year after intervention: 0.88%<br>2nd year after intervention: 2.11%<br>3rd year after intervention: 1.54%<br>4th year after intervention: 1.26%<br>5th year after intervention: 1.73%<br>• Decrease in CS rates from 11.25% at baseline (no statistical analyses):<br>1st year after intervention: 10.85%<br>2nd year after intervention: 9.42%<br>3rd year after intervention: 9.88%<br>4th year after intervention: 9.80%<br>5th year after intervention: 11.18%
Dumont 2013	Cluster-randomised trial<br><br>2007-2011<br><br>P< 0.05<br><br>OR adjusted for country, hospital type and characteristics, patient characteristics, and birthweight (for perinatal outcomes).	Mali and Senegal<br><br>Referral hospitals (23 in intervention and 23 in control)	All women<br>(N=84,924 at baseline, N=106,243 intervention period<br>Total N=191,167 deliveries)	Multifaceted intervention focused on reducing MM and improving quality of obstetric care comprising onsite training in emergency obstetric care (using Advances in Labour and Risk Management (ALARM course) and update/creation of clinical guidelines, and audit and feedback on maternal deaths,<br><br>Frequency and duration: 6 days once a year (for 2 years)	• Significant increase in overall AVB rate in Senegal from 1.24% to 2.24% in intervention groups (adjusted OR 3.10, 95% CI 1.85 to 5.20)<br>• No significant change in overall AVB rate in Mali from 3.32% to 1.97% in intervention groups, (adjusted OR 0.51, 95% CI 0.16 to 1.59)<br>• Significant decrease in intrapartum CS (adjusted OR 0.87, 95% CI 0.82 to 0.92)<br>• Significant increase in emergency antepartum CS (OR 1.33, 95% CI 1.19 to 1·50), mainly due to pre-eclampsia/eclampsia<br>• Significant decrease in hospital maternal mortality (OR 0.85, 95% CI 0.73 to 0.98)<br>• Marginally significant increase in transfusions (adjusted OR 1.44, 95% CI 0.99 to 2.11)<br>• Significant decrease in neonatal mortality < 24 h (adjusted OR 0.74, 95% CI 0.61 to 0.90)<br>• No significant change in stillbirth rates (OR 1.05, 95% CI 0.91 to 1.22)

For peer review only

**Author**	**Study design / Year study conducted / Level of significance / Adjustments**	**Country / Setting**	**Population / Number of deliveries**	**Intervention strategy / Frequency and duration**	**Effect of intervention** (Kirkpatrick levels 3 or 4)
Geelhoed 2018	Qualitative implementation study with presentation of before and after routine health service data.<br><br>2015-2017<br><br>No statistical analyses<br><br>No adjustments	Mozambique<br><br>Tetu Province Hospitals and primary health facilities with > 100 births per 3-months-period; this amounted to 52-82 out of a total of some 120 health facilities	All women (N= 88,000 at baseline, N=265,061 intervention period Total N = 353,061 births)	Multifaceted intervention (training on the management of prolonged labour including vacuum extraction; accreditation in emergency obstetric care; monitoring and evaluation of routine data from all maternities; audit and feedback of CS. Clinicians at the provincial hospital were assigned to provide in-service training to any MCH nurse or doctor wishing to strengthen their capabilities. Key components of AVD module:<br>1) didactic training<br>2) simulation training<br>3) Hands-on training<br>4) Audit & feedack<br><br>Duration:<br>VE training: 1 week<br>QI strategy: 3 years	• Increase in vacuum deliveries from 0.2% at baseline (no statistical analyses):<br>1st yr after intervention: 1.0%<br>2nd yr after intervention: 2.3%<br>3rd yr after intervention: 2.2%<br>• Decrease in overall CS rate from baseline rate of 2.7% (no statistical analyses):<br>1st yr after intervention: 2.1%<br>2nd yr after intervention: 2.1%<br>3rd yr after intervention: 1.8%<br><br>• Decrease in institutional maternal mortality rate of 126/100,000 births at baseline (no statistical analyses):<br>1st year after intervention: 49/100,000<br>2nd year after intervention:48/100,000<br>3rd year after intervention: 44/100,000<br><br>• Decrease in institutional stillbirth rate from baseline of 17.7/1000 newborns (no statistical analyses):<br>1st year: 14.9/1000<br>2nd year: 12.4/1000<br>3rd year: 12.2/1000
Gulmezoglu 2006	Cluster-randomised trial<br><br>2001-2002<br><br>P <0.05<br><br>No multivariable regressions. End rates adjusted by baseline rates	Mexico, Thailand<br><br>**Mexico:** 22 health facilities not associated directly with a university or other academic or research department<br><br>**Thailand:** 18 primary and secondary health facilities	**Mexico:** All women (N=19,592 at baseline, N=22,236 intervention Total N=41,828)<br><br>**Thailand:** All women (N=17,655 at baseline, N=17,998 intervention Total N=35,653)	Multifaceted educational intervention to promote the use of the WHO Reproductive Health Library<br><br>Frequency and duration: Three 1-day workshops over a period of 6 months. Other components were continuous over the time of the trial	**Mexico:** No significant changes in intervention or control groups from baseline to adjusted end-of-study VE rates (intervention: 0.6% to 0.2% and control: 0.4% to 0.1%). Difference in adjusted end-of-study VE rate: 0.1, 95% CI -0.1 to 0.2<br><br>**Thailand:** No significant changes in intervention or control groups from baseline to adjusted end-of-study VE rates (intervention: 7.5% to 7.7% and control: 6.3% to 7.7%). Difference in adjusted end-of-study VE rate: 0.0, 95% CI -1.5 to 1.4

For peer review only

**Author**	**Study design / Year study conducted / Level of significance / Adjustments**	**Country / Setting**	**Population / Number of deliveries**	**Intervention strategy / Frequency and duration**	**Effect of intervention** (Kirkpatrick levels 3 or 4)
Mogilevkina 2022	Non-randomized controlled trial<br><br>2004-2009<br>Baseline: 2004-2005<br><br>Intervention: 2006-2007 (not analyzed)<br><br>After intervention: 2008-2009<br><br>P<0.05<br><br>No adjustments	Ukraine<br>Intervention: 28 city or district hospitals<br><br>Control: 36 city or district hospitals	All women<br><br>Intervention hospitals:<br>2004-2005: 47,838<br>2008-2009: 61,116<br><br>Control hospitals:<br>2004-2005: 38,032<br>2008-2009: 42,866	Multifaceted EMOC intervention using the Advances in Labour and Risk Management (ALARM) International Program (AIP). One 5-day course focused on the five main causes of maternal mortality. AVB training is one of the sessions.	• Significant increase in VE from 0.10% to 0.69% in intervention group (OR: 2.86; 95% CI: 1.80-4.57, P<0.001)*<br>• Significant decrease in Forceps rates from 0.17% to 0.08% in intervention group (OR: 1.80; 95% CI: 1.00-3.25, P= 0.041)**<br>• Significant increase in overall CS rate from 13.12% to 15.93% in intervention group (OR: 1.11; 95%CI: 1.06-1.17, P<0.001)*<br>• MM: non-significant change<br>• PPH > 1000 ml: non-significant change<br>• Post-partum hysterectomy: non-significant change<br>• Blood transfusion: significant decrease in intervention group from 2.11% to 0.48% (OR: 0.56; 95% CI: 0.48-0.65, P<0.001)*<br>• Plasma transfusions: significant decrease in intervention group from 4.56% to 2.28% (OR: 0.70; 95% CI: 0.63-0.78, P<0.001)*<br>• Uterus explorations: significant decrease in intervention group from 8.32% to 4.14% (OR: 0.64; 95% CI: 0.59-0.69, P<0.001)*<br><br>*Difference in difference estimator comparing the change in the rate of events or outcomes in the control versus the intervention group<br>**The decrease in forceps rate was larger in the control than in the intervention group.

For peer review only

Author	Study design / Year study conducted/ Level of significance / Adjustments	Country / Setting	Population / Number of deliveries	Intervention strategy / Frequency and duration	Effect of intervention (Kirkpatrick levels 3 or 4)
Nolens 2016	Before and after intervention study<br><br>2012-2014<br><br>P<0.05<br><br>No adjustments	Uganda<br><br>1 Teaching hospital	Women with "medium to high risk pregnancies" (N=12,143 at baseline N= 34,894 intervention period Total N=47,037)	Development of a vacuum extraction guideline, supply of vacuum extraction equipment, training of staff in didactic and simulation sessions, and on the job supervision (for 1 week, after formal training sessions) and continued continuous supervision on labour ward for 4 months during which most residents had several labour ward shift<br><br>Frequency and duration: 4 months initial training accompanied by yearly refresher (theory and simulation training session)	• Significant increase in vacuum deliveries from 0.6% to 2.1%, P<0.01<br>• Significant increase in overall CS rate from 28.2% to 30.2%, P< 0.01<br>• No significant change in CS due to obstructed labour from 6.0% to 6.0%<br>• Significant decrease in women with ruptured uterus from 1.1% to 0.8%, P< 0.01<br>• No significant change in overall maternal deaths from 0.8% to 0.7%<br>• No significant change in maternal deaths due to intrapartum complications from 0.3% to 0.2%<br>• No significant change in maternal ICU admission from 1.9% to 1.8%<br>• Significant decrease in total perinatal deaths (stillbirths + neonatal deaths during admission) from 9.2% to 8.4%, P=0.02<br>• Significant decrease in intrapartum stillbirths from 3.4% to 2.6% , P< 0.01<br>• Significant increase in term admissions to NICU from 8.7% to 10.0%, , P< 0.01<br>• No significant change in term neonatal deaths from 1.3% to 1.4%
Skinner 2017	Retrospective cohort study (ITS)<br><br>2005-2014<br><br>P<0.05<br><br>No adjustments	Australia<br><br>1 Teaching hospital	All women excluding those with preterm or anomalous births, or who had CS without prior instrumental attempt (Total N=72,490 births)	Residency training program: formalized lecture series and mannequin simulation instrumental birth training and; mandatory instrumental birth credentialing for all obstetric residents	• Significant increase in forceps births from 4.8% last year pre-intervention to 9.8%-last year post-intervention (average annual rate (β) 1.5 per 100 births, 95% CI 1.03 to 1.96 per 100 births)<br>• Significant decrease in vacuum births from 8.0% last year pre-intervention to 6.0% last year post-intervention (β -1.43, 95% CI -2.5 to -0.37)<br>• Significant decrease in postpartum haemorrhage (β -1.3, 95% CI -2.07 to -0.49)<br>• No significant change in 3rd-4th degree tears (β -1.04, 95% CI -3.1 to 1.00)<br>• No significant change in composite neonatal morbiditya (β -0.18, 95% CI -0.38 to 0.02)

For peer review only

Author	Study design / Year study conducted/ Level of significance / Adjustments	Country / Setting	Population / Number of deliveries	Intervention strategy / Frequency and duration	• **Effect of intervention** (Kirkpatrick levels 3 or 4)
Solt 2011	Retrospective cohort (pre-post intervention)<br><br>Year study conducted not stated<br><br>P<0.05<br><br>No adjustments	USA<br><br>1 Teaching hospital	All women<br>(N=3481 at baseline<br>N=4338 intervention period<br>Total N=7819)	Day time coverage of labour and delivery by a specially recruited senior generalist obstetrician with 35 years' clinical experience<br><br>Duration: 2 years	• Significant increase in forceps deliveries, from 4.9% to 7.8%, P < 0001<br>• Significant decrease in vacuum deliveries, from 6.4% to 2.9%, P < 0001<br>• No significant change in Overall AVB: from 11.3% to 10.6%<br>• No significant change in Overall CS, from 25.5% to 27.3%<br>• No significant change in<br>-3rd-4th degree tears, from 3.6% to 3.1%<br>-5 min Apgar < 7, from 1.9% to 2.4%<br>-Birth injuries (fracture, laceration, palsy), from 0.2% to 0.3%
Sorensen 2010	Prospective intervention study (before and after training evaluation)<br><br>2008<br><br>P<0.05<br><br>No adjustments	Tanzania<br><br>1 Teaching hospital	All women giving birth to a neonate ≥ 1000 g<br>(N= 558 at baseline<br>N= 550 intervention period<br>Total N=1108)	2-day Advanced Life Support in Obstetrics (ALSO) provider course comprising lectures, workshops, and case discussions on the major topics of emergency obstetric and neonatal care (EmONC). In the workstation on vacuum delivery, hands-on skills were practiced using pelvic and fetal mannequins and the Kiwi OmniCup	• No change in vacuum delivery rates (0% to 0%)<br>• No significant change in CS before active labor, from 2.0% to 1.8%<br>• No significant change in emergency CS, from 6.9% to 6.3%<br>• No significant change in CS for prolonged labor, from 5.1% to 4.4%<br>• Significant decrease in death of liveborn with Apgar ≥ 4, from 1.1% to 0%, P=0.03<br>• No significant change in:<br>-Neonatal deaths, from 1.2% to 0.4%<br>-Stillbirths, from 1.7% to 2.5%<br>-Neonatal resuscitation, from 3.0% to 2.4%<br>• -1-min Apgar score < 7 from 6.4% to 6.9%

For peer review only

**Author**	**Study design / Year study conducted/ Level of significance / Adjustments**	**Country / Setting**	**Population / Number of deliveries**	**Intervention strategy / Frequency and duration**	**Effect of intervention** (Kirkpatrick levels 3 or 4)
Takeda 2018	Before and after study, ITS<br><br>2016<br><br>No statistical analyses<br>No adjustments	Japan<br><br>1 Primary health facility	All women<br>(N=4607 at baseline,<br>N= 650 intervention period<br>Total N=5257)	Forceps delivery simulation training comprising lectures with forceps delivery videos, demonstrations, and supervised simulation forceps deliveries	• Forceps rate increased from baseline 0%-2.0% in the 10 baseline pre-intervention years to 4.3% in the post-intervention year (no statistical analyses)<br>• Vacuum rates decreased from 1.2-13.3% in the 10 baseline pre-intervention years to 2.3% in post-intervention year (no statistical analyses)<br>• Overall CS rates decreased from 7.3%-25.6% in the 10 baseline pre-intervention years to 22.2% in post-intervention year (no statistical analyses)

AVB: assisted vaginal birth. CI: confidence interval. EmONC: emergency obstetric and neonatal care. ITS: interrupted times series. NICU: neonatal intensive care unit. OR: odds ratio. RR: relative risk. SD: standard deviation

a. Composite neonatal morbidity: a composite of low Apgar at 5 min (<7), neonatal intensive care unit or special care nursery admission, cephalohematoma, lacerations or abrasions, bruising, intraventricular hemorrhage, brachial plexus injury, fractured clavicle, convulsions, phototherapy, neonatal feeding problems, and respiratory distress syndrome.

For peer review only

Supplementary file 4. Assessment of the quality of 16 included studies

Risk of bias of 13 non-randomized intervention studies[a]

Study	Bias due to confounding	Bias in selection of participants into the study	Bias in classification of interventions	Bias due to deviations from intended interventions	Bias due to missing data	Bias in measurement of outcomes	Bias in selection of the result	Overall bias [b]
Bardos 2017	Low	Low	Low	Low	Low	Low	Low	Low
Becker 2020	Moderate	Low	Low	Low	Low	Low	Low	Moderate
Berglund 2010	Moderate	Low	Low	Low	Low	Low	Low	Moderate
Cottrell 2021	Serious	Serious	Low	Low	Low	Low	Low	Serious
Dmello 2021	Moderate	Low	Low	Low	Low	Low	Low	Moderate
Dominico 2018	Serious	Low	Low	Low	Low	Low	Low	Serious
Geelhoed 2018	Serious	Low	Low	Low	Low	Low	Low	Serious
Mogilevkina 2022	Moderate	Low	Low	Low	Low	Low	Low	Moderate
Nolens 2016	Serious	Low	Low	Low	Low	Low	Low	Moderate
Skinner 2017	Low	Moderate	Low	Low	Low	Low	Low	Moderate
Solt 2011	Moderate	Low	Low	Low	Low	Low	Low	Moderate
Sorensen 2010	Moderate	Low	Low	Low	Low	Low	Low	Moderate
Takeda 2018 [c]	-	-	-	-	-	-	-	-

[a] Based on Risk of Bias for Non-Randomised Studies of Interventions (ROBINS-I) tool.
[b] Interpretation of overall risk of bias.
[c]Takeda 2018:Risk of bias not assessed due to insufficient information.

Low risk of bias: The study is comparable to a well performed randomised trial.
Moderate risk of bias: The study provides sound evidence for a nonrandomized study but cannot be considered comparable to a well performed randomised trial.
Serious risk of bias: The study has some important problems.
Critical risk of bias: The study is too problematic to provide any useful evidence and should not be included in any synthesis.
No information: No information on which to base a judgement about risk of bias.

For peer review only

Risk of bias of 3 randomized trials[a]

Study	Random sequence generation (selection bias)	Allocation concealment (selection bias)	Baseline outcome measurements	Baseline characteristics	Blinding of participants and personnel (performance bias)	Blinding of outcome assessment (detection bias)	Contamination	Incomplete outcome data (attrition bias)	Selective reporting (reporting bias)	Overall bias[b]
Ameh 2014	Low	Low	Low	Low	Low	Low	Low	Low	Low	Low
Dumont 2013	Low	Low	Low	Low	Low	Low	Low	Low	Low	Low
Gulmezoglu 2006	Low	Low	Low	Low	Low	Low	Low	Low	Low	Low

[a] Based on the Cochrane EPOC Risk of bias criteria for randomised trials.
[b] Interpretation of overall risk of bias

Low risk of bias: Low risk of bias for key quality domains (i.e. allocation sequence generation and concealment)
High risk of bias: High risk of bias for one or more of the key domains
Unclear risk of bias: Unclear risk of bias for one or more key domains

For peer review only

Supplementary file 5. Baseline rates of assisted vaginal births and cesarean deliveries in studies included in the systematic review

Baseline rates[a]	N of studies	References
VE rates (N=14 studies, 15 data sets[b])		
range	0% to 8.7%	
< 1%	8	Ameh 2014 (Kenya), Dmello 2021 (Tanzania), Dominico 2018 (Tanzania), Geelhoed 2018 (Mozambique), Gulmezoglu 2006 (Mexico[c]), Mogilevkina 2022 (Ukraine), Nolens 2016 (Uganda), Sorensen 2010 (Tanzania)
1- 4 %	3	Takeda 2018 (Japan), Becker 2020 (USA), Bardos 2017 (USA)
> 4%	4	Solt 2011 (USA), Gulmezoglu 2006 (Thailand[c]), Skinner 2017 (Australia), Cottrell 2021 (USA)
Forceps rates (N=7 studies)		
range	0% to 5.2%	
< 1%	3	Bardos 2017 (USA), Mogilevkina 2022 (Ukraine), Takeda 2018 (Japan)
1-3%	2	Becker 2020 (USA), Cottrell 2021 (USA)
>3%	2	Skinner 2017 (Australia), Solt 2011 (USA)
Overall AVB rates (N=5 studies, 6 data sets[d])		
range	1.8% to 11.3%	
1-3%	3	Dumont 2013 (Senegal and Mali[e]) , Berglund 2010[f] (Ukraine)
>3 to 4.9%	1	Becker 2020 (USA)
5 to 10%	0	
>10%	2	Cottrell 2021 (USA), Solt 2011 (USA)
Overall CS rates (N=12 studies)		
range	2.6% to 30.6%	
< 10 %	3	Dmello 2021 (Tanzania), Geelhoed 2018 (Mozambique), Mogilevkina 2022[g] (Ukraine)
11-20%	1	Dominico 2018 (Tanzania)
21-30%	6	Ameh 2014 (Kenya), Solt 2011(USA), Takeda 2018 (Japan), Bardos 2017 (USA), Nolens 2016 (Uganda), Berglund 2010[f] (Ukraine)
>30%	2	Cottrell 2021 (USA), Becker 2020 (USA)

[a.] In studies with >1 time point, we present the lowest AVB rate and the highest CS rate before intervention. [b] One study (Gulmezoglu 2006) conducted in two countries (Mexico & Thailand) analyzed separately. [c] Gulmezoglu 2006 (randomized trial): rates in control groups. [d] One study (Dumont 2013) conducted in two countries (Senegal & Mali). [e] Dumont 2013 (cluster randomized trial): rates in control groups. [f] Berglund 2010: combined rates from three sites. [g] Mogilevkina 2022 (non-randomized trial): rate in control group.
AVB: Assisted vaginal birth; CS: Cesarean section; VE: Vacuum extraction

For peer review only

Supplementary file 6. Details of interventions in 16 included studies

Study	Details of intervention strategy	Health provider included in intervention strategy / Number (N)	Mode of delivery of intervention strategy	Frequency and duration of intervention strategy	AVB instrument used in strategy
Ameh 2014	Multifaceted intervention:<br>1. **EmONC training**<br>• Training using adapted version of the Life Saving Skills-Emergency Obstetric and Newborn Care (LSS-EONC) training package, a competency-based training developed specifically for low resource settings. The course covers a list of 10 topics, including AVB. A cross cutting theme throughout the training is effective communication and teamwork. In the adaptation, forceps training was excluded and only VE was taught for AVB.<br>• **Multidisciplinary training approach:** midwives, medical doctors and clinical officers are trained together by multidisciplinary teams of experienced specialist obstetricians, midwives and anaesthetists (UK based volunteers and in-country volunteers).<br>• **On-site training:** the training is delivered as close as possible to the health care providers' place of service delivery.	Obstetricians, medical doctors, clinical officers, midwives<br><br>Overall, 400 maternity care workers were trained.<br><br>In each of the 10 hospitals, 72-100% of relevant staff were trained.	Face-to-face interactive learning sessions comprising of short lectures, simulation, and hands-on sessions using low fidelity obstetric, newborn and resuscitation manikins	3 days	Vacuum

For peer review only

	**Details of intervention strategy**				
Ameh 2014, continued	2. **Local supportive supervision:** local master trainers were trained (1-day "train the trainer" course) and mentored during another EmOC training course - covering the principles of supportive supervision, tools for supportive supervision, developing and implementing action plans from the process of supportive supervision. These local master trainers (4-6 per hospital) were usually obstetricians, senior midwives, clinical based on their knowledge and potential to teach/mentor others on the job. Local supportive supervisors were expected to provide on the job support to other colleagues, facilitating behaviour change after the training.<br><br>3. **Essential equipment:** all hospitals were provided with essential equipment including 4 reusable Kiwi omnicups per hospital.				

For peer review only

Study	Details of intervention strategy	Health provider included in intervention strategy / Number (N)	Mode of delivery of intervention strategy	Frequency and duration of intervention strategy	AVB instrument used in strategy
Bardos 2017	Senior obstetricians (more than 20 years experience) teaching and supervising of residents on forceps deliveries.<br>• One to two senior obstetricians covered labor and delivery at least 5 days a week.<br>• Two senior obstetricians supervised resident deliveries during daytime (7:00 am to 7:00 pm); one supervised resident deliveries during nights and weekends (with a second obstetrician available as backup in the event of an emergency or increased volume).	Residents<br><br>N not stated	On-site, face-to-face teaching and supervision of forceps deliveries	Not stated	Forceps
Becker 2020	Assisted vaginal birth (AVB) educational curriculum consisting of didactics and simulation. The curriculum comprised twice-yearly workshops for residents (excluding first year residents). A typical workshop would have 10 to 12 participants.<br><br>Components of the workshop<br>• 28-question pretest to evaluate basic AVB knowledge; 1-hour didactic on the history, indications, safe use, and risks of forceps and vacuums; and<br>• 1-hour hands-on simulation where residents are taught how to properly execute an AVB (forceps and vacuum) using a standard maternal pelvis model and dummy fetus.<br><br>The didactic portion of the workshop is delivered to the entire group of residents. The hands-on simulation is performed in groups of three to four residents paired with one to two faculty members. After the session, residents complete a 50-question posttest, with a passing mark of 70%.	Residents<br><br>N not stated	Face-to-face lectures, hands-on simulation using a standard maternal pelvis model and dummy fetus	One 2-hour workshop offered every 6 months<br><br>Duration of hands-on training / supervision not stated	Forceps, Vacuum

For peer review only

Study	Details of intervention strategy	Health provider included in intervention strategy / Number (N)	Mode of delivery of intervention strategy	Frequency and duration of intervention strategy	AVB instrument used in strategy
Berglund 2010	Multifaceted intervention comprising:<br>• Continuous monitoring and evaluation system with feedback to the staff, improving medical services and counseling with special attention to resuscitation, breast feeding, and prevention of infections and to strengthen continuity of care.<br>• Development and dissemination of national, evidence-based obstetrical and neonatal protocols.<br>• Staff training on evidence-based guideline. The Russian version of the training material 'Effective Perinatal Care' (EPC), developed by WHO Regional Office for Europe was used. The material is a comprehensive, didactic, interactive two-week training package including clinical activities and a team approach to maternity care<br>• Revision of university curricula to include evidence-based perinatal care.<br>• Participating maternities were provided with basic equipment for resuscitation and neonatal care and tools for the delivery room to encourage change of position during labor and more family-oriented care.<br><br>After evaluation of the progress in the maternities by trainers, additional practical training was provided: reinforcement training in neonatal care (nine days), reinforcement in modern perinatal technology (nine days) and care in second stage in preterm delivery (one day).	Obstetricians, neonatologists, midwives, pediatric nurses, pediatricians, anesthesiologists	Face-to-face, practical clinical scenarios,	Initial Effective Perinatal Care (EPC) training: 2 weeks<br><br>Follow up EPC training: 3 days	Vacuum

For peer review only

Study	Details of intervention strategy	Health provider included in intervention strategy / Number (N)	Mode of delivery of intervention strategy	Frequency and duration of intervention strategy	AVB instrument used in strategy
Cottrell 2021	On-the-job, hands-on AVB training of residents and supervision by attending physicians.	Residents<br><br>N not stated	Hands-on training	Not stated	Forceps, Vacuum
Dmello 2021	Multiple intervention components addressed gaps across the maternal and perinatal continuum of care (training, infrastructure, routine data quality strengthening and utilization). Multiple interventions were implemented to improve the quality of Basic Emergency Obstetric Care, towards addressing maternal and perinatal mortality; specifically targetted were the EMOC signal functions that include vacuum extraction.<br><br>Components:<br>• 2-week BEmONC national training, facilitated by national trainers;<br>• 5-day critical BEmONC skills course;<br>• 1-day modular course on specific topics addressing gaps detected in perinatal audits;<br>• on-site trainer: an experienced nurse or doctor supported by CCBRT was periodically stationed full time for periods from 2 weeks to 1 year in the labour ward of high-volume facilities for on-site coaching and support;<br>• on-the-job coaching (mentors would spend 2–3 days in each of the 22 sites, building skills during routine service provision).<br>Provision of vacuum extractors was a critical component of the intervention.	Doctors and midwives.<br><br>N=1540	Face to face, videos, simulation training, group discussions, hands-on coaching	Duration of each intervention component: in 2nd column<br><br>Frequency: NI	Vacuum

Study	Details of intervention strategy	Health provider included in intervention strategy / Number (N)	Mode of delivery of intervention strategy	Frequency and duration of intervention strategy	AVB instrument used in strategy
Dominico 2018	Strategy for the 10 primary health care centers<br>• Decentralization of life-saving services from district or regional hospitals to primary health care centres. They were upgraded to provide comprehensive emergency obstetric and newborn care (C-EmONC) (i.e. they were staffed and equipped to provide obstetric surgery, blood transfusion, and basis EmONC signal functions).<br>• Task-shifting obstetric procedures from doctors, to advanced level associate clinicians or assistant medical officers (AMOs), and anaesthesia to associate clinicians or nurse anaesthetists and clinical officers.<br>Strategy for the 5 hospitals<br>• Three months clinical training (that included skill building in vacuum extraction (VE) at the two largest medical training centres in the country.<br>• Training was followed by week-long Continuing Medical Education (CME) sessions that focused on specific topics that were identified as requiring further confidence- and skills-building, such as VE; training on mannequins.<br>• Training in VE also included how to manage complications such as postpartum haemorrhage and perineal lacerations.<br>• Human resources support, clinical audit, on-going mentoring supervision.<br>• On-the-job coaching and hands-on mentoring by experts during monthly on-site visits.<br>For all institutions:<br>• Weekly conferences allowing staff to discuss specific cases and networking with others to	Assistant medical officers, nurse-midwives<br><br>N = 80	Groups of 9-14 people (face-to-face). Training was competency-based<br><br>Trainees used anatomical models for practice. They would observe procedures being performed but had only few opportunities to perform a VE on a patient<br><br>eLearning sessions were developed.<br>In the case of VE, the trainers adopted WHO's video on VE and translated it into Swahili	Not stated	Vacuum

For peer review only

	**Details of intervention strategy**				
Dominico 2018, continued	For all institutions:<br>• Weekly conferences allowing staff to discuss specific cases and networking with others to facilitate specialist to be "on-call" so emergency consultations were possible day and night.<br>• A WhatApp group was created.<br>• Audit and feedback about procedures and outcomes				

For peer review only

Study	Details of intervention strategy	Health provider included in intervention strategy / Number (N)	Mode of delivery of intervention strategy	Frequency and duration of intervention strategy	AVB instrument used in strategy
Dumont 2013	Multicomponent intervention including:<br><br>6-day training workshop provided by certified instructors in each hospital to one doctor and one midwife (train the trainer) who were responsible for maternity services.<br>• Training used ALARM (Advances in Labour and Risk Management) international course.<br>• The session consisted of 3 days of training in best practices in emergency obstetric care, 1 day of training in maternal death reviews, 1 day of awareness training related to economic, socio cultural, and ethical barriers (including sexual and reproductive rights), and 1 day of training in adult education methods.<br>• At the end of the session, a normative evaluation was done.<br>• The trainers had to attend two recertification sessions (once a year) to verify their knowledge, update them on the clinical content and process of maternal death audits, discuss their roles, share their experiences, and confirm their capacity to provide leadership in their clinical settings.<br><br>Audit and feedback on maternal deaths.<br>• A multidisciplinary audit committee including physicians, midwives, nurses, administrators was created in each participating site and trained in the process of undertaking maternal death reviews.	Doctors and midwives<br><br>N = 46	Face-to-face	6 days once a year (for 2 years)	Forceps, Vacuum

For peer review only

	**Details of intervention strategy**				
Dumont 2013, continued	Audit and feedback on maternal deaths (continued)<br>• The audit cycle and onsite training were then launched in each intervention site with the support of external facilitators (certified instructors) during their quarterly educational outreach visits.<br><br>If needed, local trainers who took part in the initial training workshop developed **new clinical guidelines** or updated existing guidelines according to best practices for emergency obstetric care.				

For peer review only

Study	Details of intervention strategy	Health provider included in intervention strategy / Number (N)	Mode of delivery of intervention strategy	Frequency and duration of intervention strategy	AVB instrument used in strategy
Geelhoed 2018	Four complementary interventions:<br><br>1. One-week training on the management of prolonged labour, including vacuum extraction and craniotomy, developed and facilitated by a professor considered an expert on obstetric care in low-income countries both internationally and in Mozambique. Clinicians at the provincial hospital were assigned to provide in-service training to any MCH nurse or doctor wishing to strengthen their capabilities<br><br>2. Strengthening of the quarterly emergency obstetric care accreditation process<br><br>3. Monthly audit of clinical files of all cases of caesarean section in the provincial hospital (a large majority of all caesarean sections in the province) with specific feedback to clinicians involved, copied to relevant provincial managers<br><br>4. Strict monitoring and evaluation of routine data from all maternities, with specific feedback to district health directors and doctors, copied to all and to relevant provincial manager<br><br>Sharing of capabilities between staff was encouraged. Prizes and public recognition of successful hospitals.	Provincial level: nurse-midwives, general doctors, one consultant OB-GYN specialist<br><br>District level: nurse-midwives<br><br>335,971 births (2014 to 2017)	Face-to-face (1-week training including VE)	1-week training including VE (provided only once at provincial level with a consultant OB-GYN / facilitator, and repeated in a simplified and shortened format in most of the districts in the province in the following months)<br><br>QI strategy: 3 years	Vacuum

For peer review only

Study	Details of intervention strategy	Health provider included in intervention strategy / Number (N)	Mode of delivery of intervention strategy	Frequency and duration of intervention strategy	AVB instrument used in strategy
Gulmezoglu 2006	Multifaceted intervention addressing perceived barriers to evidence-based practice.<br>• Meeting with hospital directors/heads of obstetrics and gynaecology departments before intervention for their consent and support (to ensure organisational buy in).<br>• Provision of the WHO Reproductive Health Library (RHL), computers and printer in an accessible part of the labour ward/department (to ensure easy access to the RHL).<br>• Identification of a hospital RHL coordinator from staff (to help health care workers to use RHL and to maintain links with the research team in case of problems).<br>• RHL information materials (poster, short printouts) to promote awareness in health care workers.<br>• A series of interactive workshop delivered by a specialist (to teach principles of evidence-based practice and use of RHL). Only didactic material.	Doctors (obstetricians, neonatologist, anaesthetist), midwives, interns and students<br><br>N not stated	Face-to-face workshops	Three 1-day workshops over a period of 6 months. Other components were continuous over the time of the trial	Vacuum

For peer review only

Study	Details of intervention strategy	Health provider included in intervention strategy / Number (N)	Mode of delivery of intervention strategy	Frequency and duration of intervention strategy	AVB instrument used in strategy
Mogilevkina 2022	Staff of participating hospitals were trained using the Advances in Labour and Risk Management (ALARM) International Program (AIP). The course is designed to motivate and encourage health professionals to improve the delivery of emergency obstetric care in LMIC.<br>• It consists of a 5-day course which is based on adult learning principles that promote a collaborative, multidisciplinary approach of working and learning together. It focuses on the five main causes of maternal mortality.<br>• It includes interactive plenary sessions and hands-on skill workshops with obstetrical mannequins	Total = 577 professionals<br>-342 OB-GYNs<br>-235 midwives<br><br>They worked in 28 hospitals. Coverage of staff training was 65.5%(range 28.6% to 100%).	Face-to-face<br><br>Interactive plenary sessions<br><br>Hands-on skill workshops with obstetrical mannequins	On 5-day course (AVB is one session of the whole course; no details on duration of this session)	VE and Forceps
Nolens 2016	Development of a vacuum extraction **guideline, supply of vacuum extraction** equipment, **training** of staff, and **on the job supervision**.<br><br>• Standard operating procedures (SOP) for the use of vacuum extraction and sterilization of Kiwi vacuum extractors were developed.<br>• Group training of residents in the hospital, 1 week before their rotation in labor and delivery ward.<br>• Training consisted of discussion of the SOP on vacuum extraction and sterilization, watching the WHO RHL video on vacuum extraction and skills training on mannequins.<br>• On the job supervision in the week following training.<br>• Continuous supervision on labour ward for 4 months during which most residents had several labour ward shift	Residents<br><br>N = 45	Face-to-face, small groups (4-6 residents), on site<br><br>Group presentation of the guidelines and sterilization SOP<br><br>Video (WHO) on VE and discussion<br><br>Skills training on mannequins<br><br>Continuous onsite supervision for 4 months during labour ward shifts	4 months initial training accompanied by yearly theory and a yearly skills training session	Vacuum (Kiwi OmniCup)

For peer review only

Nolens 2016, continued	• After completion of this program with a duration of 4 months, training continued according to the existing curriculum complemented with the new SOP on vacuum extraction.<br>• The existing curriculum consisted of a yearly theory session and a yearly skills training session on mannequins per year group (there were 3 year groups: year 1, 2 and 3).<br>• Both the previously trained providers (residents in year 1, 2 and 3) and the newly arrived (year 1) residents were trained according to the existing curriculum after the initial training period of four months.<br>• Yearly refresher course (theoretical and skills-mannequin training) was provided to all residents and final-year medical students.				

For peer review only - http://bmjopen.bmj.com/site/about/guidelines.xhtml

Study	Details of intervention strategy	Health provider included in intervention strategy / Number (N)	Mode of delivery of intervention strategy	Frequency and duration of intervention strategy	AVB instrument used in strategy
Skinner 2017	Residency training program: policy requiring residents to develop competency in outlet, low, mid-cavity, and rotational instrumental birth.<br>• Before 2010, resident teaching in forceps and vacuum births was predominantly case based with additional informal lectures and mannequin simulation.<br>• In 2010, formalized lecture series and mannequin simulation training were implemented preceding mandatory instrumental credentialing for all obstetric residents.<br>• Credentialing required residents to be directly supervised by senior obstetricians in human instrumental birth until assessed as competent for unsupervised practice.<br>• Residents could only be credentialed in vacuum birth after being first credentialed in forceps birth.<br>• All residents were required to meet with training supervisors at 3-monthly intervals to review credentialing documents and implement remedial pathways if credentialing was not achieved in an appropriate timeframe.	Residents<br><br>N not stated	Lecture series, mannequin simulation, performance of supervised human instrumental birth	5 years (ongoing post-training intervention)	Forceps, Vacuum

For peer review only

Study	Details of intervention strategy	Health provider included in intervention strategy / Number (N)	Mode of delivery of intervention strategy	Frequency and duration of intervention strategy	AVB instrument used in strategy
Solt 2011	Day time coverage of labour and delivery (L&D) by a specially recruited senior generalist obstetrician with 35 years' clinical experience.<br>• The obstetrician was involved in teaching all aspects of obstetrical management and appropriate use of instrumental assisted delivery.<br>• The attending physician worked as the L&D laborist 4 days (40 hours) in a week and performed night call approximately 2 to 3 times per month.	Residents<br><br>N not stated	On-site labour and delivery coverage and resident training by obstetrician	2 years	Forceps (with predominance of Simpson or Luikart-Simpson forceps)<br><br>Vacuum

For peer review only

Study	Details of intervention strategy	Health provider included in intervention strategy / Number (N)	Mode of delivery of intervention strategy	Frequency and duration of intervention strategy	AVB instrument used in strategy
Sorensen 2010	2-day Advanced Life Support in Obstetrics (ALSO) provider course.<br>• The course consisted of lectures, workshops and case discussions on the major topics of emergency obstetric and neonatal care (EmONC).<br>• Each workstation lasted 75 minutes and case discussion 30-45 minutes.<br>• Intrapartum monitoring was taught in small groups discussing 5 partograph-based case stories with focus on assessment of fetal heart rate and progress of labor.<br>• Artificial rupture of membranes and oxytocin augmentation were emphasized as actions to perform before considering caesarean delivery.<br>• In the workstation on vacuum delivery, hands-on skills were practiced using pelvic and fetal mannequins and the “Kiwi OmniCup,” a hard plastic cup with a pump integrated in the handle.<br>• Neonatal resuscitation was covered by both a lecture and a workshop.	Doctors, assistant medical officers, nurse-midwives<br><br>N = 26	Face-to-face, workshop, lectures, simulations with mannequins	2 days	Vacuum

For peer review only

Study	Details of intervention strategy	Health provider included in intervention strategy / Number (N)	Mode of delivery of intervention strategy	Frequency and duration of intervention strategy	AVB instrument used in strategy
Takeda 2018	Forceps delivery simulation training.<br>• Consisting of a lecture with forceps delivery videos, a demonstration and simulation training experience.<br>• The lecture is about pelvic examination for precise assessment of fetal head descent based on trapezoidal fetal station and is supplemented by forceps delivery videos.<br>• Then, forceps delivery is demonstrated by instructors, using a pelvic model and fetal doll.<br>• Finally, trainees attempt a simulated forceps delivery under the supervision of a specialist.	Obstetrician<br><br>N = 1	Face-to-face lecture with forceps delivery videos, a demonstration, and practicing in simulation pelvic model	Not stated	Utokyo Naegele Forceps

For peer review only

Supplementary file 7. Types of outcomes reported in the 16 included studies

Outcome	N studies (Total= 16)	References
OVD rates		
VE rates	14	Ameh 2014, Bardos 2017, Becker 2020, Cottrell 2021, Dmello 2021, Dominico 2018, Geelhoed 2018, Gulmezoglu 2016, Mogilevkina 2022, Nolens 2016, Skinner 2017, Solt 2011, Sorensen 2010, Takeda 2018
Forceps rates	7	Bardos 2017, Becker 2020, Cottrell 2021, Mogilevkina 2022, Skinner 2017, Solt 2011, Takeda 2018
Overall OVD rates	5	Berglund 2010, Becker 2020, Cottrell 2021, Dumont 2013, Solt 2011
Caesarean rates		
No information	2	Gulmezoglu 2016, Skinner 2017[a]
Overall rate	12	Ameh 2014, Bardos 2017, Becker 2020, Berglund 2010, Cottrell 2021, Dmello 2021, Dominico 2018, Geelhoed 2018, Mogilevkina 2022, Nolens 2016, Solt 2011, Takeda 2018
Antepartum/before active labour CS	2	Dumont 2018, Sorensen 2010
Emergency CS	1	Sorensen 2010
Intrapartum CS	1	Dumont 2018
CS for prolonged labour	1	Sorensen 2010
CS due to obstructed labor	1	Nolens 2016
Maternal outcomes		
None	6	Becker 2020, Berglund 2010, Dominico 2018, Gulmezoglu 2006, Sorensen 2010, Takeda 2018
Maternal mortality	6	Ameh 2014[b], Dmello 2021, Dumont 2013, Geelhoed 2018, Mogilevkina 2022, Nolens 2016
3rd/4th degree perineal tears	4	Bardos 2017, Cottrell 2021, Solt 2011, Skinner 2017
PPH or transfusion	3	Dumont 2013, Mogilevkina 2022, Skinner 2017
Uterine rupture	1	Nolens 2016
ICU admission	1	Nolens 2016
Overall maternal complication rate	1	Cottrell 2021
Perinatal outcomes		
None	5	Becker 2020, Dominico 2018, Gulmezoglu 2006, Mogilevkina 2022, Takeda 2018
Stillbirths	6	Ameh 2014, Dmello 2021, Dumont 2013, Geelhoed 2018, Nolens 2016, Sorensen 2010
Neonatal deaths	5	Berglund 2010, Dmello 2021, Dumont 2013, Nolens 2016, Sorensen 2010
Perinatal death	1	Nolens 2016
Apgar scores	3	Bardos 2017, Solt 2011, Sorensen 2010
NICU admission	3	Ameh 2014, Berglund 2010, Nolens 2016
Birth injuries	2	Cottrell 2021, Solt 2011
Neonatal resuscitation	2	Berglund 2010, Sorensen 2010
Composite neonatal complications	2	Cottrell 2021, Skinner 2017

ICU – intensive care unit; NICU – neonatal intensive care unit; PPH – post-partum hemorrhage.
[a] Skinner 2017 reported only CS rates in women who attempted AVB. [b]Ameh 2014 reported direct obstetric case fatality rate.

For peer review only

Supplementary file 8. Summary of effects of the interventions in 16 studies included in the review

All percentages indicate baseline x post-intervention rates or incidence. **Bold** terms in the cells indicate rates or outcomes with statistically significant changes after intervention (compared to baseline), according to primary study authors.

Highlights in last column (only for studies with statistically significant increase in overall AVB rate or rate of use of at least one AVB instrument):
Yellow: Non-significant change in rates of adverse maternal or perinatal outcomes.
Green: Significant decrease in rates of adverse maternal or perinatal outcomes.
Grey: Significant increase in rates adverse maternal or perinatal outcomes

Study	Changes in AVB use	Changes in rate of CS	Changes in adverse maternal & perinatal outcomes
Ameh 2014, Kenya	**VE rate: significant increase** (from 0.2% to 1%, at 12 months), P< 0.001	Overall CS rate did not change significantly (21.5% x 21.5%, baseline x post-intervention, Table 40).	MM (Direct obstetric case fatality rate): No significant change<br>**NICU admission for birth asphyxia: significant decrease** (48% mean reduction from 7.6% to 4% at 12 months), P = 0.03<br>**Stillbirth rate: Significant decrease** (35% mean reduction at 12 months from 5% to 3%), , P = 0.03<br>Fresh stillbirth rate: No significant change
Bardos 2017, USA	VE rate: No significant change (3.3% to 3.3%)<br>**Forceps rate: Significant increase** (0.6% to 2.6%, aOR 8.44, 95% CI 3.1–23.1). This effect was seen only during day- time (when senior obstetrician was present).	**Overall CS rate: Significant decrease** (27.3% to 24.5%, aOR 0.68, 95% CI 0.55–0.83). This effect was seen only during day-time (when senior obstetrician was present).	3rd-4th degree tears: no significant change<br>5 min Apgar score <7: no significant change
Becker 2020 USA	Overall AVB rate: no significant changes (from 3.2% to 3.1%)<br>**VE rate: significant decrease** (from 2.0% to 1.2%), P < 0.001<br>**Forceps rate: significant increase** (from 1.2% to 2.0%), P = 0.027	Overall CS rates: increase (no statistical analyses) from 27.9%-30.6% in pre-intervention years to 33.5%-33.9% in post-intervention years	NI

For peer review only

Study	Changes in AVB use	Changes in rate of CS	Changes in adverse maternal & perinatal outcomes
Berglund 2010 Ukraine	**Overall AVB rates**: non- significant difference in 2 sites (Lusk 2% to 2%, Lviv 0% to 0%), **significant decrease in 1 site** (Donetsk from 3.7% to 0%), P<0.0001	**Overall CS**: **significant decrease in two sites** (Donetsk 30% to 18.4%, P<0.0001; Lutsk 33% to 12.7%, P<0.0001). Non-significant change in 1 site (Lviv 21.9% to 16.9%).<br>For 3 sites combined: 29.9% to 15.5% (weighted average, no statistical analysis)	**NICU admission: significant decrease in two sites** (Lutsk 7.3% to 4.4%, P= 0.0115; Lviv 6.4% to 2.4%, P= 0.0115); no significant change in one site (Donetsk)<br>Early neonatal death: no significant change (Donetsk: from 13.6% to 9.5%; Lutsk: from 4.6% to 3.0%; Lviv: 6.4% to 0%).<br>**Neonatal resuscitation: significant decrease** (Donetsk: from 13.1% to 7.4%, P = 0.0025; Lutsk: from 2.4% to 0.9%, P<0.0001; Lviv: from 4.5% to 1.1%, P=0.0003).<br>**Hypothermic infants: significant decrease** (Donetsk from 59.5% to 0.6%, P<0.0001; Lutsk from 85.8% to 0.3%, P<0.0001; Lviv from 77% to 0.7%, P<0.0001).
Cottrell 2021 USA	Overall AVB rate: no significant change (10.5 to 10.5%)<br>AVB failure rate (requiring CS): no significant change (1.8% to 1.4%)<br>**VE rate: significant decrease** (from 8.7% to 6.5%, P = 0.002.)<br>**Forceps rate: significant increase** (from 1.8% to 4.4%, P < 0.001)	Overall CS increased (30.4% to 32.1%)-no statistical analysis	3rd-degree tears: no significant change in forceps or vacuum groups<br>4th-degree tears: no significant change in vacuum group<br>**4th-degree tears: significant decrease in forceps** group (RR 0.1, 95% CI 0.01-0.73)<br>**Overall maternal complication rates: significant decrease in forceps** (RR 0.40 95% CI 0.19−0.75). No significant change in VE group.<br>Facial laceration, scalp injury, cephalo-hematoma: no significant change in forceps or vacuum groups<br>Overall neonatal complications: No significant change in forceps or vacuum groups
Dmello 2021 Tanzania	**VE rate: significant increase** (from 0.03% in 2011 to 3.1% in 2019; RR 1.43; 95% CI 1.41 to 1.43)	**Overall rate of CS**: **significant increase** (from 2.3% in 2011 to 17.6% in 2019; RR 1.15; 95% CI 1.14 to 1.15)	During the decade, facility-based:<br>**MMR: significant decrease** (from 154 to 79 per 100 000 live births, RR 0.92, 95% CI 0.9 to 0.95)<br>**Stillbirth rate: significant decrease** (from 26 to 21 per 1000 live births, RR 0.96, 95% CI 0.96 to 0.97)<br>**Neonatal deaths** (sum of deaths among inborn and referred babies)**: significant increase** (from 5.5 in 2011 to a peak of 14.5) in 2014 and then slightly declined to 12.1 in 2019; RR 1.05, 95% CI 1.04 to 1.05)

For peer review only

Study	Changes in AVB use	Changes in rate of CS	Changes in adverse maternal & perinatal outcomes
Dominico 2018 Tanzania	VE rate: Increase (No statistical analyses)<br>1st yr after intervention: 0.15% to 0.88%<br>2nd yr after intervention: 0.15% to 2.11%<br>3rd yr after intervention: 0.15% to 1.54%<br>4th yr after intervention: 0.15% to 1.26%<br>5th yr after intervention: 0.15% to 1.73%	Overall CS rate: Decrease (No statistical analyses)<br>1st yr after intervention: 11.25% to 10.85%<br>2nd yr after intervention: 11.25% to 9.42%<br>3rd yr after intervention: 11.25% to 9.88%<br>4th yr after intervention: 11.25% to 9.80%<br>5th yr after intervention: 11.25% to 11.18%	NI
Dumont 2013 Mali and Senegal	Overall AVB rate: no significant change (all rates < 3%)<br>Senegal: **Overall AVB**, **significant increase** (1.24% to 2.24%, intervention group, aOR 3.10 (95% CI 1.85-5.20)<br>Mali: Overall AVB, no significant change (3.32% to 1.97% intervention groups, aOR 0.51 (95% CI 0.16 to 1.59)	**Intrapartum CS: significant decrease** (aOR 0.87, 95% CI 0.82 - 0.92, P<0.0001)<br>**Emergency antepartum CS: significant increase** (OR 1.33; 1·19–1·50, p<0·0001) mainly due to PE/E.	**Hospital MM: significant decrease** (OR 0.85, 95% CI 0.73–0.98)<br>Transfusions: marginally significant increase (OR 1·44, 95% CI 0.99–2.11, p=0.06)<br>**Neonatal mortality < 24 h: significant decrease** (adjusted OR 0·74; 95% CI 0.61–0.90)<br>Stillbirth: no significant change
Geelhoed 2018 Mozambique	VE rate: Increase (No statistical analyses)<br>1st yr after intervention:0.2% to 1.0%<br>2nd yr after intervention: 0.2% to 2.3%<br>3rd yr after intervention: 0.2% to 2.2%	Overall CS rate: Decrease (No statistical analyses)<br>1st yr after intervention: 2.7% to 2.1%<br>2nd yr after intervention: 2.7% to 2.1%<br>3rd yr after intervention: 2.7% to 1.8%	Institutional maternal mortality rate: reduction from 126/100,000 births (baseline) to 49/100,000, 48/100,000 (1st yr), 44/100,000 (1st, 2nd and 3rd yr). No statistical analysis<br>Institutional stillbirth rate: reduction from 17.7/1000 newborns (baseline) to 14.9/1000 to 12.4/1000 to 12.2/1000 (1st, 2nd, 3rd yr). No statistical analysis.

For peer review only

Study	Changes in AVB use	Changes in rate of CS	Changes in adverse maternal & perinatal outcomes
Gulmezoglu 2006 Mexico	VE rate: no significant changes in intervention or control groups from baseline to adjusted end-of-study rates (intervention: 0.6% to 0.2% and ctrl: 0.4% to 0.1%)	NI	NI
Gulmezoglu 2006 Thailand	VE rate: no significant changes in intervention or control groups from baseline to adjusted end-of-study rates (intervention: 7.5% to 7.7%, and ctrl: 6.3% to 7.7%)	NI	NI
Mogilevkina 2022 Ukraine	**VE rates: significant increase** 0.1% to 0.7% in intervention group vs 0.11% to 0.26% in the control group (Ratio of the OR: 2.86; 95% CI: 1.80-4.57)<br>**Forceps rates: significant decrease** from 0.17% to 0.08% in intervention group vs 0.21% to 0.05% in the control group (Ratio of the OR: 1.80; 95% CI: 1.00-3.25)	**Overall CS rates: significant increase** (13.12% to 15.9%) in intervention group	MM: non-significant change<br>PPH > 1000 ml: non-significant change<br>Post-partum hysterectomy: non-significant change<br>**Blood transfusion: significant decrease** (OR: 0.56; 95%CI: 0.48-0.65)<br>**Plasma transfusions: significant decrease** (OR: 0.70; 95%CI: 0.63-0.78)<br>**Uterus explorations: significant decrease** (OR: 0.64; 95%CI: 0.59-0.69)
Nolens 2016 Uganda	**VE rate: significant increase** (0.6% to 2.4%, P<0.01)	**Overall CS rate: significant Increase** (28.2% to 30.2%, P< 0.01)<br>CS rate due to obstructed labor: No change (6.0% to 6.0%)<br>Slight change in indications for CS:<br>The percentage of CS for obstructed labour decreased from 23.1% to 20.0% (OR 0.92, 95% CI 0.84-1.01)	**Uterine rupture: significant decrease** (1.1% to 0.8%, P< 0.01; OR 0.75 95% CI 0.61-0.92)<br>Maternal ICU admission due to intrapartum complication: no significant change (1.9% to 1.8%)<br>Maternal death due to intrapartum complication: no significant change (0.3% to 0.2%)<br>Overall maternal deaths: no significant change (0.8% to 0.7%)<br>**Total perinatal mortality: significant decrease** (9.2% to 8.4%, P=0.02)<br>**Intrapartum stillbirths: significant decrease** (3.4% to 2.6%, P< 0.01; OR 0.76 95% CI 0.67-0.85)<br>Term neonatal deaths: no significant change (1.3% to 1.4%)<br>**Term admission to NICU: significant increase** (8.7% to 10.0%, P< 0.01, OR 1.16 95% CI 1.08-1.25)

For peer review only

Study	Changes in AVB use	Changes in rate of CS	Changes in adverse maternal & perinatal outcomes
Skinner 2017 Australia	**VE rates decreased significantly** (from 7.98%-last yr pre-intervention to 6.0%-last yr after intervention)<br>**Forceps rate increased significantly** (from 4.75%-last yr pre-intervention to 9.77%-last yr after intervention)<br>Rate of failed AVB: no significant change	NI (only reported CS in women who attempted AVB)	**PPH: significant decrease** (-1.3, 95% CI -2.07 to -0.49)<br>3rd-4th degree tears: no significant change (-1.04, 95% CI -3.1 to 1.00)<br>Composite neonatal morbidity: no significant change (-0.18, 95% CI -0.38 to 0.02)
Solt 2011 USA	Overall AVB rates. Non-significant change (11.3% to 10.6%)<br>**VE rates: significant decrease** (from 6.4% to 2.9%, P < 0001)<br>**Forceps rate: significant increase** (from 4.9% to 7.8%, P < 0001)	Overall CS: no significant change (25.5% to 27.3%)	3rd and 4th-degree tears: non-significant change (3.6% to 3.1%)<br>5 min Apgar <7 : non-significant change (1.9% to 2.4%)<br>Birth injuries: non-significant change (0.2% to 0.3%)<br>Umbilical cord pH < 7.1: significant decrease (2.4% to 1.5%)
Sorensen 2010 Tanzania	VE rates: No change (0% to 0%).	CS before active labor: no significant change (from 2.0% to 1.8%)<br>Emergency CS: no significant change (from 6.9% to 6.3%)<br>CS for prolonged labor: no significant change (from 5.1% to 4.4%)	**Death of liveborn with Apgar $\geq$ 4: significant decrease** (from 1.1% to 0%, p=0.03)<br>Neonatal deaths: No significant change (1.2% to 0.4%)<br>1-min Apgar <7: No significant change (6.4% to 6.9%)<br>Stillbirths: No significant change (1.7% to 2.5%)<br>Neonatal resuscitation: No significant change (3.0% to 2.4%)<br>Babies with mothers within 10 minutes: significant increase (from 5.6% to 71.5%)

Study	Changes in AVB use	Changes in rate of CS	Changes in adverse maternal & perinatal outcomes
Takeda 2018 Japan	VE rates: decrease (from 1.2%-13.3% in the 10 pre-intervention years to 2.3% after the intervention). No statistical analyses<br>Forceps rate: increase (from 0%-2,0% in the 10 pre-intervention years to 4.3% in post-intervention year). No statistical analyses	Overall CS rates: decrease after intervention (from 7.2%-25.6% in pre-intervention years to 22.2% in post-intervention year). No statistical analyses	NI

aOR: Adjusted odds ratio. AVB: Assisted vaginal birth. CI: Confidence interventional. CS: Cesarean section. Difce: Difference. ICU: Intensive care unit. MM: Maternal mortality. MMR: Maternal mortality ration. NI: No information. NICU: Neonatal intensive care unit. OR: Odds ratio. PPH: Post-partum haemorrhage. RR: Relative risk. VE: Vacuum extraction. Yr: Year

For peer review only

PRISMA 2020 Checklist

Section and Topic	Item #	Checklist item	Location where item is reported
**TITLE**			
Title	1	Identify the report as a systematic review.	p.1
**ABSTRACT**			
Abstract	2	See the PRISMA 2020 for Abstracts checklist.	p.2
**INTRODUCTION**			
Rationale	3	Describe the rationale for the review in the context of existing knowledge.	p.3
Objectives	4	Provide an explicit statement of the objective(s) or question(s) the review addresses.	p.3
**METHODS**			
Eligibility criteria	5	Specify the inclusion and exclusion criteria for the review and how studies were grouped for the syntheses.	p.4-5
Information sources	6	Specify all databases, registers, websites, organisations, reference lists and other sources searched or consulted to identify studies. Specify the date when each source was last searched or consulted.	p.5
Search strategy	7	Present the full search strategies for all databases, registers and websites, including any filters and limits used.	Supl. File 1
Selection process	8	Specify the methods used to decide whether a study met the inclusion criteria of the review, including how many reviewers screened each record and each report retrieved, whether they worked independently, and if applicable, details of automation tools used in the process.	
Data collection process	9	Specify the methods used to collect data from reports, including how many reviewers collected data from each report, whether they worked independently, any processes for obtaining or confirming data from study investigators, and if applicable, details of automation tools used in the process.	p.5
Data items	10a	List and define all outcomes for which data were sought. Specify whether all results that were compatible with each outcome domain in each study were sought (e.g. for all measures, time points, analyses), and if not, the methods used to decide which results to collect.	p.4-5
	10b	List and define all other variables for which data were sought (e.g. participant and intervention characteristics, funding sources). Describe any assumptions made about any missing or unclear information.	p.5
Study risk of bias assessment	11	Specify the methods used to assess risk of bias in the included studies, including details of the tool(s) used, how many reviewers assessed each study and whether they worked independently, and if applicable, details of automation tools used in the process.	p.5-6
Effect measures	12	Specify for each outcome the effect measure(s) (e.g. risk ratio, mean difference) used in the synthesis or presentation of results.	NA
Synthesis methods	13a	Describe the processes used to decide which studies were eligible for each synthesis (e.g. tabulating the study intervention characteristics and comparing against the planned groups for each synthesis (item #5)).	p.6
	13b	Describe any methods required to prepare the data for presentation or synthesis, such as handling of missing summary statistics, or data conversions.	p.6
	13c	Describe any methods used to tabulate or visually display results of individual studies and syntheses.	p.6
	13d	Describe any methods used to synthesize results and provide a rationale for the choice(s). If meta-analysis was performed, describe the model(s), method(s) to identify the presence and extent of statistical heterogeneity, and software package(s) used.	NA
	13e	Describe any methods used to explore possible causes of heterogeneity among study results (e.g. subgroup analysis, meta-regression).	NA
	13f	Describe any sensitivity analyses conducted to assess robustness of the synthesized results.	NA
Reporting bias assessment	14	Describe any methods used to assess risk of bias due to missing results in a synthesis (arising from reporting biases).	NA
Certainty assessment	15	Describe any methods used to assess certainty (or confidence) in the body of evidence for an outcome.	NA

For peer review only

For peer review only - http://bmjopen.bmj.com/site/about/guidelines.xhtml

PRISMA 2020 Checklist

Section and Topic	Item #	Checklist item	Location where item is reported
**RESULTS**			
Study selection	16a	Describe the results of the search and selection process, from the number of records identified in the search to the number of studies included in the review, ideally using a flow diagram.	p.6 and Fig 1
	16b	Cite studies that might appear to meet the inclusion criteria, but which were excluded, and explain why they were excluded.	p.6 and Supl File 2
Study characteristics	17	Cite each included study and present its characteristics.	p.6-7, p.9 Table 1-2 Supl File 3, 5-7
Risk of bias in studies	18	Present assessments of risk of bias for each included study.	p.7, Supl File 4
Results of individual studies	19	For all outcomes, present, for each study: (a) summary statistics for each group (where appropriate) and (b) an effect estimate and its precision (e.g. confidence/credible interval), ideally using structured tables or plots.	p.11-12, Table 3-4, Supl File 8
Results of syntheses	20a	For each synthesis, briefly summarise the characteristics and risk of bias among contributing studies.	NA
	20b	Present results of all statistical syntheses conducted. If meta-analysis was done, present for each the summary estimate and its precision (e.g. confidence/credible interval) and measures of statistical heterogeneity. If comparing groups, describe the direction of the effect.	NA
	20c	Present results of all investigations of possible causes of heterogeneity among study results.	NA
	20d	Present results of all sensitivity analyses conducted to assess the robustness of the synthesized results.	NA
Reporting biases	21	Present assessments of risk of bias due to missing results (arising from reporting biases) for each synthesis assessed.	NA
Certainty of evidence	22	Present assessments of certainty (or confidence) in the body of evidence for each outcome assessed.	NA
**DISCUSSION**			
Discussion	23a	Provide a general interpretation of the results in the context of other evidence.	p.14-15
	23b	Discuss any limitations of the evidence included in the review.	p.15
	23c	Discuss any limitations of the review processes used.	p.15
	23d	Discuss implications of the results for practice, policy, and future research.	p.15-16
**OTHER INFORMATION**			
Registration and protocol	24a	Provide registration information for the review, including register name and registration number, or state that the review was not registered.	p.4
	24b	Indicate where the review protocol can be accessed, or state that a protocol was not prepared.	p.4
	24c	Describe and explain any amendments to information provided at registration or in the protocol.	NA
Support	25	Describe sources of financial or non-financial support for the review, and the role of the funders or sponsors in the review.	p.16
Competing interests	26	Declare any competing interests of review authors.	p.16
Availability of data, code and other materials	27	Report which of the following are publicly available and where they can be found: template data collection forms; data extracted from included studies; data used for all analyses; analytic code; any other materials used in the review.	NA

From: Page MJ, McKenzie JE, Bossuyt PM, Boutron I, Hoffmann TC, Mulrow CD, et al. The PRISMA 2020 statement: an updated guideline for reporting systematic reviews. BMJ 2021;372:n71. doi: 10.1136/bmj.n71

For more information, visit: http://www.prisma-statement.org/

For peer review only - http://bmjopen.bmj.com/site/about/guidelines.xhtml

For peer review only
